# CSACI guidelines for the ethical, evidence-based and patient-oriented clinical practice of oral immunotherapy in IgE-mediated food allergy

**DOI:** 10.1186/s13223-020-0413-7

**Published:** 2020-03-18

**Authors:** P. Bégin, E. S. Chan, H. Kim, M. Wagner, M. S. Cellier, C. Favron-Godbout, E. M. Abrams, M. Ben-Shoshan, S. B. Cameron, S. Carr, D. Fischer, A. Haynes, S. Kapur, M. N. Primeau, J. Upton, T. K. Vander Leek, M. M. Goetghebeur

**Affiliations:** 1 Division of Clinical Immunology, Rheumatology and Allergy, Department of Pediatrics, Sainte-Justine University Hospital Centre, Montreal, QC Canada; 2 Division of Allergy and Clinical Immunology, Department of Medicine, Centre Hospitalier de l’Université de Montréal, Montreal, QC Canada; 3 Research Center of the Sainte-Justine University Hospital Center, Montreal, QC Canada; 4 Division of Allergy & Immunology, Department of Pediatrics, University of British Columbia, BC Children’s Hospital, Vancouver, BC Canada; 5 Division of Clinical Immunology and Allergy, Department of Medicine, Western University, London, ON Canada; 6 Division of Clinical Immunology and Allergy, Department of Medicine, McMaster University, Hamilton, ON Canada; 7 Unit Methods, Ethics and Participation, INESSS, National Institute for Excellence in Health and Social Services, Montreal, QC Canada; 8 Department of Bioethics, School of Public Health of the University of Montreal, Montreal, Canada; 9 Section of Allergy and Clinical Immunology, Department of Pediatrics, University of Manitoba, Winnipeg, MB Canada; 10 Division of Allergy Immunology and Dermatology, Department of Pediatrics, Montreal Children’s Hospital, Montreal, QC Canada; 11 Community Allergy Clinic, Victoria, BC Canada; 12 Department of Pediatrics, University of Alberta, Edmonton, AB Canada; 13 Discipline of Pediatrics, Memorial University of Newfoundland, St. John’s, NL Canada; 14 Department of Pediatrics, Dalhousie University, Halifax, NS Canada; 15 Division of Allergy and Clinical Immunology, Department of Medicine, CISSS Laval, Laval, QC Canada; 16 Division of Immunology and Allergy, Department of Pediatrics, Hospital for Sick Children, University of Toronto, Toronto, ON Canada

**Keywords:** Food allergy, Oral immunotherapy, Clinical practice guidelines, Avoidance, Patient-centered, Evidence, Ethics, Quality of life, Indication, Contraindication, Multi-criteria decision analysis

## Abstract

**Background:**

Oral immunotherapy (OIT) is an emerging approach to the treatment of patients with IgE-mediated food allergy and is in the process of transitioning to clinical practice.

**Objective:**

To develop patient-oriented clinical practice guidelines on oral immunotherapy based on evidence and ethical imperatives for the provision of safe and efficient food allergy management.

**Materials and methods:**

Recommendations were developed using a reflective patient-centered multicriteria approach including 22 criteria organized in five dimensions (clinical, populational, economic, organizational and sociopolitical). Data was obtained from: (1) a review of scientific and ethic literature; (2) consultations of allergists, other healthcare professionals (pediatricians, family physicians, nurses, registered dieticians, psychologists, peer supporters), patients and caregivers; and patient associations through structured consultative panels, interviews and on-line questionnaire; and (3) organizational and economic data from the milieu of care. All data was synthesized by criteria in a multicriteria deliberative guide that served as a platform for structured discussion and development of recommendations for each dimension, based on evidence, ethical imperatives and other considerations.

**Results:**

The deliberative grid included 162 articles from the literature and media reviews and data from consultations involving 85 individuals. Thirty-eight (38) recommendations were made for the practice of oral immunotherapy for the treatment of IgE mediated food allergy, based on evidence and a diversity of ethical imperatives. All recommendations were aimed at fostering a context conducive to achieving objectives identified by patients and caregivers with food allergy. Notably, specific recommendations were developed to promote a culture of shared responsibility between patients and healthcare system, equity in access, patient empowerment, shared decision making and personalization of OIT protocols to reflect patients’ needs. It also provides recommendations to optimize organization of care to generate capacity to meet demand according to patient choice, e.g. OIT or avoidance. These recommendations were made acknowledging the necessity of ensuring sustainability of the clinical offer in light of various economic considerations.

**Conclusions:**

This innovative CPG methodology was guided by patients’ perspectives, clinical evidence as well as ethical and other rationales. This allowed for the creation of a broad set of recommendations that chart optimal clinical practice and define the conditions required to bring about changes to food allergy care that will be sustainable, equitable and conducive to the well-being of all patients in need.

## Background

IgE-mediated food allergy is a condition that imposes food-related restrictions on patients and their caregivers in order to prevent allergic reactions. The burden of disease stems from both the actual and perceived risks of accidental ingestion, including the possibility of a life-threatening allergic reaction. This burden manifests itself in variable levels of anxiety and social limitations and significantly impacts quality of life. The current standard management for food allergy is complete avoidance of the offending allergen in the diet, combined with training on how to recognize and treat allergic reactions. And while avoidance is currently recognized as a safe approach, it has a limited ability to improve a patients’ perception of safety or sense of control over the condition and its associated limitations—this leads many to seek alternative management options [1–6].

Oral immunotherapy (OIT) consists of daily ingestion of the offending food allergen (food dosing), starting below a patient’s threshold dose (i.e. the minimum amount that would elicit a reaction), and increasing the dose over time with a goal of increasing clinical tolerance to that food. It had been proposed as a potential alternative to avoidance throughout the 20th and into the early 21st centuries [7], yet its development had been limited until recently. This is mainly due to the risk of allergic reactions associated with OIT, which is difficult to reconcile with current practice standards that focus on the avoidance of reactions at all cost and are arguably rooted in a culture of fear.

OIT can be viewed as a disruptive innovation as it challenges the current paradigm of care in food allergy and highlights its limitations in responding to patient needs. There is a need for patient-centered ethical clinical practice guidelines (CPGs) that include patients and caregivers as well as other stakeholders in the consultation and deliberation process, in order to develop best practice recommendations for providing OIT for food allergy. It is essential to understand patient and caregiver perspectives to ensure proper interpretation of published evidence and understand the ethical issues involved.

Previous CPGs on OIT [8–10] have used more traditional approaches, focusing mainly on quantitative clinical evidence. However, in order to ensure a clear vision of all that OIT entails and to benefit all patients with food allergy in need of care, CPGs must not only be based on experimental data (e.g., clinical trials) but also on observational, economic and sociopolitical data, as well as experiential narrative data from both patients and healthcare professionals. The optimal provision of OIT must also account for ethical and organizational data to promote the equitable, sustainable and responsible development of this treatment. In fact, evidence-based medicine has been defined as “the application of the best available research to clinical care, which requires the integration of evidence with clinical expertise and patient values [11].”

The development of these CPGs on OIT followed the Guidelines International Network 2015 recommendations including a policy for the management of conflicts of interests [12]. They were commissioned by the Canadian Society of Allergy and Clinical Immunology (CSACI), and was developed through a collaboration with the University Hospital Center Sainte-Justine and the methodological support of the Institut National d’Excellence en Santé et Services Sociaux (INESSS). CSACI acted as the sponsor and was responsible for creating a diversified Working Group. This working group and the executive team opted for a reflective multicriteria methodology, which has been used in the past to develop CPGs [13] using the EVIDEM (Evidence and Values Impact on Decision Making) ethical framework [14–17]. This approach encourages the collection of data from diverse sources, including both the scientific literature and consultations, thus offering a 360° view of the subject. This framework was also used to organize, analyze and ultimately integrate the data into a deliberation guide, providing an efficient and consistent approach throughout the project.

## Materials and methods

### Multidisciplinary team

A multidisciplinary team comprised of experts from a diversity of fields was assembled, including allergists and other healthcare professionals, ethicists, and those with expertise in multicriteria deliberation methodology, literature review, consultations and sociology.

### Multicriteria methodology

The aim of the methodological approach was to support reflection by all stakeholders during the development of fair and reasonable recommendations. To this end, a patient-centered and ethics-based multicriteria framework was used throughout the process. The framework was adapted from EVIDEM, included three additional patient focused criteria and organized 22 criteria into five dimensions defined to cover all relevant aspects of OIT. The framework also builds on the Accountability for reasonableness (A4R) conditions set forth by Daniels and Sabin [18] and the Triple Aim of healthcare systems set forth by Berwick and colleagues [19]. The five dimensions of the framework include:

- How the use of OIT can contribute to a socio-politico-cultural context conducive to The Common Good (sociopolitical dimension),
- How OIT contributes to a fair and equitable distribution of services (populational dimension),
- How OIT responds to a need for health and well-being in an adequate way (clinical dimension),
- How the use of OIT can contribute to improve the quality, organization and governance of healthcare services (organizational dimension), and
- How OIT optimizes the use of resources and the associated costs to ensure sustainability of healthcare systems (economic dimension).

Each dimension contains criteria for operationalization, organized into a multicriteria grid. Data from the literature review and consultations were collected and organized per criterion to allow for a comprehensive assessment of OIT and provide a knowledge platform for the development of the CPG recommendations.

All documentation was compiled using a data management software program (CITAVI), which was adapted to organize data by criteria and provided an interactive interface for review by the working group.

### Conflict of interest management and respect of persons

To avoid the impact of COI or conflicts of roles on the process, explicit guidelines regarding their declaration, evaluation and management were developed based on the GIN 2015 recommendations (see Additional file 1: Appendix 1) [12]. Each potential participant was required to complete a conflict of interest declaration, which was then analyzed by an ethicist. Participants who were deemed at risk of bias on a particular subject were excluded from the room during the deliberation meeting for the time during which the related recommendations were being discussed.

Every step was taken to ensure that best practices for respect of persons were carried out, with respectful atmosphere created by chairs for each group discussion and by interviewers for each individual interview; as well documentation was provided ahead of time to give each participant time to prepare. All patients completed an informed consent; they were informed that only their perspective on the topic was solicited, not personal clinical data, and that psychological support was available.

### Literature review

#### Sources of data

An extensive literature review was performed. The primary sources of data were peer-reviewed full-text publications retrieved through searching multiple sources, including PubMed, MEDLINE (Ovid), Embase, EBM Reviews, CINAHL and Web of Science bibliographic databases. Searches for OIT clinical data were performed on April 17–18, 2019, and searches for epidemiological and burden of disease data were performed on May 30–31, 2019. Search terms are presented in Additional file 1: Appendix 2A. These searches aimed to collect data for all dimensions of the framework, including the sociopolitical, populational, clinical, organizational and economic dimensions. Bibliographical database searches were supplemented by individually searching the tables of contents of pertinent recent periodicals (up to September 2019), as well as bibliographies of key publications. Websites from government agencies, HTA bodies, professional associations and patient associations were also consulted. In order to capture the perception of OIT in the media, a review of the Canadian news media of the last 5 years was conducted via the FACTIVA Global News Monitoring & Search Engine using ‘oral immunotherapy’ and ‘immunothérapie orale’ (French) as search terms.

#### Data selection for each dimension

Retrieved records were screened based on title and abstract to select those for further assessment in full-text format. For the clinical dimension, patient focus groups or surveys providing insight on the importance of the therapy and its outcomes for patients, were considered for inclusion in addition to clinical studies (see details below), qualitative studies, and published CPGs. For the sociopolitical dimension, different types of publications on relevant topics such as the history of OIT and regulatory aspects were reviewed. Stakeholder’s positions were collected and informed by the media press review. For the populational dimension, records such as recent systematic reviews of epidemiological studies, original studies conducted in large, representative populations, preferably in the Canadian context, and recent reviews of qualitative research were considered for inclusion. For the organizational dimension, data concerning the healthcare system’s organization and requirements for the provision of OIT was sought. For the economic dimension, data collected included economic studies, such as cost of illness studies or economic analyses, and cost data (i.e., the costs of products and procedures for OIT). An analysis of associated ethical issues was included for each criterion throughout the framework, as applicable.

#### Selection of clinical studies and assessment of their methodological quality

Clinical studies were selected using eligibility (inclusion/exclusion) criteria listed in Additional file 1: Appendix 2B. Study designs included experimental, comparative studies, randomized [RCTs], non-randomized controlled clinical trials [CCTs], and observational studies reporting outcomes from clinical practice. RCTs are best suited to answer the questions whether an intervention is efficacious and safe, compared to another intervention or management option. These, however, can be limited in their ability to answer certain specific questions that arise in clinical practice, including how patient characteristics may impact outcomes or how to adapt to what happens over the course of treatment [20]. These limits stem from highly selective patient eligibility criteria, rigid study protocols, non-patient-centered end-points, short follow-up durations or small population sizes. A blinded study design, in particular, is not best suited to study personalized care, nor to investigate the impact of a treatment on patient-reported quality of life outcomes—examples include topics such as food-allergy related anxiety and the burden of allergen avoidance, both of which require awareness of the treatment received and the results achieved (e.g., level of desensitization). Observational studies can complement experimental comparative studies, especially when they involve larger less strictly selected patient populations that are closely followed over a long-term horizon in a real world practice setting.

The methodological quality of RCTs was assessed according to the Cochrane risk of bias approach [21], using published risk of bias assessments from systematic reviews, whenever available [22–24]. For case series, the Institute of Health Economics Quality Appraisal of Case Series Studies Checklist was applied [25]. Among its 20 items, 10 core items on study conduct and reporting were selected to operationalize quality assessment. A case series was deemed of high methodological quality (low risk of bias) if all 10 were positive, moderate if 8 to 9 were positive, and low (high risk of bias) if less than 8 were positive. The methodological quality of meta-analyses was determined by checking whether all eligible studies had been included, and by repeating key analyses based on the data reported in the original publications.

#### Extraction, analysis and synthesis

Clinical study data was extracted and synthesised in evidence tables in a per-criterion report format. The extraction tool was validated by a clinical expert. The report was validated by two assessors and then reviewed and revised by the Working Group. Clinical data was further synthesized and integrated into the multicriteria grid for the deliberation. Data pertaining to the other dimensions were directly synthesized in the multicriteria grid.

### Consultations

#### Objective

The objective of the consultation process was to capture relevant experiential and contextual data from diverse perspectives to develop a comprehensive understanding of the topic. Data for each dimension of the multicriteria grid was collected through discussions with allergists, patients and other healthcare professionals. Healthcare professionals were able to provide insight into two main aspects: first, to validate and enrich the data collected through the literature review, and second, enhance the data with relevant clinical practice experience that might not have been elucidated from published studies. Patients with food allergy contributed their unique perspective of the condition and its impact, as well as their view and experience of food allergy therapy, including its benefits and its constraints.

#### General approach

In order to ensure diversity of participants, an open call for applications was posted on the website of applicable patient associations and the CSACI, and targeted calls were made by sending e-mail invitations to relevant professional societies and all CSACI members. The calls provided a short text explaining the objectives of the project, as well as the goals and format of the consultation, followed by a link to a small questionnaire covering the necessary information to enable selecting a diversity of participants to be consulted. The recruitment process was completed by applying a chain-referral sampling strategy. For each potential consultant, the geographical location, gender, general opinion of OIT and, when relevant, the type of practice (e.g. academic vs community, pediatric vs adult) were considered for participant diversification.

Selected participants were contacted and, upon confirmation of participation, asked to complete COI and consent forms. Subsequent steps differed depending on the format of the consultation for each specific group. Possible steps were discussion panels, individual interviews and an online questionnaire. Discussion panels served to create a debate around the various aspects of OIT from different contexts and viewpoints. Individual interviews allowed for the collection of in-depth data from a specific context and its implications. The online questionnaire allowed for the collection of a broad range of experience from a greater number of experts than would have been possible through discussion panels and interviews alone. For every consultation, participants received a consultation guide with questions adapted to their contexts and expertise, which was based on the multicriteria grid and grouped by criteria, in order to facilitate data collection and analysis. All participants were provided with the consultation document a week prior to the consultation in order to give time to familiarize themselves with it. During the discussion panels, the Chatham House Rule [26] was applied, which guarantees anonymity of all information provided by participants. These panels were led by co-chairs experienced in data appropriation while fostering an environment of respect, attentiveness and constructive exchanges.

#### Allergists

Consultations with allergists were twofold, with two different groups. A panel discussion was first conducted with the 15 Working Group members to identify key points on which to focus the CPGs. The group was well diversified in terms of experience with OIT: some participants had never offered it, some had offered it only in research settings, and those offering it in clinical practice reported using different methods and protocols. The discussion followed the multicriteria questionnaire, discussing every issue per criteria. Data was collected through recording and notetaking. Secondly, 42 CSACI members completed an online questionnaire, following the same structure. The data obtained from both the panel discussion and the questionnaire were compiled per criteria. Key themes within each criterion were identified through thematic analysis.

#### Patients and caregivers

Consultations with patients also occurred in two formats to fit two purposes. A panel discussion was held with 8 participants, diversified according to their individual context pertaining to OIT, which included whether or not they had direct experience with it, what their vision of the therapy was and the type and impact of their food allergy condition. Discussions were guided by the multicriteria grid. Patient perspective narrative data was collected for each of the criteria through recording and notetaking. The second consultation format consisted of individual interviews with 6 participants, following the same diversification criteria and using the same questionnaire as the panel discussion. The data from both sources was then compiled in the same way as the allergist consultations and analyzed thematically to identify key points raised by the patients.

#### Other healthcare professionals and lay representatives

These consultations served the purpose of obtaining the viewpoints of stakeholders involved in the care of food allergy patients other than allergists, namely family physicians, pediatricians, nurses, registered dieticians, psychologists and caregivers engaged in peer support activities. They were consulted via a discussion panel involving 10 participants diversified by their professions and the differences in their experience with OIT, which followed the same format as the patient panel. Another two healthcare professionals completed an online questionnaire. Representatives from patient associations were also interviewed, via phone, using the same questionnaire as the panel, to provide patient-centered contextual knowledge on OIT. This data was collected through notes and recording, per criteria, compiled in the same way as for the other consulted groups, and thematically analyzed, bringing forward essential notions associated with OIT provision to include in the CPGs.

### Data from milieu of care

Data from Canadian clinical practices (milieu of care) was collected in the form of economic data provided by the working group members from their clinical practice offer of OIT, which was extracted and compiled into criteria of the economic dimension.

### Deliberation

#### Data integration

Compiled data from the literature review, consultations and milieu of care were integrated into the deliberation guide in a highly synthesized format in order to facilitate comprehension of the data by all participants during the deliberation. Prior to this integration, the data from the literature review was revised and enriched by the Working Group. Data for each criterion was separated into three sections: literature review data, consultation data and ethical aspects. The deliberation guide consisted of the synthesized data integrated into a multicriteria grid, with an additional column in which participants were invited to add their interpretation of the data provided in order to facilitate discussion. Empty recommendation boxes at the end of each dimension were also included to allow discussion and the formation of informed recommendations for one subject at a time.

#### Deliberation process

The participants in the deliberation included the allergists of the Working Group along with selected participants from the consultations, in order to provide relevant data from a global patient perspective. These included two patients with different and longstanding experiences, two family physicians, a pediatrician, a nurse, a registered dietician, a pharmacist and a peer supporter. Each participant received a copy of the deliberation guide 1 week prior to the meeting. The deliberation meeting was chaired by three methodologists who were experts in multicriteria deliberation, multicriteria literature review and synthesis and multicriteria consultations, for a total of 22 participants. The deliberation was divided into four sessions according to the dimensions of the multicriteria grid, during which the literature review and consultation data were presented by the co-chairs. Corresponding recommendations were discussed and determined at the end of each session. All recommendations were based on a group consensus—recommendations for which a COI was identified were based on a consensus of the group excluding those with a COI.

#### Rationales for recommendations

Recommendations were based on a variety of rationales that included evidence from consultations and scientific studies, ethical imperatives, such as promotion of equity or patient autonomy, and other considerations, such as general standards of clinical care, clinical reasoning, and biological plausibility.

For recommendations that included evidence from clinical studies, the strength of the evidence supporting the recommendation was determined using a method that builds on the approach of the Oxford Centre for Evidence-Based Medicine [8, 27]. The risk of bias was assessed using the Cochrane approach [21] and the Institute for Health Economics (IHE) method for case series [25]. These methods, developed for quantitative data, were adapted to include qualitative data from consultations such that the strength of the evidence supporting the recommendation takes into account:

- risk of bias (study design, including the methodology chose for data collection and analysis can affect the risk of misleading results);
- type of study (meta-analyses, RCTs, non-randomized controlled trials (CCTs), case series); studies could be carried out in usual clinical practice or in a research context;
- consistency of evidence across studies; and
- the level of coherence between evidence from studies and data obtained from the consultation process.

The amount, quality and consistency of the evidence supporting the recommendation is defined at three levels:

- *High* Large amount of consistent evidence from RCTs (or meta-analyses) *and* large studies in clinical practice, ideally at a low risk of bias; *AND* coherence with data from consultations and/or qualitative studies.
- *Moderate* Moderate amount of consistent evidence from RCTs (or meta-analyses) and/or studies in clinical practice, ideally at a moderate or low risk of bias; *AND* coherence with data from consultations and/or qualitative studies.
- *Low* Small amount of evidence *OR* evidence with some incoherence in data from RCTs (or meta-analyses) and/or studies in clinical practice *OR* data at moderate to high risk of bias; *AND* coherence with data from consultations and/or qualitative studies.

*Strength of recommendations* The strength of recommendations in CPGs is often graded based on the quality of clinical evidence regarding the efficacy and safety of an intervention. However, this approach does not apply to recommendations that do not rest on clinical trial outcomes, but for which the body of evidence from clinical research and practice shows a clear clinical benefit because conducting such trials would neither be reasonable nor ethical [28]. Moreover, grading in such a way does not take into account factors other than clinical outcomes, such as ethical imperatives, social context or economic considerations, which can be key elements of the rationale underlying a recommendation. Therefore, to ensure that all types of recommendations in these CPGs will be regarded on an equal footing, the strength of recommendations was not given a rating. Rather, in the spirit of accountability for reasonableness (A4R) [18], the rationale for each recommendation, the level of supporting evidence, where appropriate, and the necessary contextualization and nuances were all clearly stated.

## Results

### Multicriteria grid

**Fig. 1:**
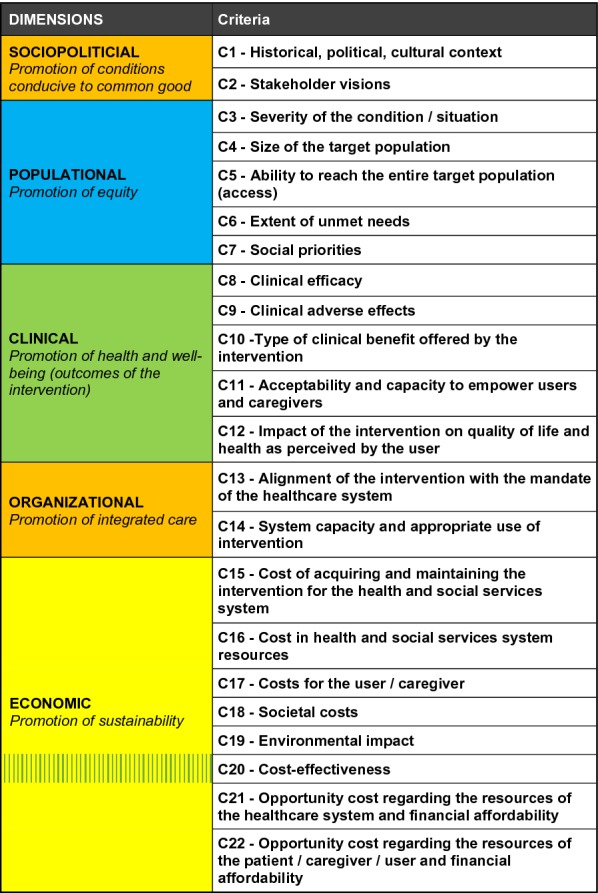
Multicriteria grid: dimensions and criteria

The multicriteria grid used for this project included five dimensions divided into 22 criteria and is shown in Fig. 1.

### Data used as basis for recommendations

**Fig. 2:**
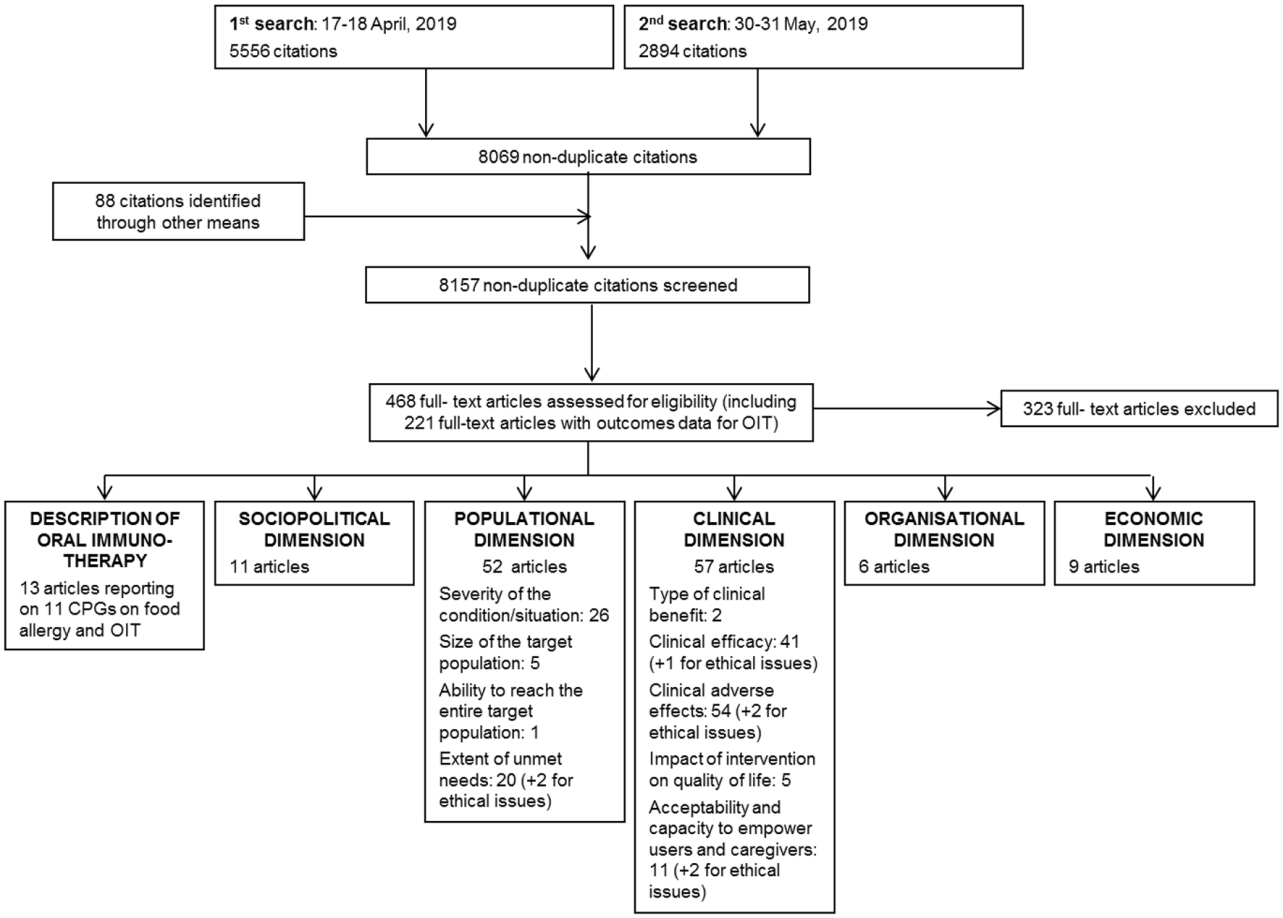
PRISMA diagram

The literature review yielded a total of 8157 records; 468 of them were assessed for eligibility in full-text records and 145 were included in the multicriteria grid (Fig. 2). An additional 17 articles were included from the media press review.

A total of 14 patients or caregivers, 13 allergists and 16 other healthcare professionals or patient association representatives were consulted through panel discussions or individual interviews. In addition, 42 CSACI allergists responded to the online consultation survey.

Data on the economic aspects of OIT was available from three Canadian practices, and data on quality of life impact of OIT was collected from one practice.

The synthesis of the data collected through the literature review, consultations and from the milieu of care is presented by criteria along with complete references in the deliberation guide (Table 1). Detailed clinical evidence tables with results of quality assessments are available in Additional file 1: Appendix 3.

**Table 1:**
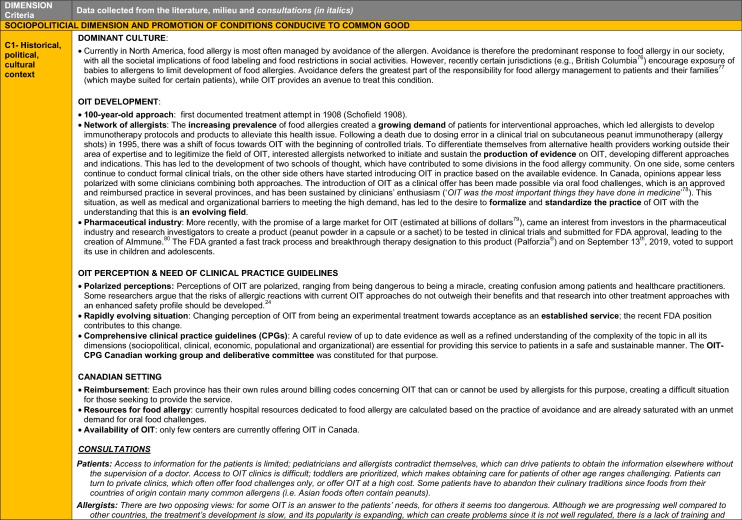

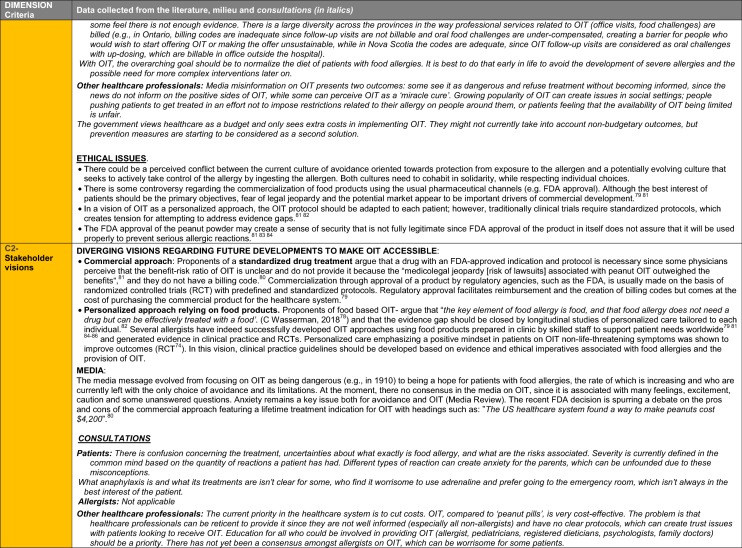

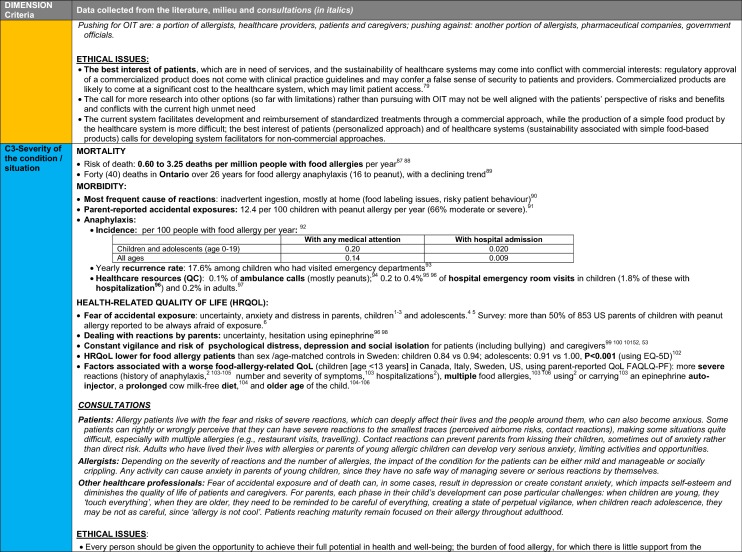

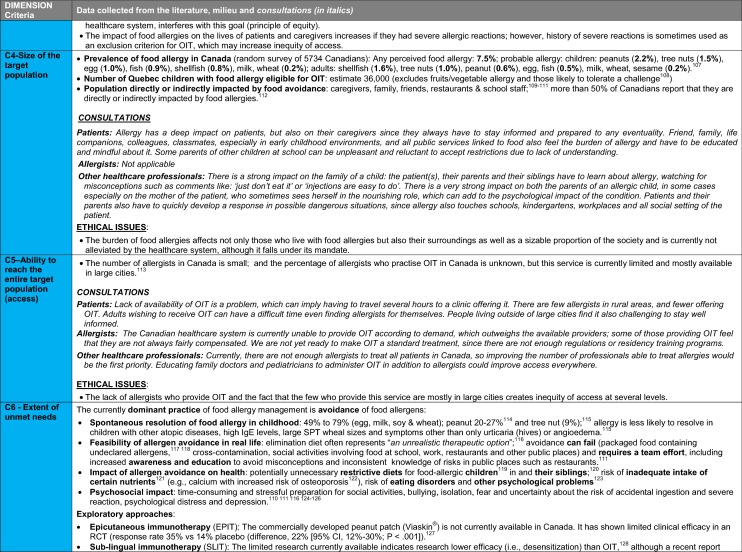

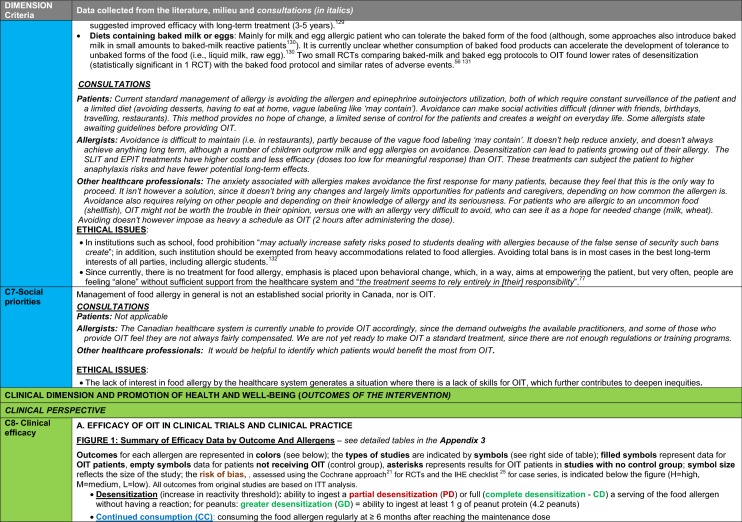

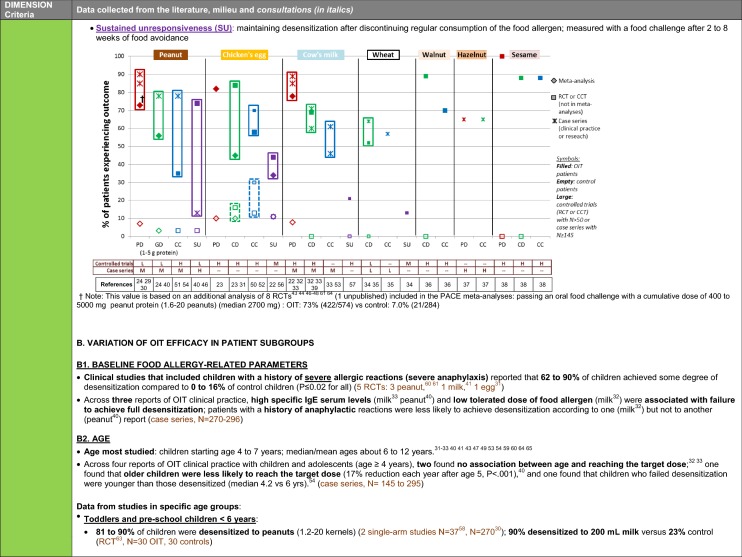

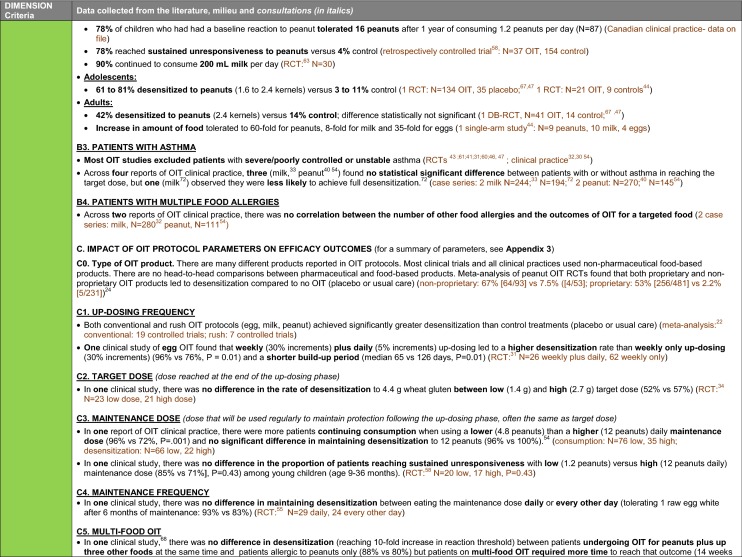

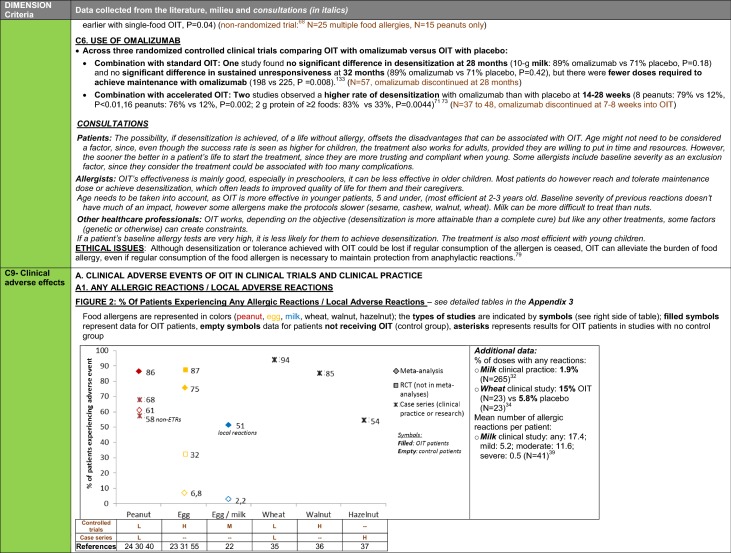

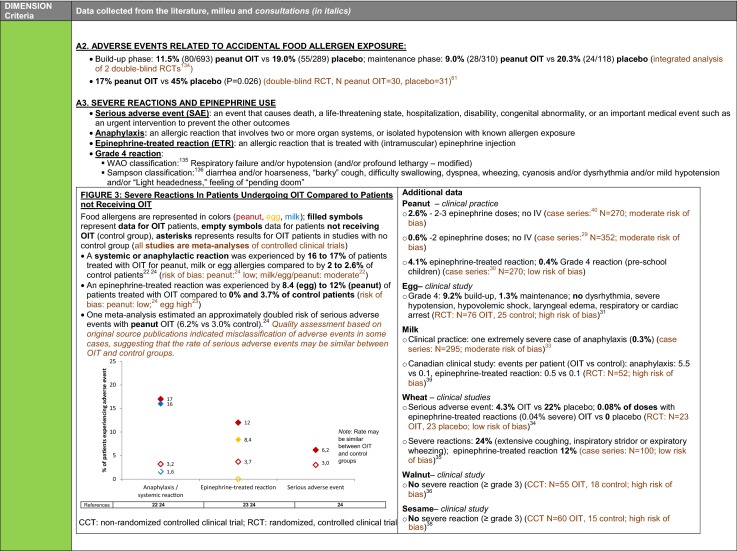

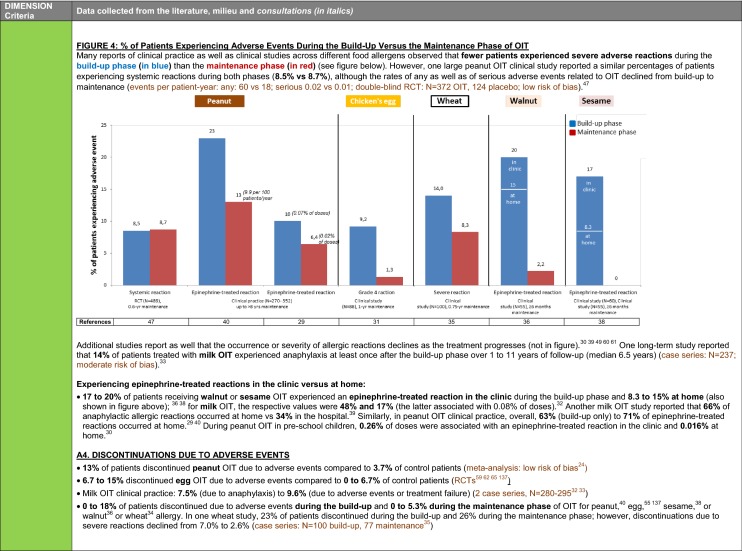

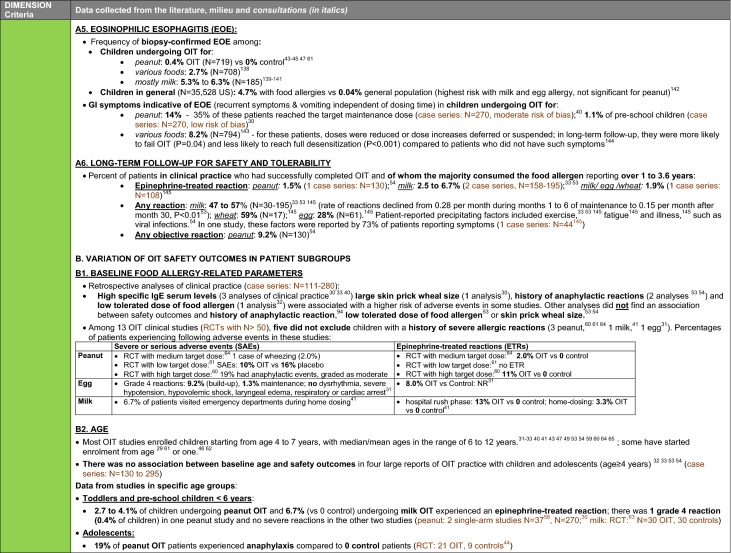

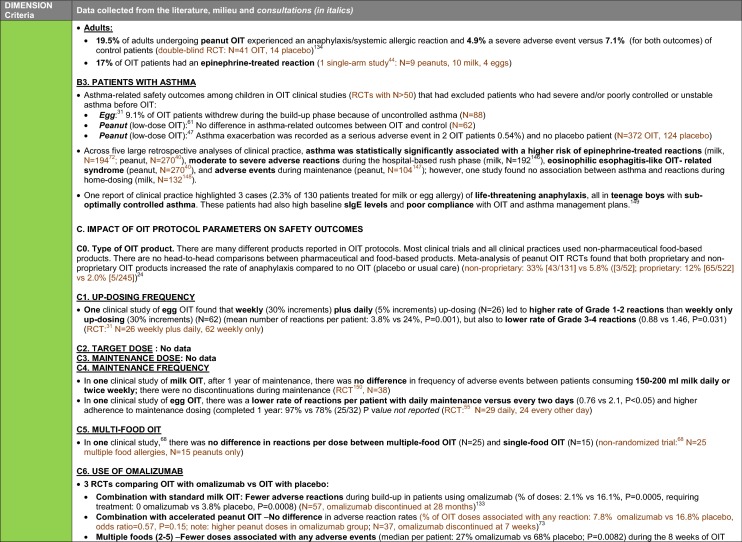

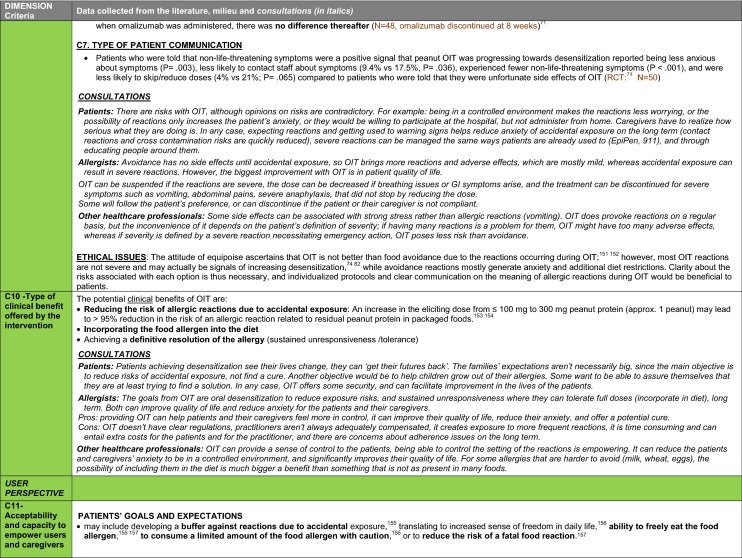

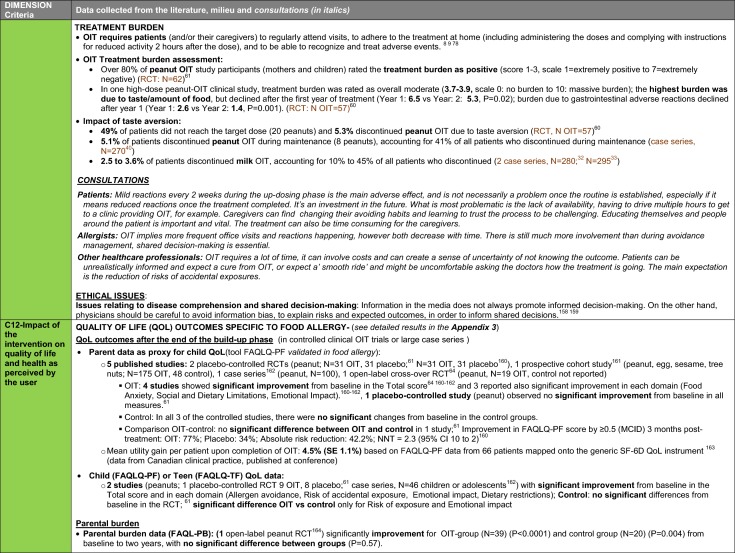

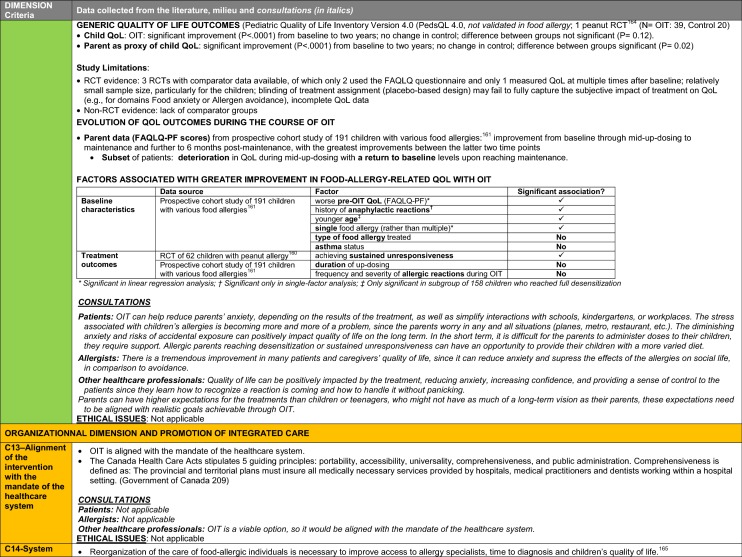

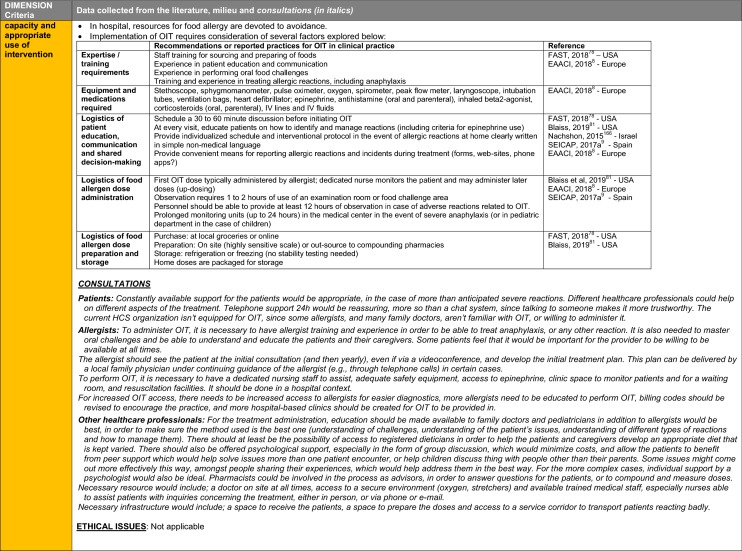

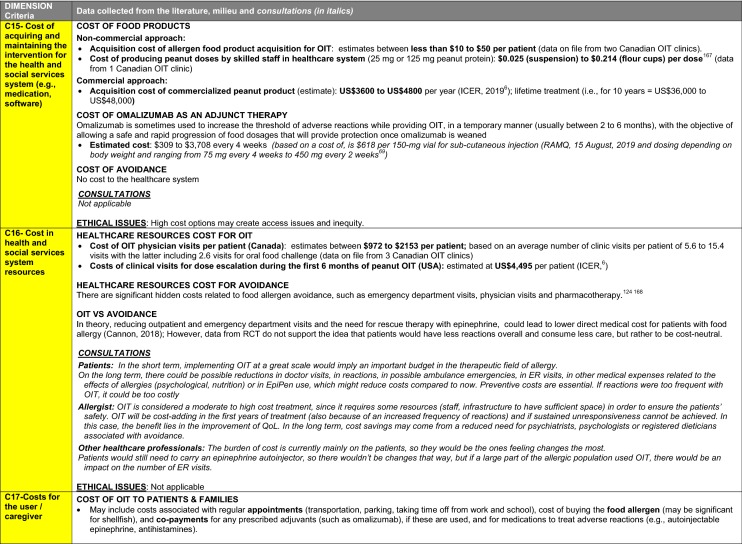

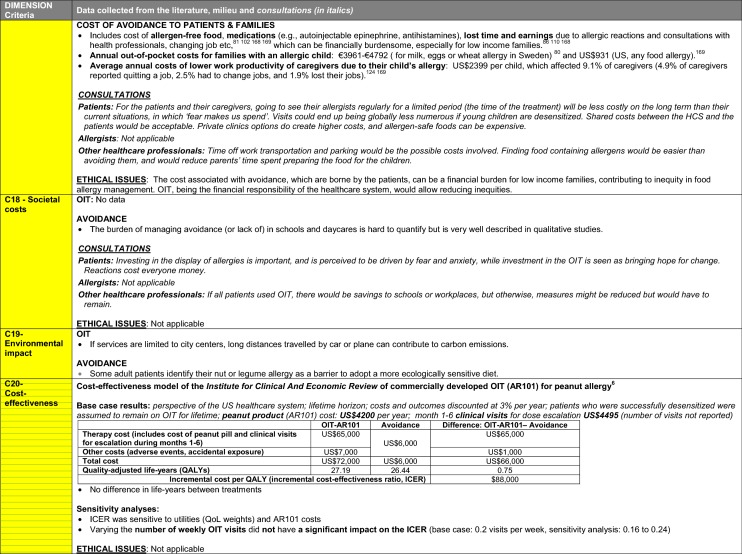

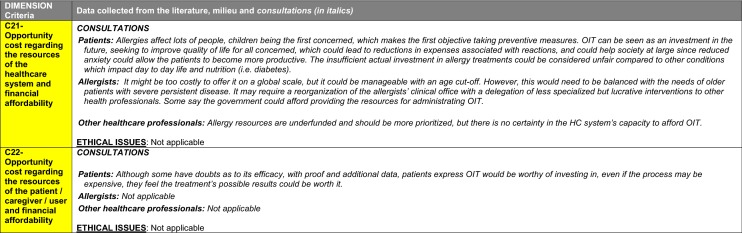
Multicriteria grid with data from literature review, consultations and milieu of care synthesized by criteria and used for the deliberation

### Deliberation guide

The deliberation guide includes the synthesized data for each criterion along with a section prompting participants to interpret and discuss the data. Boxes for the development of recommendations were added at the end of each dimension.

### Recommendations

Recommendations are presented in the following format:

1. A lay summary of the data synthesis that led to the recommendation(s) written in a format that is accessible to a non-physician audience (for the clinical dimension, detailed narrative summaries of the data are available in Additional file 1: Appendix 4).
2. Additional key points discussed during deliberation that led to the recommendation(s)’ development.
3. Recommendations and rationales.

**Fig. 3:**
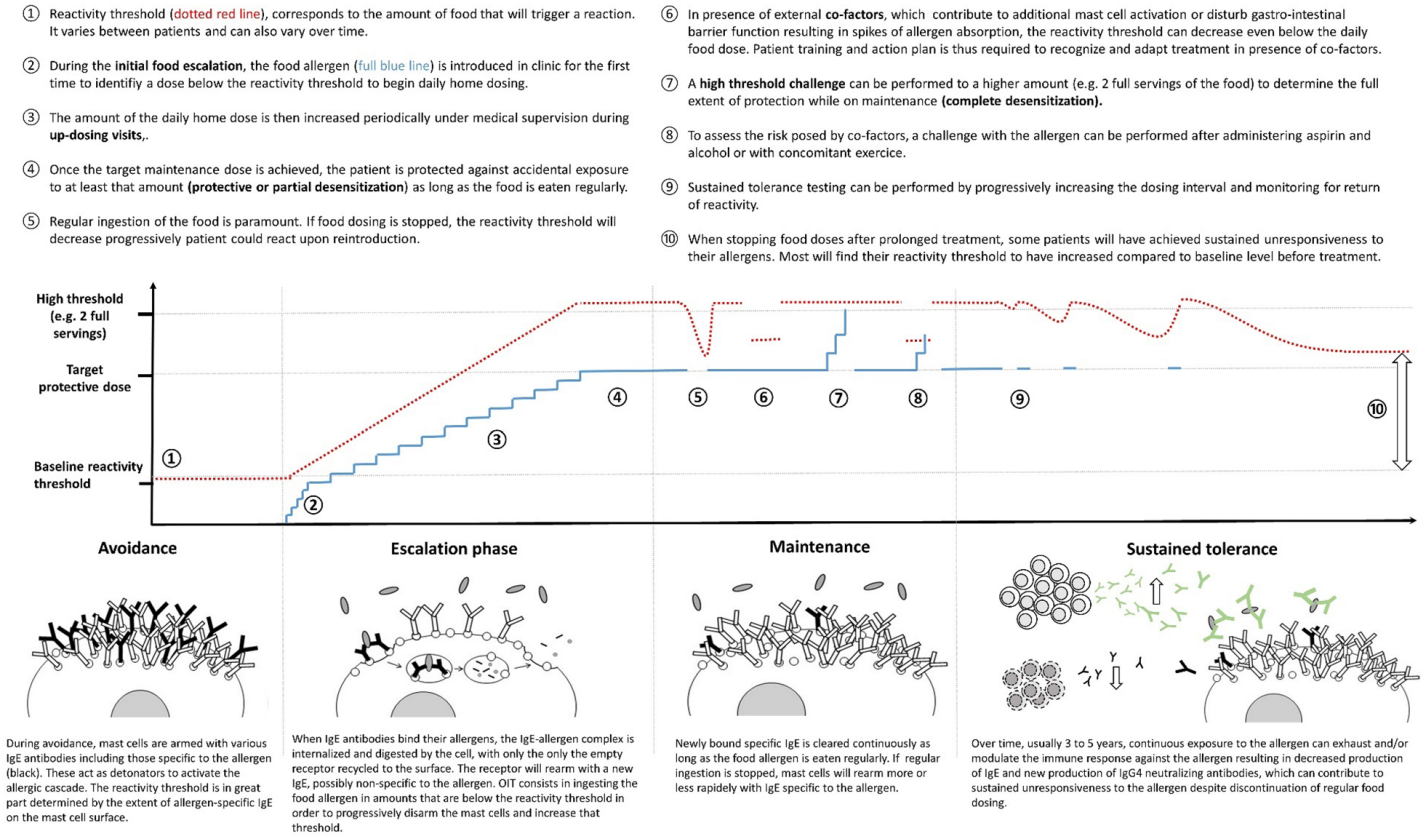
Key concepts and definitions pertaining to OIT

To facilitate the understanding of the presented data, key concepts and definitions pertaining to OIT are illustrated in Fig. 3.

### Sociopolitical dimension: promotion of the common good

Recommendations for a sociopolitical context for optimized food allergy management *Summary of data synthesis* (*see details and references in* Table 1, *Sociopolitical dimension, criteria 1 and 2*): Data collected for the sociopolitical dimension illustrates that the main practice in food allergy management is avoidance, in which patients and caregivers carry most of the responsibility. Although this management method is certainly appropriate for some, there has been a growing demand for an interventional approach in food allergy, leading to recent developments in OIT. With OIT treatment growing in popularity, two opposing perceptions have arisen: one of fear and one of hope. Confusing misinformation concerning risks and benefits of OIT further contributes to a polarizing discussion surrounding OIT. Within this unconducive context, OIT has only recently been introduced in a limited number of clinical practices. Access disparities are further exacerbated by increasing demand, the lack of specialized care in urban and, even more so, rural areas, and a potentially inadequate billing system in some Canadian provinces. Additionally, some proponents assert that OIT should only be administered using pharmaceutical products, such as pre-packaged food doses, indented as a life-long treatment, rather than using readily available food for OIT, creating issues that could adversely affect both patients and sustainable development of OIT.

**Additional key points from deliberation**

It is essential that patients with food allergy have the ability to control the management of their condition so that it reflects their needs and objectives. As such, they should be able to access OIT when it is requested. Patients will be able to reach these objectives more effectively when adequately informed about their treatment, to manage anxiety related to food allergy and to prevent unrealistic expectations about the benefits of OIT or an overestimation of its risks. A fully standardized approach would not be adequate in this context since it cannot respond to each patient’s situation and needs—this calls for research designs on food allergy care to be directed towards answering key questions of personalized care, such as long-term outcomes and real-life needs.

**Table:** 

Box 1: Recommendations for a sociopolitical context for optimized food allergy management	Ethical imperative, data or other considerations in support of the recommendation
A large number of patients with food allergies are unable to access basic care for proper diagnosis and management, including avoidance or oral immunotherapy, leaving many with inadequate support from the healthcare system. Empowerment of patients and families should be promoted through shared responsibility with the healthcare system, in respect and support of patient choices	This recommendation is based on the principle of *solidarity* of public healthcare systems towards patients to provide necessary care and foster patient and family *autonomy* in their choices and management of their condition It is supported by data from consultations with stakeholders and key aspects emerging from the literature
A new culture should be fostered to transition from one that is fear-driven towards one that promotes a sense of control in food allergy through accurate information on the condition and the options available	This recommendation is based on the principle of *patients’ best interest*, as defined by the patients themselves It is supported by data from consultations with stakeholders and key aspects emerging from the literature
There is inadequate reliable communication between patients, families and healthcare professionals about oral immunotherapy, often accompanied by misinformation. This should be addressed through shared decision-making, and access to trustworthy, clear and transparent information	This recommendation is based on the principle of *transparency* to ensure easy access to all relevant and trustworthy knowledge on OIT and safeguard patient and family decisional *autonomy* It is supported by data from consultations with stakeholders and key aspects emerging from the literature
Oral immunotherapy for food allergy should be developed and practiced in the spirit of personalized care, considering the heterogeneity and specificity of the condition and individual patient contexts	This recommendation is rooted in the principle of *patient protection*, since failure to adapt to the patient and their individual characteristics can lead to risky or futile interventions It is supported by data from consultations with stakeholders and key aspects emerging from the literature
Research should be encouraged to adequately inform clinical practice regarding OIT, through innovative study designs focusing on meaningful patient-centered and long-term outcomes, while appropriately reflecting the need for personalized care in real-world practice	This recommendation is rooted in the principle of *patient autonomy*. To ensure informed decision making in OIT, research needs to focus on meaningful patient-centered and long-term outcomes that are coherent with individual realities and objectives It is supported by data from consultations with stakeholders and key aspects emerging from the literature It is supported by the previous recommendation for the development and practice of OIT in the spirit of personalized care

### Populational dimension: promotion of equity

Recommendations for the equitable provision of OIT *Summary of data synthesis (see details and references in* Table 1, *Populational dimension, criteria 3 to 7):* The burden of food allergy can be described in two ways: based on the risk of severe reactions which is relatively low or based on its impact on the psychosocial wellbeing of patients and caregivers, which is extensive (see details and references in Table 1). Food allergy affects many spheres of life for patients and their caregivers and may lead to isolation or vulnerability to bullying. These impacts may be lifelong for those in whom food allergy does not resolve naturally with time.

A management strategy based solely on avoidance is often characterized by a number of unmet needs, as patients and caregivers are often left to manage their condition by themselves. Society can help reduce risk and burden with measures such as appropriate food labeling regulations or food restriction/management protocols in schools and restaurants. However, even with these measures in place, patients and caregivers will continue to have many limitations and few options to establish control over their risk of reaction.

Up to 50% is directly or indirectly affected by food allergy, according to some estimates, including patients, family and caregivers, their immediate social circles and society. Despite this, there is low investment in this area by the healthcare system, with a low capacity of care for both accurate diagnosis and management. There are disparities in access to specialized care, which is generally limited, both in urban and rural areas.

**Key additional points from the deliberation**

A culture of fear currently exists regarding food allergy and includes widespread erroneous concept that all allergies should be considered equally dangerous. As a result, many believe that the only acceptable management option is strict avoidance. Instead, the risk of reaction is extremely variable and hard to predict. This results in a disparity between populational epidemiological data and actual individual risk. Individuals vary with respect to their level of comfort with risk as well as their perception of the extent of benefit derived from a treatment. Thus, the decision to pursue OIT should be left to the well-informed patient as much as clinically possible, rather than based on external criteria.

It was strongly felt that in the development of OIT within the healthcare system as an option of care, steps must be taken to ensure that resources in food allergy are not disproportionately displaced toward OIT, so as to ensure that patients will continue to receive optimal care if opting for the traditional approach of food avoidance.

**Table:** 

Box 2: Recommendations for the equitable provision of OIT	Ethical imperative, data or other considerations in support of the recommendation
The notion of severity is inadequate for determining eligibility for OIT, as the risk of a reaction and of it being severe are difficult to predict and does not necessarily correlate with the psychosocial impact on patients and families. The aim should thus be to make OIT available as an option for all patients wishing to receive it, provided there is no contraindication and a clear understanding of individual risks and benefits	This recommendation is based on the principle of *equity in eligibility* It is supported by data from consultations with stakeholders and key aspects emerging from the literature
A lack of public investment has resulted in a situation of low capacity and disparities in access to care for the accurate diagnosis and proper management of food allergy. This applies to management choices that include both avoidance and OIT	This recommendation is based on the principles of *solidarity and equity of access*. This refers to both regional access and access to food allergy care in general It is supported by data from consultations with stakeholders and key aspects emerging from the literature

### Clinical dimension: promotion of health and well-being

Recommendations on eligible food allergens and types of clinical outcomes that can be achieved by OIT *Summary of data synthesis (see details and references in* Table 1, *clinical dimension, criteria 8 to 12):* Many studies with OIT to a variety of different food allergens show that a majority of patients can achieve protective levels of desensitization and that a substantial proportion can achieve complete desensitization, which can be maintained with continued consumption of the food. Studies show that many patients do continue consuming the food allergen regularly in the longer term. A variable smaller proportion can maintain tolerance to the food allergen even after a period of prolonged avoidance; however, this data is scarce and only available for the major food allergens.

Patients undergoing OIT will more likely experience allergic reactions than those who are avoiding the food. This is because treatment dictates that they intentionally consume their food allergen. A majority of OIT patients have at least one allergic reaction, varying mild to severe (i.e. anaphylaxis), and some may require the use of epinephrine. According to many studies across different designs and food allergens, the frequency and/or severity of allergic reactions declines as the treatment progresses. A few studies also observed that the frequency of reactions from accidental exposure declines in patients undergoing OIT.

Even after a prolonged period of maintenance dosing, systemic reactions may occur in some patients. These reactions usually occur in the presence of a cofactor (e.g. exercise, sleep deprivation, illness and fever, certain medications) that transiently increases the likelihood of reaction, but the risk appears to decrease with longer time on maintenance.

While some patients ultimately discontinue OIT due to adverse events, other factors may also result in discontinuations and include anxiety or refusal to ingest the food doses.

**Additional key points from deliberation**

Although reactions are more frequent during OIT than with avoidance in the short term, the anxiety associated with these is generally manageable because they are expected and can be planned for, which contributes to patients’ sense of control. The burden of these reactions is generally offset by the protection granted against unexpected reactions from accidental exposures outside of food dosing, which creates a sense of empowerment by lifting the burden of constant vigilance and provides a sense freedom. For many patients, the burden of these reactions diminishes over time as they learn to self-manage.

The variability in safety outcomes between studies may be due to various factors, including a treatment center’s experience in OIT. This will affect the ability of providers to personalize treatment plans, shaping the instructions offered to patients on how to take the dose and manage reactions.

**Table:** 

Box 3: Recommendations on eligible food allergens and types of clinical outcomes that can be achieved by OIT	Ethical imperative, data or other considerations in support of the recommendation *Level of evidence (applicable when recommendations are based on outcome data from clinical studies)*
There is no convincing evidence of a clinically significant difference between food allergens in terms of safety and efficacy outcomes in OIT for the treatment of IgE-mediated food allergy. Therefore, all recommendations in these CPGs are generally applicable to all food allergens, unless there is specific evidence to demonstrate otherwise	This recommendation is based on the principle of *equity of eligibility* It is supported by *large amount of consistent clinical evidence*, considering the absence of demonstrated lack of efficacy or of a consistent safety issue for any specific food despite a large number of clinical studies for a variety of foods [22–24, 29–38]. *Level of evidence: HIGH* It is also supported by the lack of *biological plausibility* that the mechanism of OIT would differ from one allergen to another.
OIT is recommended as a treatment to achieve desensitization. A majority of patients will achieve a level desensitization to a daily dose of the allergen that will be sufficient to provide protection against trace exposure, while a sizable proportion of patients will be able to tolerate a full serving	This recommendation is based on the principle of *beneficence*. Whether or not the outcomes (i.e. desensitization, continued consumption or sustained unresponsiveness) are worth pursuing remains patients’ prerogative, in line with the principle of *patient autonomy* It is supported by a *large amount of consistent clinical evidence*, which includes three meta-analyses [22–24] (together covering 31 published OIT controlled clinical trials), three additional RCTs [31, 34, 39] and two non-randomized controlled clinical trials (CCTs) [36, 38], five large case series in clinical practice (N > 150) [29, 30, 32, 33, 40], and two smaller case series [35, 37]. This body of evidence includes 10 RCTs rated as being at low risk of bias [34, 41–49] and is coherent with data from consultations. *Level of evidence: HIGH*
OIT can be recommended for long term management since a sizable proportion of patients will continue to regularly consume a sufficient amount of the food to maintain desensitization after reaching maintenance, without reverting to complete avoidance	This recommendation is based on the principle of *beneficence*. Whether or not the outcomes (i.e. desensitization, continued consumption or sustained unresponsiveness) are worth pursuing remains patients’ prerogative, in line with the principle of *patient autonomy* It is supported by a *moderate amount of consistent clinical evidence*, from three long-term, 2-arm follow-up studies of RCTs (high risk of bias due to open-label; mean follow-up 2.5 to 5 years; completeness of follow-up: 77 to 90%) [50–52], three large case series in clinical practice (N ≥ 145) (moderate risk of bias using the IHE tool due to single-center, retrospective design; median follow-up: 1.5 to 6.5 years; completeness of follow-up: 83 to 99%) [33, 53, 54], and four smaller single-arm, follow-up studies (N = 43 to 100) at low to high risk of bias (completeness of follow-up: 82 to 100%) [35–37, 55]. This is coherent with data from consultations. *Level of evidence: MODERATE*
OIT may be recommended to achieve sustained unresponsiveness, but data is limited and variable	This recommendation is based on the principle of *beneficence*. Whether or not the outcomes (i.e. desensitization, continued consumption or sustained unresponsiveness) are worth pursuing remains patients’ prerogative, in line with the principle of *patient autonomy* It is supported by a *small amount of evidence with some incoherence in findings*, including one meta-analysis (covering 4 RCTs) [22], three additional RCTs [34, 46, 56], one CCT [57], one prospective trial with retrospectively- matched controls [58] and one case series in clinical practice [40]. This is coherent with data from consultation concerning pre-school children. *Level of evidence: LOW*

Recommendations on who could benefit from OIT (indications) *Summary of data synthesis (see details and references in* Table 1, *Clinical dimension, criteria 8 to 12):* The allergists consulted highlighted the need for accurate diagnosis of food allergy before initiating treatment, since many patients are either mislabeled or wrongly believe they are allergic.

Most OIT studies included children and adolescents across a wide age range. Several report that the treatment is very efficacious and well tolerated in toddlers and preschoolers. A few studies focused on adults and suggested that they can also achieve desensitization.

**Additional key elements from deliberation**

An accurate diagnosis of IgE-mediated food allergy may require an oral food challenge, especially if the patient does not have a convincing medical history or a high food-specific IgE level. In addition to confirming the diagnosis, an advantage of performing a food challenge prior to starting OIT is that it can inform the initial escalation dose. This advantage must be weighed against other practical considerations, including the burden of inducing a highly probable allergic reaction and available resources to perform food challenges.

Toddlers and preschoolers are an attractive group for OIT due to the high efficacy and relative ease of treatment in this group. However, it is important that efforts are not focused solely on an age group where treatment appears to be more cost-effective, as specific clinical expertise should also be developed to address the need in other age groups, including adults. A careful balance must thus be found between the promotion of sustainability and the promotion of equity of access.

**Table:** 

Box 4: Recommendations on who could benefit from OIT (indications)	Ethical imperative, data or other considerations in support of the recommendation *Level of evidence (applicable when recommendations are based on outcome data from clinical studies)*
An accurate diagnosis of IgE-mediated food allergy is essential before proceeding with OIT	Regardless of therapeutic option considered, accurate diagnosis of food allergy is the basis for proper care to avoid futile treatment, including unnecessary avoidance. This recommendation is thus based on the principles of *nonmaleficence* and *sustainability*
OIT is indicated for toddlers and preschoolers *Important consideration* While the likelihood of spontaneously outgrowing milk or egg allergy may be greater than for other foods, their impact on patients and families, if not outgrown, is high. Caregivers should be included in shared decision-making about, whether to initiate OIT early for these foods and based on individual prognosis, considering that OIT is well tolerated and has high efficacy in this age group	This recommendation is based on the principle of *equity in eligibility* as well as *proportionality* between risks and benefits, considering patient’s goals and perspectives For *desensitization*, it is supported by a *large amount of consistent clinical evidence*. Many OIT studies (RCTs [34, 41, 43, 47, 49, 59, 60] as well as large clinical practice case series [32, 33, 40, 54]) enrolled children starting from the age of 4 or 5 years; some have started enrolment from age three [29, 61] or one [46, 62]. In addition, there is a moderate amount of consistent evidence specifically for this age group from one RCT of milk OIT (unclear risk of bias—Cochrane) [63], one large (N = 270) prospective, multi-center case series in clinical practice of peanut OIT (low risk of bias—IHE tool) [30] and one small (N = 37) prospective, uncontrolled clinical trial of peanut OIT [58]. This is coherent with data from consultations. *Level of evidence: HIGH* For *sustained unresponsiveness*, it is supported by a *small amount of clinical evidence* (1 prospective clinical trial of peanut OIT with retrospectively matched controls observing a large effect size [58]) and is overall coherent with data from consultations. *Level of evidence: LOW*
OIT is indicated for school-age children and adolescents	This recommendation is based on the principle of *equity in eligibility* as well as *proportionality* between risks and benefits, considering patient’s goals and perspectives. For *desensitization*, it is supported by a *large amount of consistent clinical evidence*. Most of the evidence for desensitization stems from studies that enrolled children and adolescents with median/mean ages in the range of 6 to 12 years (RCTs [31, 34, 39, 41, 43, 46, 49, 59–61, 64, 65] and large clinical practice case series [32, 33, 40, 54]) In nine RCTs [34, 39, 41, 43, 44, 47, 59, 61, 64] and five large clinical practice case series [29, 32, 33, 40, 54], the upper age limit for enrolment was 16 years or older (up to 27 years [32]). This is coherent with data from consultations. *Level of evidence: HIGH* For *sustained unresponsiveness*, it is supported by a *small amount of consistent clinical evidence*. Most of the limited evidence that is available for sustained unresponsiveness stems from studies that enrolled children with median/mean ages in the range of 6 to 9 years (RCTs [34, 46, 49, 56, 59, 66], CCT [57] and clinical practice case series [40]). *Level of evidence: LOW*
OIT may be indicated for adults	This recommendation is based on the principle of *equity in eligibility* as well as *proportionality* between risks and benefits, considering patient’s goals and perspectives. For *desensitization*, it is supported by only a *small amount of consistent clinical evidence*. The limited data available for patients 18 years of age and older, from one double-blind RCT that included a small number of adults [47, 67] and one small case series [44] suggest that desensitization is possible in this age group. *Level of evidence: LOW*

Recommendations regarding contraindications *Summary of data synthesis (see details and references in* Table 1, *Clinical dimension, criteria 8 to 12):* A history of anaphylactic reactions to the targeted food allergen was generally not an exclusion criterion in OIT studies. Some studies suggested that a history of anaphylaxis had an impact on OIT outcomes, but most of these patients were able to achieve at least partial desensitization. The number of food allergies a patient has appears to have no effect on treatment efficacy in terms of reaching the target maintenance dose to one food.

Most OIT studies included patients with controlled asthma, but generally excluded patients with uncontrolled or severe asthma (i.e. severe asthma defined as the requirement for extensive medication to achieve control). Patients with asthma tended to have a higher risk of adverse reactions and, in certain studies, some experienced a worsening of their asthma. Nevertheless, most patients with controlled asthma were able to achieve at least partial desensitization.

OIT requires that patients and their caregivers attend visits regularly and are able to understand and follow instructions regarding how to administer the treatment at home. Patients must also be able to recognize and treat adverse events.

**Additional key elements from deliberation**

Successful OIT requires a significant commitment from both patients and caregivers – it is essential to ensure all are informed and understand the importance of this commitment. Psychological support can help if there is significant anxiety or other psychological issues and should ideally be sought before beginning OIT.

**Table:** 

Box 5: Recommendations regarding contraindications	Ethical imperative, data or other considerations in support of the recommendation *Level of evidence (applicable when recommendations are based on outcome data from clinical studies)*
Previous history of anaphylaxis to the targeted food is not a contraindication for OIT	This recommendation is based on the principle of *equity in eligibility* It is supported by a *large amount of clinical evidence* A history of anaphylactic reactions to the targeted food allergen was generally not an exclusion criterion in OIT studies. Some RCTs [43, 46, 47, 49, 59, 63] and clinical practice case series [30] excluded patients with a history of *severe* anaphylaxis; others included them (RCTs [41, 61, 64]; case series [29, 40]). Evidence from large case series on whether baseline history of anaphylaxis had an impact on OIT outcomes is inconsistent [32, 40, 53, 54], but most patients with a history of anaphylaxis were able to achieve at least partial desensitization [32, 40]. This is coherent with data from consultations. *Level of evidence: HIGH*
Multiple food allergies are not a contraindication to OIT	This recommendation is based on the principle of *equity in eligibility* It is supported by a *moderate amount of consistent clinical evidence* from two case series (N = 111 and N = 280; moderate risk of bias [IHE tool] [32, 54]) and additional clinical studies targeted towards patients with multiple food allergies [68–71]. *Level of evidence: MODERATE*
Uncontrolled asthma is an absolute contraindication to OIT. Asthma must be controlled before beginning OIT and pro-actively managed during OIT	This recommendation is based on the principle of *nonmaleficence* In many RCTs [31, 39, 41, 43, 46, 47, 60, 61] and clinical practice case series [30, 32, 54], severe and/or poorly controlled or unstable asthma was an exclusion criterion for OIT. However, most patients with controlled asthma were able to achieve at least partial desensitization [33, 40, 54, 72]. This is coherent with data from consultations. *Level of evidence: HIGH*
Pregnancy is an absolute contraindication for initiating OIT	This recommendation is based on the principle of *nonmaleficence* It is in line with the current general standard of care in allergen immunotherapy
Conditions such as active severe atopic dermatitis, pre-existing eosinophilic esophagitis, heart disease, and those requiring the use of beta-blockers or ACE inhibitors are relative contraindications for OIT. A decision to pursue OIT in these patients should be based on clinical judgment, provider expertise and shared decision-making	This recommendation is based on the principles of *nonmaleficence, equity of eligibility and patient autonomy* It is in line with the current general standard of care in allergen immunotherapy
Patient- or caregiver-specific contexts that may jeopardize the safe administration of therapy must be assessed. These include but are not limited to unreliable adherence to protocol, reluctance to use epinephrine, language barrier, severe anxiety, psychiatric barriers, non-collaborative family dynamics, lack of schedule flexibility for proper dosing, and lack of commitment from patient or caregivers. If these cannot be satisfactorily addressed, they constitute contraindications for OIT	This recommendation is based on the principle of *patient protection* It is supported by data from consultations with stakeholders

Recommendations for the safe provision of OIT *Summary of data synthesis (see details and references in* Table 1, *Clinical dimension, criteria 8 to 12): (see details and references in* Table 1, *Clinical dimension, criteria 8 to 12):* Clinical studies of OIT report that reactions requiring the use of epinephrine, including anaphylaxis, may occur in the clinic as well as during home dosing.

Eosinophilic esophagitis (EoE) is more prevalent in children with food allergy, especially those with milk or egg allergy, as compared to the general pediatric population, and may emerge during OIT. Certain studies report that EoE was managed with dose adjustments, though some patients discontinued OIT because of EoE.

**Additional key points from deliberation**

Some patients have gastro-intestinal symptoms that occur independently of the timing of food dosing. These can often be controlled by modifying the treatment schedule or by adding specific medication. Patients and families should be informed that endoscopy-directed biopsy may be required when symptoms compatible with EoE or eosinophilic gastro-intestinal disease (EGID) arise during OIT.

**Table:** 

Box 6: Recommendations for the safe provision of OIT	Ethical imperative, data or other considerations in support of the recommendation *Level of evidence (applicable when recommendations are based on outcomes data from clinical studies)*
OIT providers and patients should be prepared to recognize and treat allergic reactions, including anaphylaxis, during OIT. Food escalation should only be performed in a clinic with appropriate equipment and infrastructure* available to treat anaphylaxis A personalized action plan should be provided to patients to guide management of reactions occurring at home Providers should only offer OIT in age groups in which they have training or experience in treating anaphylaxis (*see Additional file 1: Appendix 5 for guidance on equipment and infrastructure requirements)	This recommendation is based on the principle of *nonmaleficence* It is supported by a *large amount of consistent clinical evidence* indicating that there is a risk of anaphylactic reactions during OIT [22–24]. A proportion of these reactions occur outside the healthcare setting [29, 32, 36, 38–40]. This is coherent with data from consultations. *Level of evidence: HIGH*
Patients should be observed in clinic for 1 h following dose escalation. The observation period can be decreased as appropriate to a minimum of 30 min, based on various factors that include patients who are reliable, confident and comfortable with the management of allergic reactions	This recommendation is based on the principle of *nonmaleficence* It is supported by data from consultations with stakeholders
Surveillance for the emergence of EoE or EGID should be based on monitoring for the emergence of clinical symptoms (e.g. dysphagia, oesophageal spasms, vomiting, diarrhea). Endoscopy and biopsy should be used to confirm the diagnosis in suspected cases not responding to dose adjustments or medication	This recommendation is based on the principle of *nonmaleficence* It is supported by data from consultations with stakeholders

Recommendations on personalized OIT protocols *Summary of data synthesis (see details and references in* Table 1, *Clinical dimension, criteria 8 to 12): (see details and references in* Table 1, *Clinical dimension, criteria 8 to 12):* The goals of OIT can be achieved with many different types of protocols, which vary in terms of dosing schedules, and food allergen product and preparation (Additional file 1: Appendix 3-Table OIT PROTOCOLS). Most OIT clinical trials and all OIT clinical practice studies used non-pharmaceutical food-based products. There is no evidence that pharmaceutical products offer any additional benefit.

A few studies directly compared different OIT protocols. One study indicated better efficacy and fewer moderate to severe reactions with more gradual up-dosing. Another study observed that patients were more likely to adhere with continued allergen consumption when the maintenance dose was lowered. The frequency of maintenance dosing had an impact on reactions and adherence in one study, while another study found no difference after 1 year of maintenance. When considering clinical factors that could be used to define treatment plan, the only prognostic factor consistently associated with treatment outcome was the baseline allergen-specific IgE level.

When considering multiple food OIT protocols, similar rates of reaction were observed in studies treating up to 5 food allergens simultaneously, as compared to single-food OIT. In addition, time to reach desensitization for all the foods with multiple-food OIT was only a few months longer than for one food with single-food OIT.

Studies have also looked at the use of omalizumab as part of an OIT protocol. A short course of omalizumab combined with an accelerated OIT schedule not only reduced the rate of dosing-related reactions, but helped improve desensitization rates. In contrast, extended use of omalizumab within a standard slow OIT schedule did not improve the final success rate.

**Additional key points from deliberation**

Decisions regarding when to initiate OIT, the initial food escalation, and the rate of escalation should be based on the same factors that help predict reactivity during food challenges and adapted to patient context. For example, it may be appropriate to proceed directly to OIT for a toddler with a recent reaction to relatively high amount of the culprit food whose food allergy has been confirmed with a skin test rather than to delay therapy while waiting for further confirmatory laboratory results. The objective is to respect the time patients and families have available, optimize the use of limited healthcare resources, and avoid missing a window of opportunity before a potentially unfavorable natural evolution of the disease.

In an ideal world, the rate of dosing escalation would be adjusted to match the patient’s change in reactivity threshold, which may not follow a linear trajectory. In practice, however, this is not always feasible for logistical reasons. In addition, fixed up-dosing schedules are also more easily implemented by OIT providers with less experience.

There is limited data on the advantage of a high maintenance dose over a lower dose when long term outcomes are assessed. Conceptually, the demonstrated efficacy of sub-lingual immunotherapy (SLIT) and epicutaneous immunotherapy (EPIT) suggests that a high dose may not be needed to modulate the immune response. Adherence with lower food doses is often easier, and a potentially more important goal than an unproven theoretical benefit a higher dose may have on sustained tolerance. Ultimately, however, determination of the target dose should include a consideration of the patient’s goals and preferences.

Treatment objectives can vary between patients as well as over time during therapy for a given patient. Some patients may desire the ability to stop treatment for a prolonged period (i.e., several weeks) without losing tolerance, while others should like to know that they can safely skip treatment for a few days, that they no longer need to be careful of certain co-factors or that they can safely ingest large amount of the food. Oral food challenges are necessary to assess many of these treatment objectives but consume time and resources. Therefore, monitoring plan should be personalized to specifically assess only the treatment outcomes that are relevant to a patient. Measurements of food-specific IgE and IgG4 are inexpensive and may also be helpful in the decision whether to proceed with an oral food challenge to test for these outcomes.

Assessing for sustained unresponsiveness requires a prolonged period of discontinuation of ingestion of the food and carries the risk of unnecessarily losing progress made with desensitization. Unless allergy testing has become negative, the preferred approach is to assess for sustained unresponsiveness is to progressively increase dosing intervals such that that dosing intervals can again be shortened if and when reactivity returns.

Because of its cost, recourse to omalizumab should occur responsibly and judiciously, as widespread unjustified use could jeopardize treatment sustainability. Despite its excellent safety profile, it is an injectable biologic drug, which can limit its acceptability for some patients.

**Table:** 

Box 7: Recommendations on personalized OIT protocols	Ethical imperative, data or other considerations in support of the recommendation *Level of evidence (applicable when recommendations are based on outcomes data from clinical studies)*
OIT can be performed with many different food products	This recommendation is based on the principles of *equity of access and sustainability*. Unless the superiority of a specific food product is demonstrated over other forms of the same allergen, choice of the product should be guided by availability, cost and practicality It is supported by data from consultations with stakeholders. In addition, despite a theoretical concern for the variability of non-standardized food products there is no evidence on the superiority of OIT protocols that use pharmaceutical products. (One meta-analysis of peanut OIT RCTs found that both proprietary and non-proprietary OIT products led to desensitization compared to placebo or usual care [24])
The goals of OIT can be achieved with many different protocols. There is little evidence that specific dosing schedules are superior to others. Reference protocols* can be useful to guide therapy but need to be selected and adapted based on the patient’s specific situation See Additional file 1: Appendix 6 for sample protocols	This general recommendation as well as the specific recommendations (A to D) that follow are supported by data from consultations with stakeholders and a large amount of successful published protocols that follow the same general approach (see OIT protocol variables in clinical studies or published clinical practice in Additional file 1: Appendix 3)
A. The initial dose that will be ingested at home should be determined during an initial dose escalation in clinic (day 1). This consists of a graded introduction of the allergen to identify the highest tolerated dose at baseline. The planned starting and ending doses for the initial escalation in clinic should be below the expected reactivity threshold and determined through shared decision making with patients and families. The objective is not necessarily to identify the reactivity threshold and induce a reaction as this can become a barrier to treatment. An alternative to multi-step escalation is to start treatment directly with a single dose assumed to be below the patient’s reactivity	These recommendations are based on the principle of *patient’s best interest* to limit the burden of unnecessary visits while preserving patient safety, as well as on the principle of *sustainability* and *best use of healthcare resources* The use of a flexible approach reflects the key elements of *personalized care* and contributes to *empowering patients* in the self-management of their food allergies. This is in line with earlier recommendations (see Box 1) to practice OIT in the spirit of personalized care and to promote patient empowerment
B. After initiating daily home ingestion of the tolerated dose, up-dosing increments should be adapted to patient evolution throughout therapy. Transient mild local reactions are to be expected in the first days following a dose increase. In the absence of any signs of reaction, the protocol could be accelerated. In the event of persistently recurring, moderate to severe or systemic reactions, dose progression must be decreased. The up-dosing intervals can be prolonged for medical or logistical reasons, or for personal preferences	
C. The final target dose for the therapy should be guided by the patient’s individual clinical response and personal goals, which can range from protection against accidental exposures to small amounts to unrestricted inclusion of the allergen into the diet. There is a lack of evidence that high maintenance doses will increase the likelihood of sustained unresponsiveness	This recommendation is based on the principle of *autonomy* to allow patients to achieve their individual goals according to their contexts.
D. During follow-up, persistence of desensitization is monitored by documenting a patient’s continued consumption of the food allergen. A decision to test for other outcomes should be guided by a patient’s personal objectives. Complete desensitization can be assessed by performing a high threshold challenge to the food. The risk of a dosing reactions associated with cofactors can be assessed by performing a food challenge in the presence of cofactors (e.g. alcohol and non-steroidal anti-inflammatory drugs, exercise). Sustained unresponsiveness can be assessed by the progressive increase in dosing intervals	This recommendation is based on the *adequacy of the intervention* in addressing the actual needs of patients
When performing OIT in patients with multiple food allergies, the preferred approach is to treat multiple foods simultaneously	This recommendation is based on the principle of *sustainability* and on practical consideration for patients It is supported by a *small amount of consistent clinical evidence*: one small non-randomized study observing similar safety outcomes between multiple-food OIT (targeting up to 5 foods simultaneously) as compared to single-food OIT and a small difference in treatment duration [68]. Additional small studies lend further support to suggesting the feasibility of multi-food OIT with [69, 71] or without [70] the concomitant use of omalizumab. This is coherent with data from consultations. *Level of evidence: LOW*
Short-term concomitant use of omalizumab can be considered in challenging cases	This recommendation is based on the principles of *nonmaleficence* and *equity of access* in the specific context of challenging cases. Limiting recourse of omalizumab to such challenging cases is based on the principle of *sustainability* It is supported by a *moderate amount of consistent clinical evidence* including two small placebo-controlled RCTs rated as being at low-risk of bias, in which, compared to placebo, the concomitant use of omalizumab allowed for more rapid up-dosing with similar or fewer adverse reactions [71, 73]. This is coherent with data from consultations. *Level of evidence: MODERATE*

Recommendations for patient-centered care *Summary of data synthesis (see details and references in* Table 1, *Clinical dimension, criteria 8 to 12):* One of the most important goals patients have regarding the management of their food allergy is to reduce the anxiety and achieve a sense of control by reducing the risk associated with accidental exposure. This can be achieved in different ways, including through OIT.

OIT involves ingesting an allergen that was previously avoided, which can initially be very challenging and an understandable source of anxiety. Reactions can occur with food dosing, and as is the case in all food reactions, including those due to unintentional exposures, both patients and clinicians find it difficult to distinguish between allergic symptoms and symptoms due to anxiety.

OIT is time-consuming for multiple reasons, including the number of clinic visits necessary for up-dosing, and a period of reduced activity that is required before and after food dosing to reduce the risk of reactions. A patient can be bothered by the amount and taste of food they must consume for OIT. Nevertheless, published studies and consultation data suggest that most patients do not consider OIT an overwhelming burden.

Misconceptions regarding risks and outcomes of OIT have the potential to create unrealistic expectations or concerns. Interestingly, one clinical study showed that anxiety was reduced and adherence with OIT improved when the occurrence of mild reactions was presented in a positive light as being part of the process and indicative of progress towards treatment goals.

The majority of studies on OIT show that patients receiving OIT report improved food allergy-related quality of life, particularly with respect to anxiety. A subset of patients can experience a deterioration in quality of life measures at the beginning of treatment, but this tends to resolve upon reaching maintenance. The greatest improvements in quality of life are reported by patients for whom food allergy had the strongest impact on quality of life before starting treatment.

**Additional key points from deliberation**

Shared decision-making is a key component of patient-centered food allergy management. Providers must communicate effectively to explain all treatment options, including continued avoidance and food OIT, so that patients are able to make a choice that aligns with their preferences, values and clinical needs. Communication between patients and their provider before initiating OIT and throughout treatment is of the utmost importance to ensure that patient and provider treatment goals are aligned, expectations remain realistic, and anxiety is well managed.

During the maintenance phase of OIT, many patients want to be informed about their treatment progress beyond their level of desensitization to the daily doses. This includes information about whether they also have tolerance to food amounts above the treatment dose, the actual risk associated with skipping doses and the relevance of various cofactors in allergic reactions to foods. The observation of longitudinal changes in the underlying immune response is highly relevant to patients and is used in some clinical practices to guide decisions. Monitoring food-specific IgE and IgG4 levels may be useful in that regard; however, more research is needed to clarify the real-life interpretation and relevance of these within the context of OIT.

Research on the impact of other interventions, such as food allergy education, on quality of life would contribute to inform best approaches and optimise care and resource use.

**Table:** 

Box 8: Recommendations for patient-centered care	Ethical imperative, data or other considerations in support of the recommendation
The ultimate goal of food allergy care should be the empowerment of patients and their caregivers to manage the risk of food allergy reactions, reduce food-related anxiety and achieve a sense of control over their condition. This can be achieved in different ways for different patients. Tactful and empathic shared decision making with patients, their caregivers and the OIT provider, is necessary before making a decision to proceed with OIT	This recommendation is based on the importance of understanding patients’ perspective, with thoughtful consideration for the needs and values of each individual, which is the essence of *empathy*, as well as on the importance of promoting patient empowerment, which reflects the principle of *autonomy* It is also based on the *adequacy* of the intervention in addressing the actual needs of patients It is supported by data from consultations with stakeholders and key aspects emerging from the literature
Informed consent must be obtained before initiating OIT. This should include clear discussion of potential outcomes, risks and benefits, as well as of patients’ and their caregivers’ concerns, expectations and goals. Patients should be informed on how to recognize and manage reactions during therapy Throughout treatment, patients’ goals and perceived benefits should be reassessed periodically to ensure that clinical decisions continue to reflect their personal objectives. When appropriate, expected mild reactions should be framed in a positive manner that reduces perceived burden of therapy and promotes a sense of control	This recommendation is based on the principle of *decisional autonomy* It is supported by data from consultation with stakeholders as well as a *small amount of clinical evidence* (one clinical trial on psychologic intervention in support of OIT [74]). *Level of evidence: LOW*

### Organizational dimension: promotion optimized organization of care

Recommendations for the promotion of optimized organization of care *Summary of data synthesis (see details and references in* Table 1, *Organizational dimension, criteria 13 and 14):* OIT is a viable therapeutic option and so falls within the mandate of the healthcare system. Healthcare resources for food allergy are currently based on an approach to management focused on the complete avoidance of the offending allergen; thus, the implementation of OIT will require a complete reorganization of food allergy care, while also maintaining support for patients who continue with avoidance alone.

All physicians involved in administering OIT must be able to recognize and manage different types of adverse food reactions and must have access to equipment required to treat severe reactions. A multidisciplinary approach to OIT can improve patient outcomes and optimize the use of resources by including other relevant healthcare personnel who can assist the physician, including nurses, registered dieticians and psychologists.

**Additional key elements from deliberation**

A multidisciplinary approach could contribute to improved quality of care and a sustainable wide scale offer of OIT by relieving OIT providers of certain tasks that other healthcare professionals can perform. Nurses can be essential as a point of contact for patients and for coordinating care. Registered dieticians can provide unique support in identifying equivalent food alternatives for home dosing and to monitor nutritional needs. Psychologists can offer support within the context of individual or group interactions. Group interactions have the unique potential to offer professional support within the context of sharing issues with others, supporting others with similar concerns and questions, and collaboratively identifying solutions. Patient groups can also provide an outlet to discuss issues that might not otherwise arise if the patient interacts with healthcare professionals only. Integrating peer supporters into the clinical team can help ensure the alignment of key messages.

Regarding access disparities, offering training and support to pediatricians and family doctors who wish to offer OIT under the direct supervision of a subspecialist allergist could greatly contribute to make the treatment accessible to a much larger number of patients in rural areas, where few or no allergists are available, or in urban areas, where there are not enough allergists to respond to patient demand. Conditions for the safe provision of OIT (see Box 6) need to be always maintained, including equipment and infrastructure requirements.

The majority of clinical and research OIT initiatives have focused on children to this point which creates a risk of a knowledge deficit in the adult allergy/immunology training curriculum that could impact future capacity to offer treatment in this patient group.

**Table:** 

Box 9: Recommendations for the promotion of optimized organization of care	Ethical imperative, data or other considerations in support of the recommendation
To optimize human resources and ensure optimal delivery of quality care in food allergy, a multidisciplinary approach adapted to patient needs should be promoted, and should include nurses, registered dieticians, psychologists and peer supporters, when possible	This recommendation stems from *responsibility* of the healthcare system to deliver the best possible care to all patients while optimizing healthcare resources It is supported by data from consultations with stakeholders
In areas with limited or no access to allergists, pediatricians and family physicians could provide certain OIT services, after receiving adequate training and under close supervision by an allergist	This recommendation is based on the principle of *equity of access* by adapting available resources to local contexts, and thus minimizing regional disparities in OIT provision It is supported by data from consultations with stakeholders

### Economic dimension: promotion of sustainability

Recommendations for sustainable and responsible provision of OIT *Summary of data synthesis (see details and references in* Table 1, *Economic dimension, criteria 15 to 21):* The price of a commercial product developed for peanut OIT is based on the cost of pharmaceutical development, which makes it approximately 100 times higher in cost a similar non-pharmaceutical-based approach produced within the healthcare system. When using non-pharmaceutical products, the cost of OIT treatment derives primarily from healthcare practitioner’s services, whereas when using pharmaceutical product, the commercial food OIT product represents a significant additional and long-term expense without reducing base costs. With the commercially developed peanut OIT product, the economic model assumes lifelong treatment based on the manufacturer’s stated indication, while a non-pharmaceutical-based approach assumes that patient can use regular food. This raises ethical issues regarding the best interests of a patient, which is to have an open diet, and the best interest of the healthcare system, which is to ensure sustainability over the long term.

On the patient side, data shows that avoidance is associated with significant costs to patients, because as one patient said, “fear makes us spend”. OIT, in the short term, will increase cost associated with physician visits (including time off work), which will be reduced and possibly lower than avoidance once on maintenance. From the societal side, OIT is perceived as a good investment which could prevent future healthcare and productivity issues, especially when performed early.

**Additional key points from deliberation**

A pharmaceutical-based-approach to OIT carries the risk of diverting limited resources in food allergy care to cover high cost products and away from strengthening treatment capacity within the healthcare system.

The notion of “off-label” use does not apply to food—characterizing the practice of OIT with common food as “off-label” in reference to an alternative commercial product is in neither the best interest of patients nor of the healthcare system. To this point, foods for other medical procedures, including diagnostic food challenges, are currently bought at the grocery store and measured in clinic. The cost of a prepackaged standardized food dose should reflect its true value in clinic, which is ultimately of a practical nature, rather than the fact that it has been categorized as a drug.

**Table:** 

Box 10: Recommendations for sustainable provision of OIT	Ethical imperative, data or other considerations in support of the recommendation
In the development of OIT, extreme care should be taken to avoid creating unnecessary financial barriers that could limit access to treatment based on ability to pay	This recommendation is based on the core value of public healthcare systems, which aim at *reducing social inequalities in health* It is supported by data from consultations with stakeholders
Investment by the healthcare system in food allergy treatment should be guided by patients’ best interests and system sustainability. As such, investments should be encouraged toward measures that contribute to building the capacity for OIT within the system (e.g. multidisciplinary teams, preparation of personalized food products, treatment monitoring) in order to achieve meaningful and cost-effective impacts on patient outcomes	This recommendation stems from the principles of *sustainability* and a *fair and efficient allocation* of healthcare resources It is supported by data from consultations with stakeholders and data from milieu of care

## Discussion

This development process of these CPGs consisted of a comprehensive approach to both the data collection and the collection of perspectives from all stakeholders. This allowed for the collaborative development of 38 recommendations guided by the five principles of the common good, equity, health and wellbeing, optimized organization of care and sustainability of healthcare systems, allowing a 360° view of OIT.

This comprehensive approach included the full extent of a traditional review of the available clinical data. The large number of both RCTs and diverse observational studies provided a rich body of complementary evidence. In addition, the inclusion of patients in both the consultation and the deliberation phases provided key experiential knowledge and contributed to the interpretation of data and the development of recommendations. Similar involvement of first line and allied healthcare professionals ensured a multidisciplinary perspective for optimal organization and provision of care as well as efficient use of healthcare resources. Throughout the aforementioned steps, the multicriteria methodology drove the systematic collection of scientific and experiential knowledge for all dimensions, the sociopolitical, populational, clinical, organizational and economic. This was instrumental in developing balanced recommendations allowing us to address issues beyond what is usually covered by traditional CPGs.

This was critical in the specific context of OIT, which poses challenges beyond its clinical aspects. Offering OIT requires adapting the organization of care in a setting of highly limited resources. It marks a rupture with a culture focusing on avoiding reactions at all cost, which creates confusion and tensions. Competing developments as a drug-based versus a food-based therapy also create a number of ethical issues. The methodology provides an ethics-based framework to identify and address these issues in the elaboration of recommendations.

The 360° approach necessitates a team with diverse expertise in various methodological domains and fields of knowledge to collect and integrate the body of knowledge in the multicriteria grid. Expertise in communicating in lay language and chairing a discussion on all these aspects is essential to ensure an understanding by all. The inclusion of participants with diverging perspectives is important to stimulate individual and group reflection during discussions. A tailored multidisciplinary team and a method that allows for efficient organization of data per criteria are essential to the process and key to promote understanding of numerous complex concepts and fulfill the A4R conditions.

Accountability for Reasonableness (A4R) defines four main conditions that can enhance legitimacy and help stakeholders develop a mutual basis for decision making: publicity of rationales for choices; relevance of criteria agreed to by a broad range of stakeholders; revisability of the decision in light of new evidence or arguments; and enforcement that means the other conditions are met [14]. The multicriteria approach facilitates meting these four conditions. The use and publication of the multicriteria grid completed with the data, from which detailed and lay rationales were developed, addresses the publicity and the relevance conditions. The multicriteria grid also offers a pragmatic tool to update the CPGs as significant advancements in knowledge emerges, which contributes to revisability. The implication of a large number of stakeholders, of methodologists and ethicists, the participation of external observers at the deliberation, as well as oversight by the CSACI board contributed to the enforcement of these three conditions.

Clinical recommendations developed here are generally concordant with those from the European Academy of Allergy and Clinical Immunology (EAACI) [8]. That said, in addition to the recommendations made outside the clinical dimension, there are some noteworthy differences between the CPGs, which likely stem from the difference in methodology. The authors of the EAACI guidelines explicitly stated in their discussion that “there is no evidence that the efficacy and safety are affected by the type and nature of the food allergen used in allergen immunotherapy”. However, their methodology resulted in limiting the indication for OIT to milk, egg and peanut only. The current guidelines diverge from those from EEACI in a few other recommendations, namely regarding the indication for OIT in children less than 4 years of age or in adults and the recognition that OIT can promote sustained unresponsiveness in some patients, over the long term.

There was also a difference in the overall vision surrounding the development of OIT. In the EAACI guidelines, the need for standardized protocols and products was identified as a high priority, while these CPGs emphasize that there is a need for more personalization. Standardization is often seen as a desirable objective in modern medicine because it allows generating quantitative data that can be used as the basis for more recommendations using a traditional CPG methodology. However, the ability to adapt protocols to patients’ needs is essential in OIT. Standardized products and protocols have not been shown to provide clinical benefits over their adaptable alternatives and can actually increase barriers and costs, thus threatening sustainability and access.

Personalized approaches, however, represent a challenge for the transfer of expertise. The development of training initiatives, clinical tools, and organizational models adapted to the delivery of personalized care should therefore be made a priority. Shared decision-making tools should include clear information on food allergy management, consent forms, home dosing instructions, and a reaction management plan. Specific training tools directed toward allergists, primary care physicians and allied healthcare professionals should focus on the biological, psychological and social aspects of the therapy and be included in training curricula and continuing professional development (CPD).

Clinical tools will be developed collaboratively as a second step of these CPGs and made available online at http://www.csaci.ca/OIT-guidelines.

These CPGs also identified a number of data gaps that require further research, including:

- long-term patient-centered outcomes
- underrepresented groups, e.g. adults
- optimal use of biomarkers to guide therapy
- optimal use of medication to support treatment
- determinants of reactivity, e.g. cofactors, individual differences in absorption and distribution of allergens
- benefits and optimal use of nutritional, psychological, and peer support
- economic and organizational aspects of the treatment.

## Conclusion

**Fig. 4:**
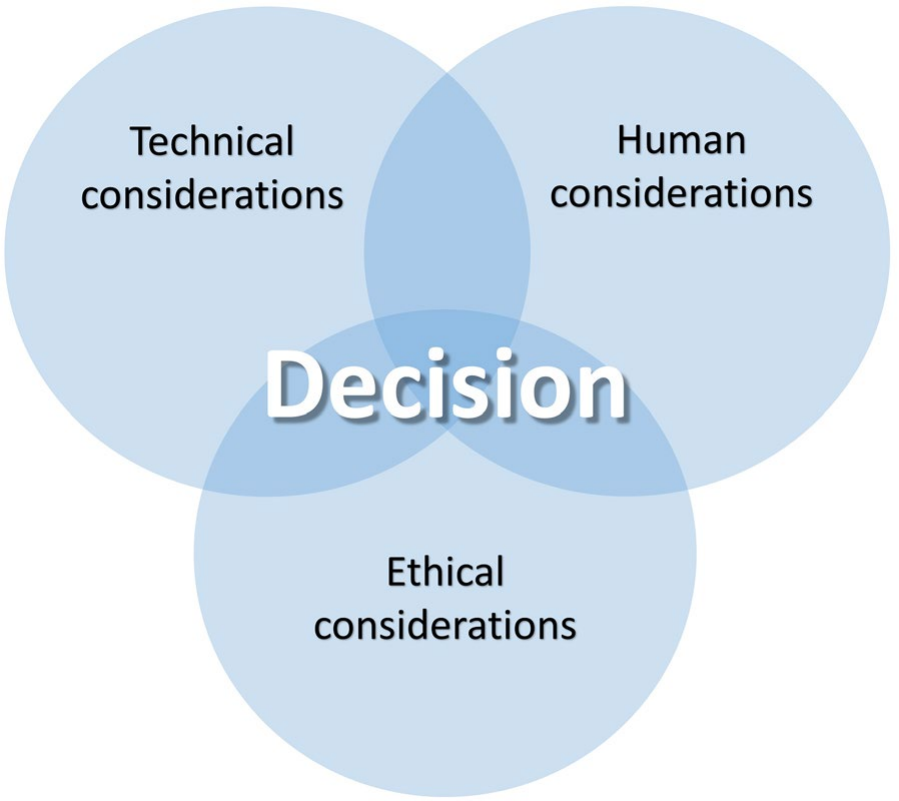
Considerations for balanced decision-making in medicine

These guidelines bring to the forefront the critical importance and value of placing patients at the center of the development of clinical practice guidelines. Here, this approach was instrumental to developing recommendations for the responsible implementation of OIT in clinical practice, adapted to individual patient needs. The multicriteria approach offers an alternative to technocentric approaches in CPG development by balancing human, ethical and technical considerations in decision making (Fig. 4).

Technocentric approaches tend to create a pressure to standardize patient care in order to generate quantitative data, which may not always be in patients’ best interest. Rather than adapting patient care to meet methodological needs for quantitative data, the methodology should be adapted to patients’ needs for best care. As the saying goes, ‘Not everything that counts can be counted, and not everything that can be counted counts [75].’

## Supplementary information

**Additional file 1.** Supplementary appendices.

## Data Availability

Additional data is included in the supplemental repository. Clinical tools for implementation in practice will be made available at http://www.csaci.ca/OIT.

## Abbreviations

A4R: Accountability for reasonableness
CCT: Controlled clinical trial
CPG: Clinical practice guidelines
CSACI: Canadian Society of Allergy and Clinical Immunology
EAACI: European Academy of Allergy and Clinical Immunology
EoE: Eosinophilic esophagitis
EPIT: Epicutaneous Immunotherapy
IHE: Institute for Health Economics
OIT: Oral immunotherapy
RCT: Randomized controlled clinical trial
SLIT: Sub-lingual Immunotherapy
