# Supplementary material for: CSACI guidelines for the ethical, evidence-based and patient-oriented clinical practice of oral immunotherapy in IgE-mediated food allergy
Source: Allergy Asthma Clin Immunol. 2020 Mar 18;16:20. doi: 10.1186/s13223-020-0413-7 (PMC7079444; doi:10.1186/s13223-020-0413-7)
Supplement: Supplementary file 1 — Additional file 1. Supplementary appendices. [file 13223_2020_413_MOESM1_ESM.docx]

Contents

[Appendix 1: Management of conflict of interests and roles 3](#_Toc27666827)

[Appendix 2: Literature review details 8](#_Toc27666828)

[Appendix 2A: Bibliographical database search strategy and terms 8](#_Toc27666829)

[First search: Outcomes of oral immunotherapy 8](#_Toc27666830)

[Second search: Epidemiology, burden of illness, impact on quality of life 11](#_Toc27666831)

[Appendix 2B: Eligibility criteria for inclusion of clinical studies 17](#_Toc27666832)

[Appendix 3: Detailed clinical evidence tables 19](#_Toc27666833)

[OIT PROTOCOL VARIABLES IN CLINICAL STUDIES OR PUBLISHED CLINICAL PRACTICE 19](#_Toc27666834)

[CLINICAL EFFICACY 23](#_Toc27666835)

[Desensitization: increase in reactivity threshold 23](#_Toc27666836)

[Data for peanut, milk and egg allergies 23](#_Toc27666837)

[Data for other food allergies 26](#_Toc27666838)

[Assessment of the quality of evidence 27](#_Toc27666839)

[Continued allergen food consumption 28](#_Toc27666840)

[Data for peanut, milk and egg allergies 28](#_Toc27666841)

[Data for other food allergies 30](#_Toc27666842)

[Assessment of the quality of evidence 31](#_Toc27666843)

[Sustained unresponsiveness 32](#_Toc27666844)

[Data for peanut, milk, egg allergies 32](#_Toc27666845)

[Allergens other than milk, egg and peanut 34](#_Toc27666846)

[Assessment of the quality of evidence 35](#_Toc27666847)

[Impact of patient characteristics on efficacy outcomes 35](#_Toc27666848)

[Correlations between baseline parameters and OIT efficacy outcomes 38](#_Toc27666849)

[Correlations between baseline asthma status and OIT outcomes 40](#_Toc27666850)

[Correlations between baseline multiple food allergies single-food OIT outcomes in large case series 41](#_Toc27666851)

[CLINICAL SAFETY 42](#_Toc27666852)

[Severe reactions /Systemic reactions/ anaphylaxis / serious adverse events / epinephrine use 42](#_Toc27666853)

[Data for peanut, milk, egg allergies 42](#_Toc27666854)

[Allergens other than milk, egg and peanut 44](#_Toc27666855)

[Assessment of the quality of evidence 46](#_Toc27666856)

[Any adverse reactions / any allergic reactions / local reactions 47](#_Toc27666857)

[Data for peanut, milk and egg allergy 47](#_Toc27666858)

[Allergens other than milk, egg and peanut 48](#_Toc27666859)

[Assessment of the quality of evidence 49](#_Toc27666860)

[Discontinuations due to adverse events 50](#_Toc27666861)

[Data for peanut, milk and egg allergy 50](#_Toc27666862)

[Allergens other than milk, egg and peanut 51](#_Toc27666863)

[Assessment of the quality of evidence 52](#_Toc27666864)

[Long-term FU for safety and tolerability 54](#_Toc27666865)

[ASSESSMENT OF THE QUALITY OF EVIDENCE 56](#_Toc27666866)

[Eosinophilic esophagitis 57](#_Toc27666867)

[Impact of patient characteristics on safety outcomes 59](#_Toc27666868)

[Impact of baseline food allergy-related parameters on safety outcomes 60](#_Toc27666869)

[Impact of baseline asthma status on safety outcomes 63](#_Toc27666870)

[IMPACT ON QUALITY OF LIFE 64](#_Toc27666871)

[References 69](#_Toc27666872)

[Appendix 4: Narrative summaries of the data that led to the recommendations 76](#_Toc27666873)

[Recommendations on patient-centered care 76](#_Toc27666874)

[Recommendations on eligible food allergens and clinical outcomes that can be achieved by OIT 76](#_Toc27666875)

[Recommendations on who could benefit from OIT (indications) 77](#_Toc27666876)

[Recommendations on contra-indications 77](#_Toc27666877)

[Recommendations on personalized protocols 78](#_Toc27666878)

[Recommendations for the safe provision of OIT 78](#_Toc27666879)

[Appendix 5: Equipment required 79](#_Toc27666880)

[Appendix 6: Sample protocols 80](#_Toc27666881)

[Equivalency table: protein content per type of allergen 81](#_Toc27666882)

[Sample dosing schedules 82](#_Toc27666883)

[CoFAR schedule 82](#_Toc27666884)

[Omalizumab-enabled accelerated schedule 83](#_Toc27666885)

[Pre-school peanut schedule 83](#_Toc27666886)

[Individualized symptom-driven up-dosing 84](#_Toc27666887)

[Pre-medication 85](#_Toc27666888)

# Appendix 1: Management of conflict of interests and roles

**Orientations for the implementation of Guidelines International Network (GIN) principles - OIT Guideline development**

|  | **(GIN) (2015) Principles** | **Proposed orientations for the implementation of GIN principles - OIT Guideline development** |
| --- | --- | --- |
| **Defining and declaring COIs** | *Principle 2: The definition of COI and its management applies to all members of a guideline development group, regardless of the discipline or stakeholders they represent, and this should be determined before a panel is constituted.* | 1. Definitions: **Conflict of interests (COI):** situations in which the professional judgement of an individual involved in developing the CPG can be unduly influenced by an opportunity to derive personal benefit. A **specific interest** is related to the topic under evaluation. 2. The declarant must declare his/her own interests as well as the interests of spouses living in the same residence as the declarant, children, parents (father and mother) and siblings. Interests from friends, other family members, and business partners must also be declared, if they are known to the declarant and could affect impartiality and independence of the declarant’s judgment. Interests of organizations in which the declarant holds a managerial position must also be declared. 3. The principles and orientations proposed apply to every person working on the OIT Guideline development, including the members of: the Clinical Practice Guideline Development Executive Team; the CSACI Oral Immunotherapy CPG Working Group; the Scientific Support team; the Deliberative Committee; and the individuals (experts/advisors/patients) consulted as part of the consultations activities. |
|  | *Principle 3: A guideline development group should use standardized forms for disclosure of interests* | 1. The declaration is made via a declaration of interests form. It must be renewed at least every year. Any change which places the declarant in a situation conducive to the development of conflicts of interest or roles must be declared as soon as possible. 2. The types of interests that are to be declared include:    1. Financial or non-financial interests or roles, specific or non-specific to the object of the work;    2. Personal, professional or institutional activities, past, present or future (already planned), actual, apparent or potential. 3. Interests held in the 36 months preceding the declaration, and those foreseeable, are to be declared. |
|  | *Principle 4: A guideline development group should disclose all relevant interests publicly, and these should be easily accessible for users of the guideline.* | 1. The COIs of every person working on the OIT Guideline development, the reasons for including conflicted members and the management of COIs, will be disclosed explicitly and publicly, i.e. they will be published and described with the Guideline. 2. The funding source of the Guideline will be disclosed and published along with the Guideline. The funding for the OIT Guideline development comes from the membership to the Canadian Society of Allergy and Clinical Immunology. No industry money is used to fund the activities. 3. The role of the sponsor(s) and the support provided for the development of the guideline will be described within the Guideline. |
|  | *Principle 5: All members of a guideline development group should declare and update any changes in interests at each meeting of the group and at regular intervals (for example, annually for standing guideline development groups).* | 1. The declaration of interests must be renewed every year. Any change which places the declarant in a situation conducive to the development of conflicts of interest must be declared at the earliest opportunity, for example, orally at the start of meetings and/or by updating the original form. |
| **Oversight Committee** | *Principle 9: An oversight committee should be responsible for developing and implementing rules related to COIs.* | 1. The CSACI Guidelines Selection Committee, designated by the CSACI executive, reviews the written declarations of COIs from CSACI members who volunteered to be part of the CSACI OIT CPG Working Group. 2. The Committee reviews written COIs declarations from the Clinical Practice Guideline Development Executive Team, the CSACI OIT CPG Working Group, the Scientific Support team, the Deliberative Committee and the individuals (experts/advisors/patients) consulted. 3. The Committee evaluates the interests declared applying the following criteria, using an evaluation grid (to be developed):   1) interest or role (nature, value, nature of the organization involved, specificity, seniority, duration, repetition);  2) the declarant (holder of the interest, nature of the link with an outside organization);  3) the mandate of the declarant;  4) the potential consequences of the conflict of interests or roles.   1. The Committee determines one of three potential responses to a conflict of interest:    1. No action other than open disclosure    2. Partial exclusion (e.g., include only for consultation but not for deliberation, i.e., decision-making, or if included in deliberation, exclude from voting on specific recommendations)    3. Complete exclusion 2. The Committee retains the declaration forms five years after the publication of the guidelines 3. The Committee takes action if a conflict of interest was not identified, declared or managed appropriately. |
| **GDG composition** | *Principle 1: Guideline developers should make all possible efforts to not include members with direct financial or relevant indirect COIs.* | 1. Members of the CSACI Oral Immunotherapy CPG Working Group, the CPG Development Executive Team, the Scientific support team, the Deliberative Committee, and all individuals consulted must submit a disclosure of interests form. 2. More than one third of persons participating at the OIT Guideline Development should not have COIs (financial or non-financial; specific or non-specific to the object of the work; personal, professional or institutional - including, but not limited to: patents; stocks; equity positions; gifts; consulting agreements, employment arrangements, research grants/ contracts, and travel reimbursement and honorariums sponsored by industry; representation of an association whose purpose, mission or interests that are related to the objects of evaluation; advocacy). The inclusion of conflicted persons should be explicitly justified (e.g. irreplaceable expertise, geographical representation). |
|  | *Principle 8: No member of the guideline development group deciding about the direction or strength of a recommendation should have a direct financial COI.* | 1. When a member of the deliberative Committee has a financial COI directly related to a specific recommendation, he/she will be excluded from the discussion (physically absent) on this particular recommendation.   Financial COIs include, but are not limited to, patents, stocks, equity positions, gifts, consulting agreements, employment arrangements, research grants/ contracts, and travel reimbursement and honorariums sponsored by industry as well as interests that relate to the development, manufacture or marketing of products that may be considered by the committee.   1. Individuals who are likely to be excluded from more than half of the Deliberative Committee’s discussions because of financial COIs should not be appointed as members of the Deliberative committee. 2. More than half of the Deliberative Committee members who deliberate on a specific recommendation should not have any COIs related to this recommendation (financial or non-financial; specific or non-specific to the objects of the work; personal, professional or institutional - including, but not limited to: patents; stocks; equity positions; gifts; consulting agreements, employment arrangements, research grants/ contracts, and travel reimbursement and honorariums sponsored by industry; representation of an association whose purpose, mission or interests are related to the objects of evaluation ; public comments, testimony, and previously published opinions including policy positions; leadership role on a panel; substantial career efforts/ interests relating to the services, interventions, products, or delivery of care to be considered within the scope of the guideline; advocacy). The inclusion of conflicted participants should be justified (e.g. irreplaceable expertise). |
|  | *Principle 6: Chairs of guideline development groups should have no direct financial or relevant indirect COIs.*  *When direct or indirect COIs of a chair are unavoidable, a co-chair with no COIs who leads the guideline panel should be appointed.* | 1. The Chair and the co-chair of the Deliberative Committee should have no COIs:  - no direct financial interests including, but not limited to: patents; stocks; equity positions; gifts; consulting agreements, employment arrangements, research grants/ contracts, and travel reimbursement and honorariums sponsored by industry; interests that relates to the development, manufacture or marketing of products that may be consider by the committee. - no other relevant interests including, but not limited to: representation of an association whose purpose, mission or interests are related to the objects of evaluation; public comments, testimony, and previously published opinions including policy positions; leadership role on a panel; substantial career efforts/ interests relating to the services, interventions, products, or delivery of care to be considered within the scope of the guideline; advocacy. |
|  | *Principle 7: Experts with relevant COIs and specific knowledge or expertise may be permitted to participate in discussion of individual topics, but there should be an appropriate balance of opinion among those sought to provide input.* | 1. All individuals consulted (experts/advisers/patients) must declare their interests. 2. Individuals with relevant COIs and specific knowledge or expertise may be consulted and participate in the discussion of the Deliberative Committee. 3. If consulted individuals with COIs are invited to a meeting of the Deliberative Committee, they should declare their interests at each meeting they attend before giving their testimony. 4. The consultation process seeks to capture a diversity of opinions. |

# Appendix 2: Literature review details

## Appendix 2A: Bibliographical database search strategy and terms

### First search: Outcomes of oral immunotherapy

PubMed 17 April 2019

| #1 | Food Hypersensitivity[MH] |
| --- | --- |
| #2 | Hypersensitivity[MH] OR allerg*[TIAB] OR hypersensitiv*[TIAB] OR hyper-sensitiv*[TIAB] OR hyperresponsiv*[TIAB] OR hyper-responsiv*[TIAB] OR allerg*[OT] OR hypersensitiv*[OT] OR hyper-sensitiv*[OT] OR hyperresponsiv*[OT] OR hyper-responsiv*[OT] |
| #3 | Food[Mesh] OR additive[TIAB] OR additives[TIAB] OR almond*[TIAB] OR apple[TIAB] OR apples[TIAB] OR apricot*[TIAB] OR arachis hypogaea[TIAB] OR avocado*[TIAB] OR banana*[TIAB] OR barley[TIAB] OR beef[TIAB] OR berries[TIAB] OR berry[TIAB] OR blackberr*[TIAB] OR blueberr*[TIAB] OR buckwheat*[TIAB] OR cacao[TIAB] OR calamari[TIAB] OR carrot*[TIAB] OR cashew*[TIAB] OR celery[TIAB] OR cherries[TIAB] OR cherry[TIAB] OR chestnut*[TIAB] OR chicory[TIAB] OR chocolat*[TIAB] OR cilandro[TIAB] OR clam[TIAB] OR clams[TIAB] OR cockle*[TIAB] OR coconut[TIAB] OR coconuts[TIAB] OR conch[TIAB] OR conchs[TIAB] OR coriander[TIAB] OR corn[TIAB] OR crab[TIAB] OR crabs[TIAB] OR cranberr*[TIAB] OR crawfish*[TIAB] OR crayfish*[TIAB] OR crustacea*[TIAB] OR cucumber*[TIAB] OR dairy[TIAB] OR dairies[TIAB] OR edamame*[TIAB] OR egg[TIAB] OR eggplant*[TIAB] OR eggs[TIAB] OR fig[TIAB] OR figs[TIAB] OR fish[TIAB] OR fishes[TIAB] OR flaxseed*[TIAB] OR food[TIAB] OR foods[TIAB] OR fruit[TIAB] OR fruits[TIAB] OR grape[TIAB] OR grapes[TIAB] OR hazelnut*[TIAB] OR kiwi[TIAB] OR kiwis[TIAB] OR lactose[TIAB] OR legume*[TIAB] OR lobster[TIAB] OR lobsters[TIAB] OR mango*[TIAB] OR meat[TIAB] OR melon[TIAB] OR melons[TIAB] OR milk[TIAB] OR mollusc*[TIAB] OR multifood[TIAB] OR mushroom*[TIAB] OR mussel[TIAB] OR mussels[TIAB] OR mustard[TIAB] OR nut[TIAB] OR nuts[TIAB] OR oat[TIAB] OR oats[TIAB] OR octopus[TIAB] OR "oral allergy syndrome"[TIAB] OR orange*[TIAB] OR oyster[TIAB] OR oysters[TIAB] OR parsley[TIAB] OR pea[TIAB] OR peach*[TIAB] OR peanut*[TIAB] OR pear[TIAB] OR pears[TIAB] OR peas[TIAB] OR pecan*[TIAB] OR periwinkle*[TIAB] OR pineapple*[TIAB] OR pistachio*[TIAB] OR plum[TIAB] OR plums[TIAB] OR pork[TIAB] OR potato*[TIAB] OR prawn[TIAB] OR prawns[TIAB] OR raspberr*[TIAB] OR rice[TIAB] OR rye[TIAB] OR salmon[TIAB] OR scallop[TIAB] OR scallops[TIAB] OR seafood*[TIAB] OR sesame[TIAB] OR shellfish*[TIAB] OR shrimp[TIAB] OR shrimps[TIAB] OR snail[TIAB] OR snails[TIAB] OR soja[TIAB] OR sojabean*[TIAB] OR soy[TIAB] OR soya[TIAB] OR soyabean*[TIAB] OR soybean*[TIAB] OR squid[TIAB] OR strawberr*[TIAB] OR sunflower seed*[TIAB] OR tomato*[TIAB] OR vegetable*[TIAB] OR walnut*[TIAB] OR wheat[TIAB] OR wheats[TIAB] OR whelk[TIAB] OR whelks[TIAB] OR zucchini*[TIAB] OR additive[OT] OR additives[OT] OR almond*[OT] OR apple[OT] OR apples[OT] OR apricot*[OT] OR arachis hypogaea[OT] OR avocado*[OT] OR banana*[OT] OR barley[OT] OR beef[OT] OR berries[OT] OR berry[OT] OR blackberr*[OT] OR blueberr*[OT] OR buckwheat*[OT] OR cacao[OT] OR calamari[OT] OR carrot*[OT] OR cashew*[OT] OR celery[OT] OR cherries[OT] OR cherry[OT] OR chestnut*[OT] OR chicory[OT] OR chocolat*[OT] OR cilandro[OT] OR clam[OT] OR clams[OT] OR cockle*[OT] OR coconut[OT] OR coconuts[OT] OR conch[OT] OR conchs[OT] OR coriander[OT] OR corn[OT] OR crab[OT] OR crabs[OT] OR cranberr*[OT] OR crawfish*[OT] OR crayfish*[OT] OR crustacea*[OT] OR cucumber*[OT] OR dairy[OT] OR dairies[OT] OR edamame*[OT] OR egg[OT] OR eggplant*[OT] OR eggs[OT] OR fig[OT] OR figs[OT] OR fish[OT] OR fishes[OT] OR flaxseed*[OT] OR food[OT] OR foods[OT] OR fruit[OT] OR fruits[OT] OR grape[OT] OR grapes[OT] OR hazelnut*[OT] OR kiwi[OT] OR kiwis[OT] OR lactose[OT] OR legume*[OT] OR lobster[OT] OR lobsters[OT] OR mango*[OT] OR meat[OT] OR melon[OT] OR melons[OT] OR milk[OT] OR mollusc*[OT] OR multifood[OT] OR mushroom*[OT] OR mussel[OT] OR mussels[OT] OR mustard[OT] OR nut[OT] OR nuts[OT] OR oat[OT] OR oats[OT] OR octopus[OT] OR "oral allergy syndrome"[OT] OR orange*[OT] OR oyster[OT] OR oysters[OT] OR parsley[OT] OR pea[OT] OR peach*[OT] OR peanut*[OT] OR pear[OT] OR pears[OT] OR peas[OT] OR pecan*[OT] OR periwinkle*[OT] OR pineapple*[OT] OR pistachio*[OT] OR plum[OT] OR plums[OT] OR pork[OT] OR potato*[OT] OR prawn[OT] OR prawns[OT] OR raspberr*[OT] OR rice[OT] OR rye[OT] OR salmon[OT] OR scallop[OT] OR scallops[OT] OR seafood*[OT] OR sesame[OT] OR shellfish*[OT] OR shrimp[OT] OR shrimps[OT] OR snail[OT] OR snails[OT] OR soja[OT] OR sojabean*[OT] OR soy[OT] OR soya[OT] OR soyabean*[OT] OR soybean*[OT] OR squid[OT] OR strawberr*[OT] OR sunflower seed*[OT] OR tomato*[OT] OR vegetable*[OT] OR walnut*[OT] OR wheat[OT] OR wheats[OT] OR whelk[OT] OR whelks[OT] OR zucchini*[OT] |
| #4 | Desensitization, Immunologic[MH] OR desensitisation*[TIAB] OR desensitization*[TIAB] OR hypo-sensitisation*[TIAB] OR hyposensitisation*[TIAB] OR hypo-sensitization*[TIAB] OR hyposensitization*[TIAB] OR immunotherap*[TIAB] OR "oral induction"[TIAB] OR oral tolerance[TIAB] OR OIT[TIAB] OR up dosing[TIAB] OR desensitisation*[OT] OR desensitization*[OT] OR hypo-sensitisation*[OT] OR hyposensitisation*[OT] OR hypo-sensitization*[OT] OR hyposensitization*[OT] OR immunotherap*[OT] OR "oral induction"[OT] OR oral tolerance[OT] OR OIT[OT] OR up dosing[OT] |
| #5 | English[lang] OR French[lang] OR Italian[lang] OR Spanish[lang] OR German[lang] |
| #6 | (#1 OR (#2 AND #3)) AND #4 AND #5 |
| #7 | #6 AND ("1998/01/01"[PDAT] : "3000/12/31"[PDAT]) 2417 results |

Medline (OVID) 17 April 2019

| 1 | Exp Food Hypersensitivity/ |
| --- | --- |
| 2 | Exp Hypersensitivity/ OR (allerg* OR hypersensitiv* OR hyper-sensitiv* OR hyperresponsiv* OR hyper-responsiv*).ti,ab,kw,kf |
| 3 | Exp Food/ OR (additive OR additives OR almond* OR apple OR apples OR apricot* OR arachis hypogaea OR avocado* OR banana* OR barley OR beef OR berries OR berry OR blackberr* OR blueberr* OR buckwheat* OR cacao OR calamari OR carrot* OR cashew* OR celery OR cherries OR cherry OR chestnut* OR chicory OR chocolat* OR cilandro OR clam OR clams OR cockle* OR coconut OR coconuts OR conch OR conchs OR coriander OR corn OR crab OR crabs OR cranberr* OR crawfish* OR crayfish* OR crustacea* OR cucumber* OR dairy OR dairies OR edamame* OR egg OR eggplant* OR eggs OR fig OR figs OR fish OR fishes OR flaxseed* OR food OR foods OR fruit OR fruits OR grape OR grapes OR hazelnut* OR kiwi OR kiwis OR lactose OR legume* OR lobster OR lobsters OR mango* OR meat OR melon OR melons OR milk OR mollusc* OR multifood OR mushroom* OR mussel OR mussels OR mustard OR nut OR nuts OR oat OR oats OR octopus OR "oral allergy syndrome" OR orange* OR oyster OR oysters OR parsley OR pea OR peach* OR peanut* OR pear OR pears OR peas OR pecan* OR periwinkle* OR pineapple* OR pistachio* OR plum OR plums OR pork OR potato* OR prawn OR prawns OR raspberr* OR rice OR rye OR salmon OR scallop OR scallops OR seafood* OR sesame OR shellfish* OR shrimp OR shrimps OR snail OR snails OR soja OR sojabean* OR soy OR soya OR soyabean* OR soybean* OR squid OR strawberr* OR sunflower seed* OR tomato* OR vegetable* OR walnut* OR wheat OR wheats OR whelk OR whelks OR zucchini*).ti,ab,kw,kf |
| 4 | Exp Desensitization, Immunologic/ OR (desensitisation* OR desensitization* OR hypo-sensitisation* OR hyposensitisation* OR hypo-sensitization* OR hyposensitization* OR immunotherap* OR "oral induction" OR oral tolerance OR OIT OR up dosing).ti,ab,kw,kf |
| 5 | (English OR French OR Italian OR Spanish OR German).la |
| 6 | (1 OR (2 AND 3)) AND 4 AND 5 |
| 7 | Limit 6 to yr="1998 -Current" 2469 results |

All EBM Reviews 17 April 2019

| 1 | Exp Food Hypersensitivity/ |
| --- | --- |
| 2 | Exp Hypersensitivity/ OR (allerg* OR hypersensitiv* OR hyper-sensitiv* OR hyperresponsiv* OR hyper-responsiv*).ti,ab,kw,kf |
| 3 | Exp Food/ OR (additive OR additives OR almond* OR apple OR apples OR apricot* OR arachis hypogaea OR avocado* OR banana* OR barley OR beef OR berries OR berry OR blackberr* OR blueberr* OR buckwheat* OR cacao OR calamari OR carrot* OR cashew* OR celery OR cherries OR cherry OR chestnut* OR chicory OR chocolat* OR cilandro OR clam OR clams OR cockle* OR coconut OR coconuts OR conch OR conchs OR coriander OR corn OR crab OR crabs OR cranberr* OR crawfish* OR crayfish* OR crustacea* OR cucumber* OR dairy OR dairies OR edamame* OR egg OR eggplant* OR eggs OR fig OR figs OR fish OR fishes OR flaxseed* OR food OR foods OR fruit OR fruits OR grape OR grapes OR hazelnut* OR kiwi OR kiwis OR lactose OR legume* OR lobster OR lobsters OR mango* OR meat OR melon OR melons OR milk OR mollusc* OR multifood OR mushroom* OR mussel OR mussels OR mustard OR nut OR nuts OR oat OR oats OR octopus OR "oral allergy syndrome" OR orange* OR oyster OR oysters OR parsley OR pea OR peach* OR peanut* OR pear OR pears OR peas OR pecan* OR periwinkle* OR pineapple* OR pistachio* OR plum OR plums OR pork OR potato* OR prawn OR prawns OR raspberr* OR rice OR rye OR salmon OR scallop OR scallops OR seafood* OR sesame OR shellfish* OR shrimp OR shrimps OR snail OR snails OR soja OR sojabean* OR soy OR soya OR soyabean* OR soybean* OR squid OR strawberr* OR sunflower seed* OR tomato* OR vegetable* OR walnut* OR wheat OR wheats OR whelk OR whelks OR zucchini*).ti,ab,kw,kf |
| 4 | Exp Desensitization, Immunologic/ OR (desensitisation* OR desensitization* OR hypo-sensitisation* OR hyposensitisation* OR hypo-sensitization* OR hyposensitization* OR immunotherap* OR "oral induction" OR oral tolerance OR OIT OR up dosing).ti,ab,kw,kf |
| 5 | (English OR French OR Italian OR Spanish OR German).la |
| 6 | (1 OR (2 AND 3)) AND 4 AND 5 |
| 7 | Limit 6 to yr="1998 -Current" 432 results |

Embase 18 April 2019

| 1 | Exp food allergy OR food allergen/ |
| --- | --- |
| 2 | Exp Hypersensitivity/ OR (allerg* OR hypersensitiv* OR hyper-sensitiv* OR hyperresponsiv* OR hyper-responsiv*).ti,ab,kw |
| 3 | Exp food/ OR (additive OR additives OR almond* OR apple OR apples OR apricot* OR arachis hypogaea OR avocado* OR banana* OR barley OR beef OR berries OR berry OR blackberr* OR blueberr* OR buckwheat* OR cacao OR calamari OR carrot* OR cashew* OR celery OR cherries OR cherry OR chestnut* OR chicory OR chocolat* OR cilandro OR clam OR clams OR cockle* OR coconut OR coconuts OR conch OR conchs OR coriander OR corn OR crab OR crabs OR cranberr* OR crawfish* OR crayfish* OR crustacea* OR cucumber* OR dairy OR dairies OR edamame* OR egg OR eggplant* OR eggs OR fig OR figs OR fish OR fishes OR flaxseed* OR food OR foods OR fruit OR fruits OR grape OR grapes OR hazelnut* OR kiwi OR kiwis OR lactose OR legume* OR lobster OR lobsters OR mango* OR meat OR melon OR melons OR milk OR mollusc* OR multifood OR mushroom* OR mussel OR mussels OR mustard OR nut OR nuts OR oat OR oats OR octopus OR "oral allergy syndrome" OR orange* OR oyster OR oysters OR parsley OR pea OR peach* OR peanut* OR pear OR pears OR peas OR pecan* OR periwinkle* OR pineapple* OR pistachio* OR plum OR plums OR pork OR potato* OR prawn OR prawns OR raspberr* OR rice OR rye OR salmon OR scallop OR scallops OR seafood* OR sesame OR shellfish* OR shrimp OR shrimps OR snail OR snails OR soja OR sojabean* OR soy OR soya OR soyabean* OR soybean* OR squid OR strawberr* OR sunflower seed* OR tomato* OR vegetable* OR walnut* OR wheat OR wheats OR whelk OR whelks OR zucchini*).ti,ab,kw |
| 4 | Exp desensitization/ OR (desensitisation* OR desensitization* OR hypo-sensitisation* OR hyposensitisation* OR hypo-sensitization* OR hyposensitization* OR immunotherap* OR "oral induction" OR oral tolerance OR OIT OR up dosing).ti,ab,kw |
| 5 | (English OR French OR Italian OR Spanish OR German).la |
| 6 | (1 OR (2 AND 3)) AND 4 AND 5 |
| 7 | Limit 6 to yr="1998 -Current" |
| 8 | conference abstract.pt. |
| 9 | 7 not 8 3260 results |

CINAHL 18 April 2019

| S1 | MH(Food Hypersensitivity+) |
| --- | --- |
| S2 | MH(Hypersensitivity+) OR TI(allerg* OR hypersensitiv* OR hyper-sensitiv* OR hyperresponsiv* OR hyper-responsiv*) OR AB(allerg* OR hypersensitiv* OR hyper-sensitiv* OR hyperresponsiv* OR hyper-responsiv*) |
| S3 | MH(food+) OR TI(additive OR additives OR almond* OR apple OR apples OR apricot* OR arachis hypogaea OR avocado* OR banana* OR barley OR beef OR berries OR berry OR blackberr* OR blueberr* OR buckwheat* OR cacao OR calamari OR carrot* OR cashew* OR celery OR cherries OR cherry OR chestnut* OR chicory OR chocolat* OR cilandro OR clam OR clams OR cockle* OR coconut OR coconuts OR conch OR conchs OR coriander OR corn OR crab OR crabs OR cranberr* OR crawfish* OR crayfish* OR crustacea* OR cucumber* OR dairy OR dairies OR edamame* OR egg OR eggplant* OR eggs OR fig OR figs OR fish OR fishes OR flaxseed* OR food OR foods OR fruit OR fruits OR grape OR grapes OR hazelnut* OR kiwi OR kiwis OR lactose OR legume* OR lobster OR lobsters OR mango* OR meat OR melon OR melons OR milk OR mollusc* OR multifood OR mushroom* OR mussel OR mussels OR mustard OR nut OR nuts OR oat OR oats OR octopus OR "oral allergy syndrome" OR orange* OR oyster OR oysters OR parsley OR pea OR peach* OR peanut* OR pear OR pears OR peas OR pecan* OR periwinkle* OR pineapple* OR pistachio* OR plum OR plums OR pork OR potato* OR prawn OR prawns OR raspberr* OR rice OR rye OR salmon OR scallop OR scallops OR seafood* OR sesame OR shellfish* OR shrimp OR shrimps OR snail OR snails OR soja OR sojabean* OR soy OR soya OR soyabean* OR soybean* OR squid OR strawberr* OR sunflower seed* OR tomato* OR vegetable* OR walnut* OR wheat OR wheats OR whelk OR whelks OR zucchini*) OR AB(additive OR additives OR almond* OR apple OR apples OR apricot* OR arachis hypogaea OR avocado* OR banana* OR barley OR beef OR berries OR berry OR blackberr* OR blueberr* OR buckwheat* OR cacao OR calamari OR carrot* OR cashew* OR celery OR cherries OR cherry OR chestnut* OR chicory OR chocolat* OR cilandro OR clam OR clams OR cockle* OR coconut OR coconuts OR conch OR conchs OR coriander OR corn OR crab OR crabs OR cranberr* OR crawfish* OR crayfish* OR crustacea* OR cucumber* OR dairy OR dairies OR edamame* OR egg OR eggplant* OR eggs OR fig OR figs OR fish OR fishes OR flaxseed* OR food OR foods OR fruit OR fruits OR grape OR grapes OR hazelnut* OR kiwi OR kiwis OR lactose OR legume* OR lobster OR lobsters OR mango* OR meat OR melon OR melons OR milk OR mollusc* OR multifood OR mushroom* OR mussel OR mussels OR mustard OR nut OR nuts OR oat OR oats OR octopus OR "oral allergy syndrome" OR orange* OR oyster OR oysters OR parsley OR pea OR peach* OR peanut* OR pear OR pears OR peas OR pecan* OR periwinkle* OR pineapple* OR pistachio* OR plum OR plums OR pork OR potato* OR prawn OR prawns OR raspberr* OR rice OR rye OR salmon OR scallop OR scallops OR seafood* OR sesame OR shellfish* OR shrimp OR shrimps OR snail OR snails OR soja OR sojabean* OR soy OR soya OR soyabean* OR soybean* OR squid OR strawberr* OR sunflower seed* OR tomato* OR vegetable* OR walnut* OR wheat OR wheats OR whelk OR whelks OR zucchini*) |
| S4 | MH(Desensitization, Immunologic) OR TI(desensitisation* OR desensitization* OR hypo-sensitisation* OR hyposensitisation* OR hypo-sensitization* OR hyposensitization* OR immunotherap* OR "oral induction" OR oral tolerance OR OIT OR up dosing) OR AB(desensitisation* OR desensitization* OR hypo-sensitisation* OR hyposensitisation* OR hypo-sensitization* OR hyposensitization* OR immunotherap* OR "oral induction" OR oral tolerance OR OIT OR up dosing) |
| S5 | LA(English OR French OR Italian OR Spanish OR German) |
| S6 | (S1 OR (S2 AND S3)) AND S4 AND S5 **limiters** publication date 19980101-20191231 437 results |

Web of Science 18 April 2019

| #1 | TS=( allerg* OR hypersensitiv* OR hyper-sensitiv* OR hyperresponsiv* OR hyper-responsiv*) |
| --- | --- |
| #2 | TS=(additive OR additives OR almond* OR apple OR apples OR apricot* OR arachis hypogaea OR avocado* OR banana* OR barley OR beef OR berries OR berry OR blackberr* OR blueberr* OR buckwheat* OR cacao OR calamari OR carrot* OR cashew* OR celery OR cherries OR cherry OR chestnut* OR chicory OR chocolat* OR cilandro OR clam OR clams OR cockle* OR coconut OR coconuts OR conch OR conchs OR coriander OR corn OR crab OR crabs OR cranberr* OR crawfish* OR crayfish* OR crustacea* OR cucumber* OR dairy OR dairies OR edamame* OR egg OR eggplant* OR eggs OR fig OR figs OR fish OR fishes OR flaxseed* OR food OR foods OR fruit OR fruits OR grape OR grapes OR hazelnut* OR kiwi OR kiwis OR lactose OR legume* OR lobster OR lobsters OR mango* OR meat OR melon OR melons OR milk OR mollusc* OR multifood OR mushroom* OR mussel OR mussels OR mustard OR nut OR nuts OR oat OR oats OR octopus OR "oral allergy syndrome" OR orange* OR oyster OR oysters OR parsley OR pea OR peach* OR peanut* OR pear OR pears OR peas OR pecan* OR periwinkle* OR pineapple* OR pistachio* OR plum OR plums OR pork OR potato* OR prawn OR prawns OR raspberr* OR rice OR rye OR salmon OR scallop OR scallops OR seafood* OR sesame OR shellfish* OR shrimp OR shrimps OR snail OR snails OR soja OR sojabean* OR soy OR soya OR soyabean* OR soybean* OR squid OR strawberr* OR sunflower seed* OR tomato* OR vegetable* OR walnut* OR wheat OR wheats OR whelk OR whelks OR zucchini*) |
| #3 | TS=(desensitisation* OR desensitization* OR hypo-sensitisation* OR hyposensitisation* OR hypo-sensitization* OR hyposensitization* OR immunotherap* OR "oral induction" OR oral tolerance OR OIT OR up dosing) |
| #4 | (#1 AND #2 AND #3) AND LANGUAGE: (English OR French OR German OR Italian OR Spanish) Timespan=1998-2019 4121 results |

After the removal of duplicates, there were a total of 5556 records retrieved on Avril 18^th^ 2019.

### Second search: Epidemiology, burden of illness, impact on quality of life

PubMed 30 Mai 2019

| #1 | Food Hypersensitivity[MH] |
| --- | --- |
| #2 | Hypersensitivity[MH] OR allerg*[TIAB] OR hypersensitiv*[TIAB] OR hyper-sensitiv*[TIAB] OR hyperresponsiv*[TIAB] OR hyper-responsiv*[TIAB] OR allerg*[OT] OR hypersensitiv*[OT] OR hyper-sensitiv*[OT] OR hyperresponsiv*[OT] OR hyper-responsiv*[OT] |
| #3 | Food[Mesh] OR additive[TIAB] OR additives[TIAB] OR almond*[TIAB] OR apple[TIAB] OR apples[TIAB] OR apricot*[TIAB] OR arachis hypogaea[TIAB] OR avocado*[TIAB] OR banana*[TIAB] OR barley[TIAB] OR beef[TIAB] OR berries[TIAB] OR berry[TIAB] OR blackberr*[TIAB] OR blueberr*[TIAB] OR buckwheat*[TIAB] OR cacao[TIAB] OR calamari[TIAB] OR carrot*[TIAB] OR cashew*[TIAB] OR celery[TIAB] OR cherries[TIAB] OR cherry[TIAB] OR chestnut*[TIAB] OR chicory[TIAB] OR chocolat*[TIAB] OR cilandro[TIAB] OR clam[TIAB] OR clams[TIAB] OR cockle*[TIAB] OR coconut[TIAB] OR coconuts[TIAB] OR conch[TIAB] OR conchs[TIAB] OR coriander[TIAB] OR corn[TIAB] OR crab[TIAB] OR crabs[TIAB] OR cranberr*[TIAB] OR crawfish*[TIAB] OR crayfish*[TIAB] OR crustacea*[TIAB] OR cucumber*[TIAB] OR dairy[TIAB] OR dairies[TIAB] OR edamame*[TIAB] OR egg[TIAB] OR eggplant*[TIAB] OR eggs[TIAB] OR fig[TIAB] OR figs[TIAB] OR fish[TIAB] OR fishes[TIAB] OR flaxseed*[TIAB] OR food[TIAB] OR foods[TIAB] OR fruit[TIAB] OR fruits[TIAB] OR grape[TIAB] OR grapes[TIAB] OR hazelnut*[TIAB] OR kiwi[TIAB] OR kiwis[TIAB] OR lactose[TIAB] OR legume*[TIAB] OR lobster[TIAB] OR lobsters[TIAB] OR mango*[TIAB] OR meat[TIAB] OR melon[TIAB] OR melons[TIAB] OR milk[TIAB] OR mollusc*[TIAB] OR multifood[TIAB] OR mushroom*[TIAB] OR mussel[TIAB] OR mussels[TIAB] OR mustard[TIAB] OR nut[TIAB] OR nuts[TIAB] OR oat[TIAB] OR oats[TIAB] OR octopus[TIAB] OR "oral allergy syndrome"[TIAB] OR orange*[TIAB] OR oyster[TIAB] OR oysters[TIAB] OR parsley[TIAB] OR pea[TIAB] OR peach*[TIAB] OR peanut*[TIAB] OR pear[TIAB] OR pears[TIAB] OR peas[TIAB] OR pecan*[TIAB] OR periwinkle*[TIAB] OR pineapple*[TIAB] OR pistachio*[TIAB] OR plum[TIAB] OR plums[TIAB] OR pork[TIAB] OR potato*[TIAB] OR prawn[TIAB] OR prawns[TIAB] OR raspberr*[TIAB] OR rice[TIAB] OR rye[TIAB] OR salmon[TIAB] OR scallop[TIAB] OR scallops[TIAB] OR seafood*[TIAB] OR sesame[TIAB] OR shellfish*[TIAB] OR shrimp[TIAB] OR shrimps[TIAB] OR snail[TIAB] OR snails[TIAB] OR soja[TIAB] OR sojabean*[TIAB] OR soy[TIAB] OR soya[TIAB] OR soyabean*[TIAB] OR soybean*[TIAB] OR squid[TIAB] OR strawberr*[TIAB] OR sunflower seed*[TIAB] OR tomato*[TIAB] OR vegetable*[TIAB] OR walnut*[TIAB] OR wheat[TIAB] OR wheats[TIAB] OR whelk[TIAB] OR whelks[TIAB] OR zucchini*[TIAB] OR additive[OT] OR additives[OT] OR almond*[OT] OR apple[OT] OR apples[OT] OR apricot*[OT] OR arachis hypogaea[OT] OR avocado*[OT] OR banana*[OT] OR barley[OT] OR beef[OT] OR berries[OT] OR berry[OT] OR blackberr*[OT] OR blueberr*[OT] OR buckwheat*[OT] OR cacao[OT] OR calamari[OT] OR carrot*[OT] OR cashew*[OT] OR celery[OT] OR cherries[OT] OR cherry[OT] OR chestnut*[OT] OR chicory[OT] OR chocolat*[OT] OR cilandro[OT] OR clam[OT] OR clams[OT] OR cockle*[OT] OR coconut[OT] OR coconuts[OT] OR conch[OT] OR conchs[OT] OR coriander[OT] OR corn[OT] OR crab[OT] OR crabs[OT] OR cranberr*[OT] OR crawfish*[OT] OR crayfish*[OT] OR crustacea*[OT] OR cucumber*[OT] OR dairy[OT] OR dairies[OT] OR edamame*[OT] OR egg[OT] OR eggplant*[OT] OR eggs[OT] OR fig[OT] OR figs[OT] OR fish[OT] OR fishes[OT] OR flaxseed*[OT] OR food[OT] OR foods[OT] OR fruit[OT] OR fruits[OT] OR grape[OT] OR grapes[OT] OR hazelnut*[OT] OR kiwi[OT] OR kiwis[OT] OR lactose[OT] OR legume*[OT] OR lobster[OT] OR lobsters[OT] OR mango*[OT] OR meat[OT] OR melon[OT] OR melons[OT] OR milk[OT] OR mollusc*[OT] OR multifood[OT] OR mushroom*[OT] OR mussel[OT] OR mussels[OT] OR mustard[OT] OR nut[OT] OR nuts[OT] OR oat[OT] OR oats[OT] OR octopus[OT] OR "oral allergy syndrome"[OT] OR orange*[OT] OR oyster[OT] OR oysters[OT] OR parsley[OT] OR pea[OT] OR peach*[OT] OR peanut*[OT] OR pear[OT] OR pears[OT] OR peas[OT] OR pecan*[OT] OR periwinkle*[OT] OR pineapple*[OT] OR pistachio*[OT] OR plum[OT] OR plums[OT] OR pork[OT] OR potato*[OT] OR prawn[OT] OR prawns[OT] OR raspberr*[OT] OR rice[OT] OR rye[OT] OR salmon[OT] OR scallop[OT] OR scallops[OT] OR seafood*[OT] OR sesame[OT] OR shellfish*[OT] OR shrimp[OT] OR shrimps[OT] OR snail[OT] OR snails[OT] OR soja[OT] OR sojabean*[OT] OR soy[OT] OR soya[OT] OR soyabean*[OT] OR soybean*[OT] OR squid[OT] OR strawberr*[OT] OR sunflower seed*[OT] OR tomato*[OT] OR vegetable*[OT] OR walnut*[OT] OR wheat[OT] OR wheats[OT] OR whelk[OT] OR whelks[OT] OR zucchini*[OT] |
| #4 | Psychology[Mh:noexp] OR Psychology[sh] OR Stress, Psychological[Mh] OR Adaptation, Psychological[Mh:noexp] OR Quality of Life[Mh] OR Family Health[Mh] OR Social*[TIAB] OR Psychosocial*[TIAB] OR Psycholog*[TIAB] OR Quality of Life[TIAB] OR Life quality[TIAB] OR QoL[TIAB] OR HRQoL[TIAB] OR burden*[TIAB] OR Social*[OT] OR Psychosocial*[OT] OR Psycholog*[OT] OR Quality of Life[OT] OR Life quality[OT] OR QoL[OT] OR HRQoL[OT] OR burden*[OT] |
| #5 | Epidemiology[mh] OR Epidemiology[sh:noexp] OR Incidence[Mh] OR Prevalence[Mh] OR "Costs and Cost Analysis"[Mh] OR economics[sh] OR Epidemiolog*[TIAB] OR prevalence*[TIAB] OR Frequenc*[TIAB] OR occurrence*[TIAB] OR incidence*[TIAB] OR cost*[tiab] OR expens*[tiab] OR expenditure*[tiab] OR economic*[tiab] OR financ*[tiab] OR budget*[tiab] OR spending*[tiab] OR Epidemiolog*[OT] OR prevalence*[OT] OR Frequenc*[OT] OR occurrence*[OT] OR incidence*[OT] OR cost*[OT] OR expens*[OT] OR expenditure*[OT] OR economic*[OT] OR financ*[OT] OR budget*[OT] OR spending*[OT] |
| #6 | Canada[Mesh] OR Canad*[TIAB] OR Alberta*[TIAB] OR Calgary[TIAB] OR Edmonton[TIAB] OR British Columbi*[TIAB] OR Vancouver[TIAB] OR Manitoba*[TIAB] OR New Brunswick[TIAB] OR Newfoundland[TIAB] OR Labrador[TIAB] OR Northwest Territor*[TIAB] OR Nova Scotia*[TIAB] OR Nunavut[TIAB] OR Ontari*[TIAB] OR Toronto[TIAB] OR Ottawa[TIAB] OR Prince Edward Island[TIAB] OR Quebec*[TIAB] OR Montreal[TIAB] OR Saskatchewan[TIAB] OR Yukon[TIAB] OR Canad*[OT] OR Alberta*[OT] OR Calgary[OT] OR Edmonton[OT] OR British Columbi*[OT] OR Vancouver[OT] OR Manitoba*[OT] OR New Brunswick[OT] OR Newfoundland[OT] OR Labrador[OT] OR Northwest Territor*[OT] OR Nova Scotia*[OT] OR Nunavut[OT] OR Ontari*[OT] OR Toronto[OT] OR Ottawa[OT] OR Prince Edward Island[OT] OR Quebec*[OT] OR Montreal[OT] OR Saskatchewan[OT] OR Yukon[OT] |
| #7 | Systematic review[Pt] OR Systematic Reviews as Topic[Mh] OR Meta-Analysis[Pt] OR Meta-Analysis as Topic[mh] OR ((systematic*[tiab] OR Umbrella[tiab]) AND (review*[tiab] OR overview*[tiab] OR synthes*[tiab])) OR meta-analys*[tiab] OR metaanalys*[tiab] OR ((systematic*[OT] OR umbrella[OT]) AND (review*[OT] OR overview*[OT] OR synthes*[OT])) OR meta-analys*[OT] OR metaanalys*[OT] |
| #8 | English[lang] OR French[lang] OR Italian[lang] OR Spanish[lang] OR German[lang] |
| #9 | (#1 OR (#2 AND #3)) AND (#4 OR (#5 AND (#6 OR #7))) AND #8 |
| #10 | #9 AND ("2014/01/01"[PDAT] : "2019/05/30"[PDAT]) 1233 results |

Medline (OVID) 30 Mai 2019

| 1 | Exp Food Hypersensitivity/ | |
| --- | --- | --- |
| 2 | Exp Hypersensitivity/ OR (allerg* OR hypersensitiv* OR hyper-sensitiv* OR hyperresponsiv* OR hyper-responsiv*).ti,ab,kw,kf | |
| 3 | Exp Food/ OR (additive OR additives OR almond* OR apple OR apples OR apricot* OR arachis hypogaea OR avocado* OR banana* OR barley OR beef OR berries OR berry OR blackberr* OR blueberr* OR buckwheat* OR cacao OR calamari OR carrot* OR cashew* OR celery OR cherries OR cherry OR chestnut* OR chicory OR chocolat* OR cilandro OR clam OR clams OR cockle* OR coconut OR coconuts OR conch OR conchs OR coriander OR corn OR crab OR crabs OR cranberr* OR crawfish* OR crayfish* OR crustacea* OR cucumber* OR dairy OR dairies OR edamame* OR egg OR eggplant* OR eggs OR fig OR figs OR fish OR fishes OR flaxseed* OR food OR foods OR fruit OR fruits OR grape OR grapes OR hazelnut* OR kiwi OR kiwis OR lactose OR legume* OR lobster OR lobsters OR mango* OR meat OR melon OR melons OR milk OR mollusc* OR multifood OR mushroom* OR mussel OR mussels OR mustard OR nut OR nuts OR oat OR oats OR octopus OR "oral allergy syndrome" OR orange* OR oyster OR oysters OR parsley OR pea OR peach* OR peanut* OR pear OR pears OR peas OR pecan* OR periwinkle* OR pineapple* OR pistachio* OR plum OR plums OR pork OR potato* OR prawn OR prawns OR raspberr* OR rice OR rye OR salmon OR scallop OR scallops OR seafood* OR sesame OR shellfish* OR shrimp OR shrimps OR snail OR snails OR soja OR sojabean* OR soy OR soya OR soyabean* OR soybean* OR squid OR strawberr* OR sunflower seed* OR tomato* OR vegetable* OR walnut* OR wheat OR wheats OR whelk OR whelks OR zucchini*).ti,ab,kw,kf | |
| 4 | Psychology/ OR Psychology.fs OR exp Stress, Psychological/ OR Adaptation, Psychological/ OR exp Quality of Life/ OR exp Family Health/ OR (Social* OR Psychosocial* OR Psycholog* OR Quality of Life OR Life quality OR QoL OR HRQoL OR burden*).ti,ab,kw,kf |  |
| 5 | Exp Epidemiology/ OR Epidemiology.fs OR exp Incidence/ OR exp Prevalence/ OR exp "Costs and Cost Analysis"/ OR economics.fs OR (Epidemiolog* OR prevalence* OR Frequenc* OR occurrence* OR incidence* OR cost* OR expens* OR expenditure* OR economic* OR financ* OR budget* OR spending*).ti,ab,kw,kf |  |
| 6 | Exp Canada/ OR ( Canad* OR Alberta* OR Calgary OR Edmonton OR British Columbi* OR Vancouver OR Manitoba* OR New Brunswick OR Newfoundland OR Labrador OR Northwest Territor* OR Nova Scotia* OR Nunavut OR Ontari* OR Toronto OR Ottawa OR Prince Edward Island OR Quebec* OR Montreal OR Saskatchewan OR Yukon).ti,ab,kw,kf |  |
| 7 | Systematic review.pt OR Systematic Reviews as Topic/ OR Meta-Analysis.pt OR Meta-Analysis as Topic/ OR (((systematic* OR Umbrella) AND (review* OR overview* OR synthes*)) OR meta-analys* OR metaanalys*).ti,ab,kw,kf |  |
| 8 | (English OR French OR Italian OR Spanish OR German).lg |  |
| 9 | (1 OR (2 AND 3)) AND (4 OR (5 AND (6 OR 7))) AND 8 |  |
| 10 | limit 9 to yr="2014 - 2019" 1298 results |  |

All EBM Reviews 30 Mai 2019

| 1 | Exp Food Hypersensitivity/ | |
| --- | --- | --- |
| 2 | Exp Hypersensitivity/ OR (allerg* OR hypersensitiv* OR hyper-sensitiv* OR hyperresponsiv* OR hyper-responsiv*).ti,ab,kw,kf | |
| 3 | Exp Food/ OR (additive OR additives OR almond* OR apple OR apples OR apricot* OR arachis hypogaea OR avocado* OR banana* OR barley OR beef OR berries OR berry OR blackberr* OR blueberr* OR buckwheat* OR cacao OR calamari OR carrot* OR cashew* OR celery OR cherries OR cherry OR chestnut* OR chicory OR chocolat* OR cilandro OR clam OR clams OR cockle* OR coconut OR coconuts OR conch OR conchs OR coriander OR corn OR crab OR crabs OR cranberr* OR crawfish* OR crayfish* OR crustacea* OR cucumber* OR dairy OR dairies OR edamame* OR egg OR eggplant* OR eggs OR fig OR figs OR fish OR fishes OR flaxseed* OR food OR foods OR fruit OR fruits OR grape OR grapes OR hazelnut* OR kiwi OR kiwis OR lactose OR legume* OR lobster OR lobsters OR mango* OR meat OR melon OR melons OR milk OR mollusc* OR multifood OR mushroom* OR mussel OR mussels OR mustard OR nut OR nuts OR oat OR oats OR octopus OR "oral allergy syndrome" OR orange* OR oyster OR oysters OR parsley OR pea OR peach* OR peanut* OR pear OR pears OR peas OR pecan* OR periwinkle* OR pineapple* OR pistachio* OR plum OR plums OR pork OR potato* OR prawn OR prawns OR raspberr* OR rice OR rye OR salmon OR scallop OR scallops OR seafood* OR sesame OR shellfish* OR shrimp OR shrimps OR snail OR snails OR soja OR sojabean* OR soy OR soya OR soyabean* OR soybean* OR squid OR strawberr* OR sunflower seed* OR tomato* OR vegetable* OR walnut* OR wheat OR wheats OR whelk OR whelks OR zucchini*).ti,ab,kw,kf | |
| 4 | Psychology/ OR Psychology.fs OR exp Stress, Psychological/ OR Adaptation, Psychological/ OR exp Quality of Life/ OR exp Family Health/ OR (Social* OR Psychosocial* OR Psycholog* OR Quality of Life OR Life quality OR QoL OR HRQoL OR burden*).ti,ab,kw,kf |  |
| 5 | Exp Epidemiology/ OR Epidemiology.fs OR exp Incidence/ OR exp Prevalence/ OR exp "Costs and Cost Analysis"/ OR economics.fs OR (Epidemiolog* OR prevalence* OR Frequenc* OR occurrence* OR incidence* OR cost* OR expens* OR expenditure* OR economic* OR financ* OR budget* OR spending*).ti,ab,kw,kf |  |
| 6 | Exp Canada/ OR ( Canad* OR Alberta* OR Calgary OR Edmonton OR British Columbi* OR Vancouver OR Manitoba* OR New Brunswick OR Newfoundland OR Labrador OR Northwest Territor* OR Nova Scotia* OR Nunavut OR Ontari* OR Toronto OR Ottawa OR Prince Edward Island OR Quebec* OR Montreal OR Saskatchewan OR Yukon).ti,ab,kw,kf |  |
| 7 | Systematic review.pt OR Systematic Reviews as Topic/ OR Meta-Analysis.pt OR Meta-Analysis as Topic/ OR (((systematic* OR Umbrella) AND (review* OR overview* OR synthes*)) OR meta-analys* OR metaanalys*).ti,ab,kw,kf |  |
| 8 | (English OR French OR Italian OR Spanish OR German).lg |  |
| 9 | (1 OR (2 AND 3)) AND (4 OR (5 AND (6 OR 7))) AND 8 |  |
| 10 | limit 9 to yr="2014 - 2019" 170 results |  |

Embase 31 Mai 2019

| 1 | Exp food allergy OR food allergen/ |
| --- | --- |
| 2 | Exp Hypersensitivity/ OR (allerg* OR hypersensitiv* OR hyper-sensitiv* OR hyperresponsiv* OR hyper-responsiv*).ti,ab,kw |
| 3 | Exp food/ OR (additive OR additives OR almond* OR apple OR apples OR apricot* OR arachis hypogaea OR avocado* OR banana* OR barley OR beef OR berries OR berry OR blackberr* OR blueberr* OR buckwheat* OR cacao OR calamari OR carrot* OR cashew* OR celery OR cherries OR cherry OR chestnut* OR chicory OR chocolat* OR cilandro OR clam OR clams OR cockle* OR coconut OR coconuts OR conch OR conchs OR coriander OR corn OR crab OR crabs OR cranberr* OR crawfish* OR crayfish* OR crustacea* OR cucumber* OR dairy OR dairies OR edamame* OR egg OR eggplant* OR eggs OR fig OR figs OR fish OR fishes OR flaxseed* OR food OR foods OR fruit OR fruits OR grape OR grapes OR hazelnut* OR kiwi OR kiwis OR lactose OR legume* OR lobster OR lobsters OR mango* OR meat OR melon OR melons OR milk OR mollusc* OR multifood OR mushroom* OR mussel OR mussels OR mustard OR nut OR nuts OR oat OR oats OR octopus OR "oral allergy syndrome" OR orange* OR oyster OR oysters OR parsley OR pea OR peach* OR peanut* OR pear OR pears OR peas OR pecan* OR periwinkle* OR pineapple* OR pistachio* OR plum OR plums OR pork OR potato* OR prawn OR prawns OR raspberr* OR rice OR rye OR salmon OR scallop OR scallops OR seafood* OR sesame OR shellfish* OR shrimp OR shrimps OR snail OR snails OR soja OR sojabean* OR soy OR soya OR soyabean* OR soybean* OR squid OR strawberr* OR sunflower seed* OR tomato* OR vegetable* OR walnut* OR wheat OR wheats OR whelk OR whelks OR zucchini*).ti,ab,kw |
| 4 | Psychology/ OR metal stress/ OR exp coping behavior/ OR exp "Quality of Life"/ OR Family Health/ OR (Social* OR Psychosocial* OR Psycholog* OR Quality of Life OR Life quality OR QoL OR HRQoL OR burden*).ti,ab,kw |
| 5 | Epidemiology/ OR ep.fs OR exp Incidence/ OR exp Prevalence/ OR exp Cost/ OR (Epidemiolog* OR prevalence* OR Frequenc* OR occurrence* OR incidence* OR cost* OR expens* OR expenditure* OR economic* OR financ* OR budget* OR spending*).ti,ab,kw |
| 6 | Exp Canada/ OR ( Canad* OR Alberta* OR Calgary OR Edmonton OR British Columbi* OR Vancouver OR Manitoba* OR New Brunswick OR Newfoundland OR Labrador OR Northwest Territor* OR Nova Scotia* OR Nunavut OR Ontari* OR Toronto OR Ottawa OR Prince Edward Island OR Quebec* OR Montreal OR Saskatchewan OR Yukon).ti,ab,kw |
| 7 | "systematic review (topic)"/ OR " Meta-Analysis (topic)"/ OR (((systematic* OR Umbrella) AND (review* OR overview* OR synthes*)) OR meta-analys* OR metaanalys*).ti,ab,kw |
| 8 | limit 5 to (meta analysis or "systematic review") |
| 9 | (English OR French OR Italian OR Spanish OR German).la |
| 10 | (1 OR (2 AND 3)) AND (4 OR 8 OR (5 AND (6 OR 7))) AND 9 |
| 11 | limit 10 to yr="2014 - 2019" |
| 12 | conference abstract.pt. |
| 13 | 11 not 12 1710 results |

CINAHL 31 Mai 2019

| S1 | MH(Food Hypersensitivity+) |
| --- | --- |
| S2 | MH(Hypersensitivity+) OR TI(allerg* OR hypersensitiv* OR hyper-sensitiv* OR hyperresponsiv* OR hyper-responsiv*) OR AB(allerg* OR hypersensitiv* OR hyper-sensitiv* OR hyperresponsiv* OR hyper-responsiv*) |
| S3 | MH(food+) OR TI(additive OR additives OR almond* OR apple OR apples OR apricot* OR arachis hypogaea OR avocado* OR banana* OR barley OR beef OR berries OR berry OR blackberr* OR blueberr* OR buckwheat* OR cacao OR calamari OR carrot* OR cashew* OR celery OR cherries OR cherry OR chestnut* OR chicory OR chocolat* OR cilandro OR clam OR clams OR cockle* OR coconut OR coconuts OR conch OR conchs OR coriander OR corn OR crab OR crabs OR cranberr* OR crawfish* OR crayfish* OR crustacea* OR cucumber* OR dairy OR dairies OR edamame* OR egg OR eggplant* OR eggs OR fig OR figs OR fish OR fishes OR flaxseed* OR food OR foods OR fruit OR fruits OR grape OR grapes OR hazelnut* OR kiwi OR kiwis OR lactose OR legume* OR lobster OR lobsters OR mango* OR meat OR melon OR melons OR milk OR mollusc* OR multifood OR mushroom* OR mussel OR mussels OR mustard OR nut OR nuts OR oat OR oats OR octopus OR "oral allergy syndrome" OR orange* OR oyster OR oysters OR parsley OR pea OR peach* OR peanut* OR pear OR pears OR peas OR pecan* OR periwinkle* OR pineapple* OR pistachio* OR plum OR plums OR pork OR potato* OR prawn OR prawns OR raspberr* OR rice OR rye OR salmon OR scallop OR scallops OR seafood* OR sesame OR shellfish* OR shrimp OR shrimps OR snail OR snails OR soja OR sojabean* OR soy OR soya OR soyabean* OR soybean* OR squid OR strawberr* OR sunflower seed* OR tomato* OR vegetable* OR walnut* OR wheat OR wheats OR whelk OR whelks OR zucchini*) OR AB(additive OR additives OR almond* OR apple OR apples OR apricot* OR arachis hypogaea OR avocado* OR banana* OR barley OR beef OR berries OR berry OR blackberr* OR blueberr* OR buckwheat* OR cacao OR calamari OR carrot* OR cashew* OR celery OR cherries OR cherry OR chestnut* OR chicory OR chocolat* OR cilandro OR clam OR clams OR cockle* OR coconut OR coconuts OR conch OR conchs OR coriander OR corn OR crab OR crabs OR cranberr* OR crawfish* OR crayfish* OR crustacea* OR cucumber* OR dairy OR dairies OR edamame* OR egg OR eggplant* OR eggs OR fig OR figs OR fish OR fishes OR flaxseed* OR food OR foods OR fruit OR fruits OR grape OR grapes OR hazelnut* OR kiwi OR kiwis OR lactose OR legume* OR lobster OR lobsters OR mango* OR meat OR melon OR melons OR milk OR mollusc* OR multifood OR mushroom* OR mussel OR mussels OR mustard OR nut OR nuts OR oat OR oats OR octopus OR "oral allergy syndrome" OR orange* OR oyster OR oysters OR parsley OR pea OR peach* OR peanut* OR pear OR pears OR peas OR pecan* OR periwinkle* OR pineapple* OR pistachio* OR plum OR plums OR pork OR potato* OR prawn OR prawns OR raspberr* OR rice OR rye OR salmon OR scallop OR scallops OR seafood* OR sesame OR shellfish* OR shrimp OR shrimps OR snail OR snails OR soja OR sojabean* OR soy OR soya OR soyabean* OR soybean* OR squid OR strawberr* OR sunflower seed* OR tomato* OR vegetable* OR walnut* OR wheat OR wheats OR whelk OR whelks OR zucchini*) |
| S4 | MH(Psychology) OR MW(PH) OR Mh(Stress, Psychological+) OR MH(Adaptation, Psychological+) OR MH(Quality of Life+) OR MH(Family Health) OR TI(Social* OR Psychosocial* OR Psycholog* OR Quality of Life OR Life quality OR QoL OR HRQoL OR burden*) OR AB(Social* OR Psychosocial* OR Psycholog* OR Quality of Life OR Life quality OR QoL OR HRQoL OR burden*) |
| S5 | MH(Epidemiology+) OR MW(EP) OR MH(Incidence) OR MH(Prevalence) OR MH("Costs and Cost Analysis"+) OR MW(EC) OR TI(Epidemiolog* OR prevalence* OR Frequenc* OR occurrence* OR incidence* OR cost* OR expens* OR expenditure* OR economic* OR financ* OR budget* OR spending*) OR AB(Epidemiolog* OR prevalence* OR Frequenc* OR occurrence* OR incidence* OR cost* OR expens* OR expenditure* OR economic* OR financ* OR budget* OR spending*) |
| S6 | MH(Canada+) OR TI( Canad* OR Alberta* OR Calgary OR Edmonton OR British Columbi* OR Vancouver OR Manitoba* OR New Brunswick OR Newfoundland OR Labrador OR Northwest Territor* OR Nova Scotia* OR Nunavut OR Ontari* OR Toronto OR Ottawa OR Prince Edward Island OR Quebec* OR Montreal OR Saskatchewan OR Yukon) OR AB( Canad* OR Alberta* OR Calgary OR Edmonton OR British Columbi* OR Vancouver OR Manitoba* OR New Brunswick OR Newfoundland OR Labrador OR Northwest Territor* OR Nova Scotia* OR Nunavut OR Ontari* OR Toronto OR Ottawa OR Prince Edward Island OR Quebec* OR Montreal OR Saskatchewan OR Yukon) |
| S7 | PT(Systematic review) OR MH( Systematic Review) OR PT(Meta-Analysis) OR MH(Meta-Analysis) OR TI(((systematic* OR Umbrella) AND (review* OR overview* OR synthes*)) OR meta-analys* OR metaanalys*) OR AB(((systematic* OR Umbrella) AND (review* OR overview* OR synthes*)) OR meta-analys* OR metaanalys*) |
| S8 | LA(English OR French OR Italian OR Spanish OR German) |
| S9 | (S1 OR (S2 AND S3)) AND (S4 OR (S5 AND (S6 OR S7))) AND S8 **limiters** publication date 20140101-20191231 506 results |

Web of Science 31 Mai 2019

| #1 | TS=( allerg* OR hypersensitiv* OR hyper-sensitiv* OR hyperresponsiv* OR hyper-responsiv*) |
| --- | --- |
| #2 | TS=(additive OR additives OR almond* OR apple OR apples OR apricot* OR arachis hypogaea OR avocado* OR banana* OR barley OR beef OR berries OR berry OR blackberr* OR blueberr* OR buckwheat* OR cacao OR calamari OR carrot* OR cashew* OR celery OR cherries OR cherry OR chestnut* OR chicory OR chocolat* OR cilandro OR clam OR clams OR cockle* OR coconut OR coconuts OR conch OR conchs OR coriander OR corn OR crab OR crabs OR cranberr* OR crawfish* OR crayfish* OR crustacea* OR cucumber* OR dairy OR dairies OR edamame* OR egg OR eggplant* OR eggs OR fig OR figs OR fish OR fishes OR flaxseed* OR food OR foods OR fruit OR fruits OR grape OR grapes OR hazelnut* OR kiwi OR kiwis OR lactose OR legume* OR lobster OR lobsters OR mango* OR meat OR melon OR melons OR milk OR mollusc* OR multifood OR mushroom* OR mussel OR mussels OR mustard OR nut OR nuts OR oat OR oats OR octopus OR "oral allergy syndrome" OR orange* OR oyster OR oysters OR parsley OR pea OR peach* OR peanut* OR pear OR pears OR peas OR pecan* OR periwinkle* OR pineapple* OR pistachio* OR plum OR plums OR pork OR potato* OR prawn OR prawns OR raspberr* OR rice OR rye OR salmon OR scallop OR scallops OR seafood* OR sesame OR shellfish* OR shrimp OR shrimps OR snail OR snails OR soja OR sojabean* OR soy OR soya OR soyabean* OR soybean* OR squid OR strawberr* OR sunflower seed* OR tomato* OR vegetable* OR walnut* OR wheat OR wheats OR whelk OR whelks OR zucchini*) |
| #3 | TS=(Social* OR Psychosocial* OR Psycholog* OR Quality of Life OR Life quality OR QoL OR HRQoL OR burden*) |
| #4 | TS=(Epidemiolog* OR prevalence* OR Frequenc* OR occurrence* OR incidence* OR cost* OR expens* OR expenditure* OR economic* OR financ* OR budget* OR spending*) |
| #5 | TS=( Canad* OR Alberta* OR Calgary OR Edmonton OR British Columbi* OR Vancouver OR Manitoba* OR New Brunswick OR Newfoundland OR Labrador OR Northwest Territor* OR Nova Scotia* OR Nunavut OR Ontari* OR Toronto OR Ottawa OR Prince Edward Island OR Quebec* OR Montreal OR Saskatchewan OR Yukon) |
| #6 | TS=(((systematic* OR Umbrella) AND (review* OR overview* OR synthes*)) OR meta-analys* OR metaanalys*) |
| #7 | (#1 AND #2 AND ( #3 OR (#4 AND (#5 OR #6)))) AND LANGUAGE: (English OR French OR German OR Italian OR Spanish) Timespan=1998-2019 1438 results |

After the removal of duplicates, there were a total of 2894 records retrieved.

## Appendix 2B: Eligibility criteria for inclusion of clinical studies

|  | **Inclusion / exclusion criteria** |
| --- | --- |
| **INCLUSION** |  |
| **Patient population** | - Patients of any age with clinician-diagnosed IgE-mediated food-allergy (confirmed through positive skin prick test, specific IgE level and/or oral food challenge test) including eggs, peanuts, milk, wheat, soy, tree nuts, or other |
| **Intervention** | - Oral immunotherapy using food products (including patented food-based products)   *Note: Sublingual (SLIT) and epicutaneous (EPIT) immunotherapy was only to be discussed in terms of background information* |
| **Comparators** | - Routine care (allergen avoidance and prescription of epinephrine injection device), no treatment, placebo - OIT with different protocols |
| **Outcomes** | Efficacy:   - desensitization - continued consumption - sustained unresponsiveness - Impact of patient characteristics and protocol variables on efficacy outcomes   Adverse effects:   - Severe reactions (systemic reactions, anaphylaxis, serious adverse events, intra-muscular epinephrine use) - Any reactions (mild, local reactions) - Discontinuations due to adverse events - Long-term follow-up for safety and tolerability - Eosinophilic esophagitis - Impact of patient characteristics and protocol variables on safety outcomes   Impact on QoL:   - Changes in disease-specific and generic health-related quality of life measured using validated instruments   Patient perspectives on the intervention:   - Acceptability, burden of treatment, impact on autonomy, values, preferences, expectations and goals of treatment |
| **Study design** | Generally included:   - Meta-analyses published since 2015 - Randomized controlled clinical trials (RCTs) OIT vs placebo or avoidance with N (enrolled)> 50 not included in meta-analyses - Large (N>150) observational studies of OIT in clinical practice or and non-randomized, controlled, parallel group intervention studies (controlled, clinical trials, CCTs)   Studies not fulfilling the above criteria were included if they addressed the following data gaps or questions concerning patient selection or OIT protocol parameters:   - Long-term follow-up of controlled trials (with control arm) and observational studies (≥ 6 months after reaching maintenance) - Impact of patients’ age on the outcomes of OIT / studies in adults or adolescents - Impact of other patient characteristics on OIT outcomes - Multi-food OIT - Less common allergens (e.g., wheat, soy, walnut) - Baked food product protocols - Studies focusing on EoE and its impact on the outcomes of OIT - Patient-reported outcomes (PROs): quality of life, burden-of-treatment - Impact of different modes of communication and shared decision-making on clinical or PRO outcomes: - RCTs exploring OIT with adjuvant therapy (e.g., biologics, probiotics) - Studies comparing different OIT protocols |
| **EXCLUSION** |  |
| **Outcomes** | - Studies reporting immunological outcomes only (surrogate outcomes) (*Note: immunological outcomes were extracted if reported by otherwise included studies*) |
| **Design** | - Case reports |
| **Language** | - Language other than French or English |
| **Completeness of reporting** | - Conference papers (excluded due to incomplete reporting and lack of peer-review) |
| **Time of publication** | - Articles published before 1998 |

# Appendix 3: Detailed clinical evidence tables

## OIT PROTOCOL VARIABLES IN CLINICAL STUDIES OR PUBLISHED CLINICAL PRACTICE

|  | **Allergen food preparation** | **Single starting dose in initial dose escalation** | **Build-up phase starting dose (per day)** | **Target dose (per day)** | **Up-dosing interval during build-up phase** | **Length of build-up phase (months)** | **Maintenance phase** |
| --- | --- | --- | --- | --- | --- | --- | --- |
| **Peanut** |  |  |  |  |  |  |  |
| RCTs with N> 50 | **Peanut flour** (Anagnostou, 2014;^1^ Blumchen, 2019;^2^ Reier-Nilsen, 2019a^3^; Tang, 2015^4^), **AR101 powder** (Bird, 2018^5^; Vickery, 2018^6^)  Adjuvant: *Lactobacillus rhamnosus CGMCC 1.3724* (Tang, 2015^4^) | **0.1-0.5 mg PP** (Bird, 2018^5^; Tang, 2015^4^; Vickery, 2018^6^) | **No initial dose escalation:**  **1-5 mg** (Anagnostou, 2014;^1^ Reier-Nilsen, 2019a^3^)  **0.1-30 mg** **PP** depending on eliciting dose (Blumchen, 2019^2^)  **With initial dose escalation:**  **3 mg PP** (Vickery, 2018^6^)  **6 mg PP** (Bird, 2018^5^,  **25 mg PP** (Tang, 2015^4^) | **125** **or** **250** **mg** **PP** depending on eliciting dose (Blumchen, 2019^2^)  **300 mg PP** (Bird, 2018^5^; Vickery 2018^6^)  **800 mg PP** (Anagnostou, 2014^1^)  **2000 mg PP** (Tang, 2015^4^)  **5000 mg PP** (Reier-Nilsen, 2019^3^) | **2-3 weeks** | 6 (Anagnostou, 2014^1^)  4.6 to 7.8 (Bird, 2018^5^)  13 median (range 10-14) (Blumchen, 2019^2^)  11.5 to 18.0 (Reier-Nilsen, 2019^3^)  8 (Tang, 2015^4^)  ~6.4 (Vickery, 2018^6^) | **5000 mg PP** daily for 36 months (Reier-Nilsen, 2019a^3^)  **2000 mg PP** daily for 10 months (Tang, 2015^4^)  **300 mg PP** daily for 24 weeks (Vickery, 2018^6^)  **50-250 mg** **PP** daily; median (observed): 125 mg for 8±2 weeks (Blumchen, 2019^2^) |
| Large case series | **Peanut flour**; **peanuts** for doses ≥ 300 mg PP (Nachshon, 2018^7^)  **Peanut flour**; for higher doses **peanuts**, peanut butter, and other **processed** **peanut food products** (Wasserman, 2019^8^)  **Peanut flour,** Bamba^®^ (**peanut flour puffs**), **peanut butter powder compounded** at local pharmacies into capsules with inert filler (to be opened) (Soller, 2019^9^) | **0.1 mg** **PP** (Nachshon, 2018^7^)  **2.5 µg PP** (Wasserman, 2019^8^)  **0.1 mg PP** (Soller, 2019^9^) | **Median: 20 mg PP (IQR 7.5-100)** - single highest tolerated dose during initial dose escalation (Nachshon, 2018^7^)  **Individualized** (Wasserman, 2019^8^)  **No initial dose escalation:**  **10 to 12 mg PP** (Soller, 2019^9^)  **With initial dose escalation:** individualized (Soller, 2019^9^) | **3000** **mg** **PP or ≥ 300 mg** for patients with difficulty reaching 3000 mg PP (Nachshon, 2018^7^)  **3000 mg** **PP** (Wasserman, 2019^8^)  **300-320 PP** (Soller, 2019^9^) | **Monthly** (Nachshon, 2018^7^)  **1-2 weeks** (Wasserman, 2019^8^)  **2 weeks** (Soller, 2019^9^) | 8.7 median (IQR: 3.8 – 12.8) (Nachshon, 2018^7^)  NR-Individualized (Wasserman, 2019^8^)  3.7 to 5.1 (16-22 weeks) (Soller, 2019^9^) | **3000 or 1200 mg PP** **daily** for unlimited time (Nachshon, 2018^7^)  **2000 mg of PP** **once or twice a day for** a minimum of 3 years (Wasserman, 2019^8^)  **300-320 PP** (Soller, 2019^9^) |
| **Cow’s milk** |  |  |  |  |  |  |  |
| RCTs with N> 50 | **Milk**, diluted or whole (Longo, 2008;^10^ Martorell, 2011;^11^ Morisset, 2007;^12^ de Schryver, 2019^13^) | **0.5 mg CMP** (=0.017 mL milk) (Longo, 2008^10^)  **0.3 mg CMP** (=0.010 mL milk) (De Schryver, 2019^13^) | **≤ 20 mL** milk (individualized) (Longo, 2008^10^)  **4 mL** milk (=120 mg CMP) (Martorell, 2011^11^)  **1 mL** milk (=30 mg CMP) (Morisset, 2007^12^)  **2.5 mL** (=75 mg CMP) De Schryver, 2019^13^) | **4500 mg CMP** (150 mL milk) (Longo, 2008^10^)  **6400 mg CMP** (200 mL milk) (Martorell, 2011; De Schryver, 2019^13^)  **7500 mg CMP** (250 mL milk) and dairy products (Morisset, 2007^12^) | **2 days** (Longo, 2008^10^)  **1 week** (Martorell, 2011;^11^ Morisset, 2007;^12^) (De Schryver, 2019^13^) | **12** (Longo, 2008^10^)  **3.7** (Martorell, 2011^11^)  **1.4** (Morisset, 2007^12^) | **150 – 200 mL** milk daily plus dairy products ad libitum (Longo, 2008; ^10^ Martorell, 2011;^11^  **200 mL/d** for 1 month, afterwards **≥200 mL twice a week** plus dairy products ad libitum (De Schryver, 2019^13^) |
| Large case series | **Powdered formula**; 3% fat **milk** for doses ≥ 90 mg of CMP (Levy, 2014^14^)  **Milk** diluted in water (Kauppila, 2014^15^) | **0.3 mg CMP** (=0.010 mL milk) (Levy, 2014^14^) | **No initial dose escalation:**  **0.5 mg CMP** (=0.017 mL milk) (Kauppila, 2014^15^)  **With initial dose escalation:**  **Median 52.5 mg CMP** (=1.75 mL milk) (Levy, 2014^14^) | **7200 mg CMP** (240 mL milk) (Levy, 2014^14^)  **6400 mg CMP** (200 mL milk) (Kauppila, 2014^15^) | **Monthly** (Levy, 2014^14^)  **1-2 weeks** (Kauppila, 2014^15^) | 4-6 (Kaupilla, 2014^15^) | 4500 mg CMP daily (**150 mL milk**) plus ad libitum milk products for unlimited time (Elizur, 2016^16^)  6400 mg CMP daily (**200 mL milk**) for unlimited time (Kauppila, 2014^15^) |
| **Chicken’s egg** |  |  |  |  |  |  |  |
| RCTs with N> 50 | Dried egg white powder/ dehydrated egg white (Burks, 2012;^17^ Escudero, 2015^18^)  Powdered pasteurised egg (Fuentes-Aparicio, 2013^19^)  Pasteurized egg white (Martin-Munoz, 2019a^20^)  Hard-boiled egg (Morisset, 2007^12^) | 0.1 mg dried egg white powder (Burks, 2012^17^)  0.08 mg EWP (Escudero, 2015^18^)  1 mg powdered pasteurised egg (Fuentes-Aparicio, 2013^19^)  0.11 mg EWP (Martin-Munoz, 2019a^20^) | 3-50 mg dried egg white powder (individualized) (Burks, 2012^17^)  0.02 mg EWP (Escudero, 2015^18^)  30 mg powdered pasteurised egg (Fuentes-Aparicio, 2013^19^)  Individualized, based on last tolerated dose in initial dose escalation (Martin-Munoz, 2019a^20^)  1 g of hard-boiled egg yolk (Morisset, 2007^12^) | 2 g of egg-white powder (~1/3 egg) (Burks, 2012^17^)  2.808 g EWP (Escudero, 2015^18^)  10 g powdered pasteurised egg (~1 egg) (Fuentes-Aparicio, 2013^19^)  30 mL pasteurized egg white (3.3 g EWP, ~1 egg) (Martin-Munoz, 2019a^20^)  4 g of egg yolk and 4 g of egg white plus foods containing eggs | **1-2 weeks**  **Weekly plus daily or weekly only** (Martin-Munoz, 2019a) | 10 (Burks, 2012^17^)  3 (planned), 1 (observed) (Escudero, 2015^18^)  3 (range 1.2-8.2) (Fuentes-Aparicio, 2013^19^)  2-3 (Morisset, 2007^12^) | 2 g of egg-white powder (~1/3 egg) daily for 12 months (Burks, 2012^17^)  ≥ 1 undercooked egg every 2 days plus any other foods containing egg ad libitum (Escudero, 2015^18^)  Normal, egg-containing diet (Fuentes-Aparicio, 2013^19^)  30 mL pasteurized egg white (3.3 g EWP, ~1 egg) daily or every 2 days (Martin-Munoz, 2019b^21^) |
| **Multi-food OIT** |  |  |  |  |  |  |  |
| Single-arm clinical study, Begin, 2014a^22^ (N=40) | Food flours/powders (milk powder; egg powder; peanut, walnut, cashew, almond, pecan, hazelnut, pecan, wheat, soy, and sesame seed flours) | 0.1 mg of each food allergen protein (i.e., 0.5 mg total food allergen protein for a patient with 5 allergies treated) | Highest tolerated dose at initial dose escalation (≤6 mg total food allergen protein) divided evenly among the treated food allergens | 4000 mg food allergen protein per allergen (up to 20,000 mg for those with 5 allergies) | 2 weeks | Individualized; median time to reach 1000 mg dose: approx. 16 months; 4000 mg:>20 months | 4000 mg food allergen protein per allergen (i.e., 20,000 mg total food allergen protein for a patient with 5 allergies treated) |
| Single-arm clinical study, Begin, 2014b^23^ (with omalizumab, N=25) |  | 5 mg total food allergen protein divided evenly among the treated food allergens (e.g., 1 mg of each allergen for a patient with 5 allergies treated) | Highest tolerated dose at initial dose escalation (≤1250 mg total food allergen protein) divided evenly among the treated food allergens | 4000 mg food allergen protein per allergen (up to 20,000 mg for those with 5 allergies) | 2 weeks | Individualized; median time to reach maintenance dose was 4.14 months (range 1.6–8.3) | 4000 mg food allergen protein per allergen |

CMP: cow’s milk protein; EWP: egg white protein; PP: peanut protein

Note: 1 peanut corresponds to approximately 250 mg peanut protein; 1 mL milk corresponds to 30 mg milk protein; 1 egg corresponds to 4g egg white protein or 30 mL pasteurized egg white or 6 g of egg-white powder

**Summary table**

| **Allergen food preparation** | **Adjuvants** | **Single starting dose in initial dose escalation** | **Build-up phase starting dose (per day)** | **Target dose (per day)** | **Up-dosing interval during build-up phase** | **Length of build-up phase (months)** | **Maintenance dose** |
| --- | --- | --- | --- | --- | --- | --- | --- |
| **Peanut**: Peanut flour, AR101 powder, peanuts, peanut butter and other processed peanut products (e.g., peanut flour puffs), compounded peanut butter powder  **Milk**: milk, diluted or whole; powdered (formula)  **Egg**: Dried / dehydrated egg white powder; pasteurized egg white; hard-boiled egg  **Multi-food OIT**: Food flours/powders | Lactobacillus, omalizumab | **Peanut:** 0.0025-0.5 mg peanut protein  **Milk**: 0.3-0.5 mg milk protein  **Egg**: 0.1-1 mg egg white powder or egg protein  **Multi-food OIT**: 0.1 mg of each food allergen protein | **Peanut:**  No initial dose escalation:  0.1-30 mg peanut protein  With initial dose escalation:  3-100 mg peanut protein  **Milk**:  No initial dose escalation:  0.5 mg milk protein  With initial dose escalation:  30-120 mg milk protein  **Egg**: 0.02-50 mg egg white powder or egg protein | **Peanut:** 800 -5000 mg peanut protein (low dose OIT: 125-320 mg)  **Milk**: 150-250 mL milk (4500-7200 mg milk protein)  **Egg**: ~1 egg or ~1/3 egg  **Multi-food OIT**: 4000 mg food allergen protein per allergen (up to 20,000 mg for those with 5 allergies) | Most common: 2-3 weeks; range: 2 days to 1 month | **Peanut**: 3.7 to 18  **Milk**: 1.4 to 12  **Egg**: 3 to 10  **Multi-food OIT:**  No omalizumab median > 20  With omalizumab: 1.6-8.3, median 4.14 | **Peanut:** 1200-5000 mg peanut protein /d (low dose OIT: 50-320 mg/d)  **Milk:** 150-200 mL daily or at least twice weekly (4500-6500 cow milk protein)  **Egg**: 3.3 g egg white protein (~1 egg) or 2 g of egg-white powder (~1/3 egg) or ≥ 1 undercooked egg daily or every 2 days |

**Protocols for other food allergens**

|  | **Nowak-Wegrzyn, 2019^24^ (RCT, N=46) -wheat** | **Kulmala, 2018^25^ (prospective, single-arm study, N=100) -wheat** | **Elizur, 2019^26^ (CCT, N=73)-walnut** | **Barni, 2019^27^ (case series, N=43) - hazelnut** | **Nachshon, 2019^28^ (CCT) N=75 - sesame** |
| --- | --- | --- | --- | --- | --- |
| **Age** | Median, 8.7 years; range, 4.2-22.3 | Mean 11.6 years, range, 6.1-18.6 | Mean 7.9 years OIT, 6.8 years control; range 4-20 | Mean 10 years, range: 5-16 | Mean 7.5 years, ≥4, (IQR: 5.8-11.6) |
| **Diagnosis** | DBPCFC | Open OFC (not performed in 15 patients with an immediate reaction within the previous 3 months) | OFC, unless an immediate, recent (past year) reaction was documented | OFC | OFC or reaction within past year together with positive skin prick test (SPT) result and/or specific serum IgE (>0.35 kUA/L) |
| **OIT protocol** | Biweekly escalation for up to 44 weeks to a maximum of 1445 mg of wheat protein (WP), followed by daily home maintenance dosing. After 52 weeks of treatment (≥ 8 weeks of maintenance dosing), DBPCFC for a cumulative dose of 7443 of WP (2-3 slices of bread). Subjects continued OIT for another year and underwent a year 2 DBPCFC and, if passed, a subsequent off therapy DBPCFC. Placebo-treated subjects crossed over to high-dose OIT (maximum, 2748 mg of WP). | Well-cooked wheat spaghetti was consumed every day for 17 weeks, increasing every 1-2 weeks from 0.3 to 2000 mg of wheat protein, followed by 3-month maintenance and 9-month follow-up  Patients received antihistamine every day | Initial 4-day dose-escalation phase to establish the single highest tolerated dose, which was consumed daily at home for 24 days; subsequent monthly dose escalations were repeated until 4000 mg walnut protein was achieved. Patients who were desensitized to walnut continued to consume 1200 mg walnut protein daily for 6 months as maintenance. | Initial hospital OFC followed by hazelnut intake at home 3 times/ week with a dose equal to the maximum dose tolerated during hospital stay, up-dosing every 3 months at the hospital until 2.5 g hazelnut (cumulative dose) was reached | The initial 2 days of first dose-escalation round served as an entry OFC up to 4800 mg sesame protein (non-reacting patients were excluded).  The single highest tolerated dose (SHTD) was then consumed at least once daily at home for 24 days. ubsequently, patients returned for monthly dose escalations performed in an ambulatory day care setting,  Patients who could tolerate the target dose of 4000 mg sesame protein at the end build-up, were considered fully desensitized and were instructed to consume a daily maintenance dose of 1200 mg sesame protein (5g Tahini) plus ad-lib consumption of sesame products  Patients who tolerated ≥ 240 mg sesame protein (~1 g of Tahini) but<4000 mg were considered partially desensitized and instructed to consume their achieved dose for an unlimited time |

## CLINICAL EFFICACY

### Desensitization: increase in reactivity threshold

#### Data for peanut, milk and egg allergies

| **Meta-analyses** | | | **RCT not included in meta-analyses with N>50** | |
| --- | --- | --- | --- | --- |
| **Nurmatov, 2017^29^ – chicken’s egg, cow’s milk, peanut and others** | **Romantsik, 2018^30^ – chicken’s egg** | **Chu, 2019^31^ – peanut** | **Martin-Munoz, 2019a^20^ (Spain) – chicken’s egg**  **N=101**  **Age: 6-9 years**  **Diagnosis confirmed with OFC: yes** | **De Schryver, 2019^13^ (Canada) – cow’s milk**  **N=52**  **Age: 6-18, mean 12.1 years**  **Diagnosis confirmed with OFC: yes (single-blind)** |
| Desensitization in patients assessed in DBPCFC:  All OIT trials - 17 RCTs^1,10-12,18,19,32-42^ and 5 CCTs^43-47^  **OIT: 75% (432/573)**  **Control 11% (44/409)**  RR (Control/OIT) = 0.14 (95% CI 0.08, 0.24)  OIT RCTs only:^1,10-12,18,19,32-42^  OIT: 71% (291/410)  Control: 13% (43/334)  RR (Control/OIT) = 0.18 (95% CI 0.10, 0.32) **- 5.55**  Diagnosis confirmed by DBPCFC:  OIT: 73% (412/565)  Control: 13% (43/334)  RR (Control/OIT) = 0.15 (95% CI 0.09, 0.27)  **P<0.0001**  Chicken’s egg: - 9 RCTs^12,18,19,32,34,35,37,39,48^ (1 SLIT^48^) and 1 CCT^47^  **OIT: 74% (251/339)**  **Control: 17% (36/208**), P<.0001  RR (Control/OIT) = 0.22 (95% CI 0.11, 0.45)  Cow’s milk - 9 RCTs^10,11,33,36,38-41,48^ (1 SLIT^48^) and 4 CCTs^43,44,46,47^ – tolerance to median 45 to 250 mL (=1340-7500 mg milk protein) milk (median 200 mL=6000 mg) across trials  **OIT: 78% (262/336)**  **Control: 7.8% (15/193),** P<.0001  RR (Control/OIT) = 0.12 (95% CI 0.06, 0.25)  Peanut - 2 OIT^1,42^ and 2 SLIT^49,50^ RCTs  **OIT: 73% (65/89)**  **Control: 3.7% (3/82),** P<.0001  RR (Control/OIT) = 0.11 (95% CI 0.04, 0.31) | Induction of immunologic tolerance and/or ability to tolerate a full serving of egg as assessed in 9 RCTs^18,19,32,34,35,37,51-53^ and 1 CCT:^54^  **OIT: 45% (112/249)**  **Control: 10% (19/190)**  RR: 4.25 (95% CI 2.77, 6.53)  *GRADE assessment: low quality of evidence*  Ingestion of a partial serving of egg (1 g to 7.5 g) as assessed in 8 RCTs^18,19,32,34,35,37,51,53^ and 1 CCT:^54^  **OIT: 82% (192/234)**  **Control: 10% (18/176)**  RR= 7.48 (95% CI 4.91, 11.38)  *GRADE assessment: low quality of evidence* | Passing an OFC at the highest dose tested (8 RCTs^1,2,4-6,42,55^, 1 unpublished):   - *Original analysis:* median cumulative dose 4250 mg PP (range 1043-5000 mg) = 17 (4.2-20) peanuts   **OIT: 56% (320/574)**  **Control: 3.2% (9/284)**  RR=12.42 (95% CI 6.82, 22.61), P<.001  *GRADE assessment: High quality of evidence*   - *Corrected analysis:* median cumulative dose 3022 mg PP (range 1043-5000 mg) = 12 (4.2-20) peanuts   **OIT: 55% (315/574)**  **Control: 2.8% (8/284)**  **– see Section 8.1**  *Alternative analysis: achieving at least partial desensitization:* passing an OFC with a cumulative dose of 400 to 5000 mg PP (1.6-20 peanuts) (median 2700 mg) across 8 RCTs^1,2,4-6,42,55^ (1 unpublished):  ***OIT: 73% (422/574)***  ***Control: 7.0% (21/284)*** | Passing DBPCFC at 12 months with 3.3 g pasteurized raw egg white protein (cumulative) or reaching target dose of 3.3 g pasteurized raw egg white egg white protein (equivalent to 1 egg):  **OIT: 84% (64/76)**  **Control: 16% (4/25)**  **P=0.000**  *Cochrane risk of bias: high (MW assessment)* | OIT patients: reaching maintenance dose (200 mL) (8.8 g milk protein)  Control patients: passing a single-blind OFC with a cumulative dose of 200 mL milk (8.8 g milk protein):  **OIT: 69% (18/26)**  **Control: 0 (0/26)**  **Difference: 69% (95% CI: 48%, 91%)** |

DBPCFC: double-blind, placebo-controlled food challenge; IQR: inter-quartile range; OFC: oral food challenge; OIT: oral immunotherapy; PP: peanut protein; RR: risk ratio; SLIT: sublingual immunotherapy

Note: approximate correspondence: 1 peanut = 250 mg peanut protein; 1 mL milk = 30 mg milk protein; 1 egg white=4g egg white protein

*An additional 15 patients continued to receive increasing amounts of cow milk at date of data cut-off (i.e., had not completed the build-up phase).

| **Case series (clinical practice) with N>150** | | | | |
| --- | --- | --- | --- | --- |
| **Levy, 2014^14^ (Israel)– cow’s milk**  **N=265***  **Age: 4-27 (7.5 median) years**  **Diagnosis confirmed with OFC: not for all patients** | **Kauppila, 2019^15^ (Finland) – cow’s milk**  **N=295**  **Age: 5-17 (median 7.5) years**  **Diagnosis confirmed with OFC: yes** | **Soller, 2019^9^ (Canada) – peanut**  **N=270**  **Age: 0.75-5.9 (median 1.9, IQR : 1.25-2.75) years**  **Diagnosis confirmed with OFC: for 31% of patients** | **Wasserman, 2019^8^ (USA) – peanut**  **N=270**  **Age: 4-18 (mean: 8.1) years**  **Diagnosis confirmed with OFC: no** | **Wasserman, 2014^56^ (USA and Israel)- peanut**  **N=352**  **Age: 3-24 years**  **Diagnosis confirmed with OFC: 97% of patients** |
| Completion of treatment program and ability to freely consume milk or milk products (> 7.2 g milk protein): **60%** (160/265)  Ability to tolerate ≥ 180 mg of milk protein (6 mL of milk): **85%** (226/265) | Consuming ≥200 mL/d of milk (6.4 g milk protein) 3 months after reaching the maintenance dose of 200 mL: **71%** (209/295);  Consuming <200 mL (10-190 mL): 18% (54/295)  *Consuming ≥ 10 mL milk: 89% (263/295)*  Avoiding milk consumption: or lost to follow-up: 11% (32/295) | Reaching the target maintenance dose of 300-320 mg PP (1.2-1.3 peanuts): **90%** (243/270) | Reaching the target dose of 3000 mg PP: **78%** (211/270) (an additional 3 patients reached a planned target dose of 2000 mg PP)  Passing an OFC with 6000 mg PP: **78%** (210/270) | Reaching the target dose of 415-8000 mg PP: **85%** (298/352) |

DBPCFC: double-blind, placebo-controlled food challenge; IQR: inter-quartile range; OFC: oral food challenge; OIT: oral immunotherapy; PP: peanut protein; RR: risk ratio

Note: approximate correspondence: 1 peanut = 250 mg peanut protein; 1 mL milk = 30 mg milk protein; 1 egg white=4g egg white protein

*An additional 15 patients continued to receive increasing amounts of cow milk at date of data cut-off (i.e., had not completed the build-up phase).

#### Data for other food allergies

|  | **Nowak-Wegrzyn, 2019^24^ (RCT, N=46) -wheat** | **Kulmala, 2018^25^ (prospective, single-arm study, N=100) -wheat** | **Elizur, 2019^26^ (CCT, N=73)-walnut** | **Barni, 2019^27^ (case series, N=43) - hazelnut** | **Nachshon, 2019^28^ (CCT) N=75 - sesame** |
| --- | --- | --- | --- | --- | --- |
| **Age** | Median, 8.7 years; range, 4.2-22.3 | Mean 11.6 years, range, 6.1-18.6 | Mean 7.9 years OIT, 6.8 years control; range 4-20 | Mean 10 years, range: 5-16 | Mean 7.5 years, ≥4, (IQR: 5.8-11.6) |
| **Diagnosis** | DBPCFC | Open OFC (not performed in 15 patients with an immediate reaction within the previous 3 months) | OFC, unless an immediate, recent (past year) reaction was documented | OFC | OFC or reaction within past year together with positive skin prick test (SPT) result and/or specific serum IgE (>0.35 kUA/L) |
| **Desensitization** | At year 1, 12/23 (52%) low-dose VWG OIT-treated and none of 23 placebo-treated subjects consumed successfully ≥ 4443 mg of WP (primary end point) (P < .0001); median doses successfully consumed were 4443 and 143 mg, respectively.  Among placebo-treated subjects who crossed over to high-dose VWG OIT, 12/21 (57%) were desensitized after 1 year (median dose, 7443 mg of WP; nonsignificant vs low-dose VWG OIT). | At the end of the 17-week build-up period, 64% (64/100) patients reached the target dose of 2000 mg /d WP (24 strands of spaghetti). Among the remaining 36 patients (including 23 who had discontinued), the median maximum tolerated dose was 5.5 strands of spaghetti (445 mg WP, range 1–1760)  After 3 month maintenance, 47 patients consumed 2000 mg/d WP, 25 consumed a median of 330 mg (range 5–1750) and 5 had discontinued | Passing an OFC with 4000 mg of walnut protein (26-g walnut): 49/55 (89%) patients in the OIT group compared to 0/18 patients in the control group (odds ratio 9·2, 95% CI 4·3–19·5; P<.0001).  Nine (50%) control patients began walnut OIT, and 7 (78%) were desensitized to 4000 mg walnut protein  All patients who were co-allergic to pecan (n=46) were also desensitized to pecan. Additionally, 18/30 (60%) patients who were co-allergic to hazelnut or cashew, and 14/15 (93%) patients who were co-allergic to hazelnut alone, were either fully desensitized or responded to treatment. | 65% (28/43) reached the cumulative dose of 2.5 g hazelnut (0.375 g hazelnut protein)  35% (15/43) ongoing, tolerating a mean dose of 255 mg hazelnut  Average time to complete treatment was 5 months (range 3-12) | Full desentiziation (4000 mg sesame protein): 88% (53/60) vs 0% (0/15) (median time to achieving maintenance dose was 6.5 (IQR, 3.8-12.8) months)  At least partial desentiziation (≥ 240 mg sesame protein): 100% (60/60) vs 0% (0/15)  Control (eligible, but did not undergo OIT due to non-clinical reasons, eg., long distance from clinic): 0% (0/15) |

DBPCFC: double-blind, placebo-controlled food challenge; SCD: successfully consumed dose; VWG: vital wheat gluten

*Note: 7 walnuts correspond to approx. 28.3 g (1 ounce serving); 12 hazelnuts correspond to approx. 28.3 g (1 ounce serving), with 1 hazelnut weighting about 2.4 g*

#### Assessment of the quality of evidence

|  |  | **Risk of bias** (Cochrane tool^57^ for interventional comparative studies; IHE tool^58^ for case series) | **Level of evidence (Oxford Centre for Evidence-based medicine) -** adapted using the approach of the European Academy of Allergy and Clinical Immunology^59^ |
| --- | --- | --- | --- |
| **Peanut** | **Interventional comparative studies (RCTs, CCTs):** Chu meta-analysis (8 RCTs^1,2,4-6,42,55^, 1 unpublished) | **Low -**  8 RCTs of which 5 were rated as being at low risk of bias and 3 as high risk of bias for this outcome* | Level I (Systematic reviews, meta-analysis, randomized controlled trials) |
|  | **Case series:** Soller, 2019^9^, Wasserman, 2019,^8^ Wasserman, 2014^56^ | **Moderate** – 3 case series: 2 with retrospective study design; 1 single center; OFC as outcome in 1 study only |  |
| **Egg** | **Interventional comparative studies (RCTs, CCTs):** Romantsik, 2018^30^ meta-analysis (9 RCTs^18,19,32,34,35,37,51-53^ and 1 CCT^54^) plus 1 RCT (Martin-Munoz, 2019a^20^) | **High** – All RCTs rated as being at overall high risk of bias by ≥1 analysis | Level I (Systematic reviews, meta-analysis, randomized controlled trials) |
|  | **Case series** | **--** |  |
| **Milk** | **Interventional comparative studies (RCTs, CCTs):** meta-analysis (9 RCTs^10,11,33,36,38-41,48^ (1 SLIT^48^) and 4 CCTs^43,44,46,47^) plus 1 RCT (De Schryver, 2019^13^) | **High** – meta-analysis: of the 9 RCTs, 5 were rated as being at high risk of bias and 2 at unclear risk of bias; RCT not in meta-analysis- high risk of bias | Level I (Systematic reviews, meta-analysis, randomized controlled trials) |
|  | **Case series**: Levy, 2014^14^ and Kauppila, 2019^15^ (clinical practice) | **Moderate** – 2 case series from clinical practice, main limit: retrospective study design, no OFC as outcome; 1 was conducted at a single center |  |
| **Wheat** | **Interventional comparative studies (RCTs, CCTs)**: Nowak-Wegrzyn, 2019^24^ | **Low** – 1 RCT | Level I (Systematic reviews, meta-analysis, randomized controlled trials) |
|  | **Case series**: 1 prospective case series in research context (Kulmala, 2018^25^) | **Low** – 1 prospective case series |  |
| **Walnut** | **Interventional comparative studies (RCTs, CCTs)**: Elizur, 2019^26^ (CCT, N=73) | **High** – non-randomized control group,** unblinded | Level II two groups, non-randomized studies |
|  | **Case series**: NA |  |  |
| **Hazelnut** | **Interventional comparative studies (RCTs, CCTs)**:NA | **--** | Level III: one group non-randomized |
|  | **Case series:** Barni, 2019^27^ (clinical practice) | **High** - 1 case series from clinical practice, limits: retrospective study design, single center, unclear recrutement and eligibility criteria |  |
| **Sesame** | **Interventional comparative studies (RCTs, CCTs)**: Nachshon, 2019^28^ | **High** – non-randomized control group,*** unblinded | Level II two groups, non-randomized studies |
|  | **Case series**: NA | **--** |  |

*Note: Sensitivity analyses that adjusted for risk of bias yielded similar results to the main analysis (Chu et al, 2019^31^).

** Treatment assignment was based on the order in which patients presented at to the clinic, with the first patients starting OIT and the remain ing patients being managed by observation and strict dietary exclusion (control group).

*** Controls were patients eligible for OIT but refrained from treatment during the study period for nonmedical reasons, such as a large travel distance to the clinic or previous obligations at the time of their invite to treatment.

### Continued allergen food consumption

#### Data for peanut, milk and egg allergies

| **LTFU of RCTs (with control arm data)** | | | **Large case series (N≥145)** | | |
| --- | --- | --- | --- | --- | --- |
| **Jones, 2016^60^ – LTFU of Burks, 2012 (egg)^32^**  Diagnosis confirmed with OFC: no | **Hsiao, 2017^61^ – LTFU of Tang 2015 (peanut):^4^**  Diagnosis confirmed with OFC: no | **Meglio, 2017^62^ – LTFU of Meglio 2013 (egg)^37^**  Diagnosis confirmed with OFC (DBPCFC) | **Kauppila, 2019^15^ (Finland) – cow’s milk**  N=295  Age: 5-17 (median 7.5) years  Diagnosis confirmed with OFC | **Elizur, 2016^16^ (Israel) - cow’s milk**  N=196  Age: >6, mean 10 years  Diagnosis confirmed with OFC for patients without an anaphylactic reaction in the preceding year | **Nachshon, 2018^7^ (Israel)- peanut***  N=145  Age: ≥4 (median 5.8) years  Diagnosis confirmed with OFC for patients without an anaphylactic reaction in the preceding year |
| **Patients followed:** All patients who started OIT or control therapy  **Follow-up**: Point 1: approx. 5 years after study enrolment; Point 2: 1 year later  **Completeness of follow-up**:  OIT: 85% (34/40); placebo: 73% (11/15) at both time points  Among patients with follow-up:  **Eating baked or unbaked egg:** At point 1: OIT: 82% (28/34) vs placebo: 36% (4/11), P=.007; At point 2: OIT: 85% (28/33) vs placebo: 67% (8/12), P=.22  **Eating baked and unbaked egg:** At point 1: OIT: 68% (23/34) vs placebo: 18% (2/11), P=.006; At point 2: OIT: 64% (21/33) vs placebo: 25% (3/12), P=.04  *Sensitivity analysis (all lost to FU assumed to avoid):*  *At point 1:*  *OIT: 58% (23/40) vs placebo: 13% (2/15);*  *At point 2: OIT: 53% (21/40) vs placebo: 20% (3/15)*  20 OIT patients had demonstrated SU at some point over 4 years of treatment; 18 of them responded to the FU survey and all (100%) reported consumption of all forms of egg at points 1 and 2. | **Patients followed:** All patients who started OIT or control therapy  **Follow-up:** Mean of 4.2 years (SD 0. 7) from treatment cessation  **Completeness of follow-up**: 77% (OIT: 24/31; placebo: 24/31) of patients    Among patients with follow-up:  **Eating peanuts:**  OIT: 67% (16/24) vs Control 4.2% (1/24) P=.001  *Sensitivity analysis (all lost to FU assumed to avoid):*  *OIT: 52% (16/31) vs Control 3.2% (1/31)*  **Eating peanuts at least weekly:**  OIT: 46% (11/24) vs Control 4.2% (1/24)  P=NR  *Sensitivity analysis (all lost to FU assumed to avoid):*  *OIT: 35% (11/31) vs Control 3.2% (1/31)*  **Eating > 2000 mg PP:**  OIT: 52% (12/23)  Placebo: 4% (1/24)  P=.001  23 OIT patients had demonstrated SU at 18 months of treatment; 20 of them responded to the FU survey. 16/20 (80%) reported regular consumption of peanuts at FU, 1 had discontinued due to taste aversion and 3 failed subsequent DBPCF | **Patients followed:** All patients who started OIT or control therapy  **Follow-up:** Mean of 2.5 (SD 0.3) and 7 (SD 0.9) years after original study start  **Completeness of follow-up**: 2.5 years: 9/10 OIT, 10/10 control; 7 years: 9/10 OIT, 9/10 control  Among patients with follow-up:  **Being able to eat raw and/or cooked egg at least once a week without symptoms:**  OIT group: 7/9 children (78%) at both time points  Control: 2.5-year FU: 3/10 (30%), P<.05; 7-year FU: 3/9 (33%), P=n.s. (2 control children started OIT)  *Sensitivity analysis (all lost to FU assumed to avoid):*  *OIT: 70% (7/10) vs Control 30% (3/10)* | **Patients followed:** All patients who started OIT  **Follow-up**: median 6.5 years (range 1-11)  **Completeness of follow-up**:  83% (244/295) of patients  Among patients with follow-up:  **Consuming ≥200 mL milk/d:** 56% (136/244) – *SA: 46% (136/295)*  **Consuming ≥200 mL milk/d with no self-reported milk-related side effects in the last year:** 26% (77/244)  **Consuming <200 mL (10-190 mL) milk/d:** 18% (44/244)  **Avoiding milk consumption**: 26% (65/244) of patients  **Any milk consumption** (≥ 10 mL/d)**:** 74% (180/244)  *Sensitivity analysis (all lost to FU assumed to avoid): 61% (180/295)* | **Patients followed:** Patients who reached full cow milk protein consumption  **Follow-up**: ≥ 6 months after completing build-up phase; median: 24.8 (range 6–41) months  **Completeness of follow-up**:  195/196 (99%) of patients  Among patients with follow-up:  **Consuming ≥ 4.5 g of cow milk protein ≥3 times per week**:  93% (181/195) of patients  *Sensitivity analysis (all lost to FU assumed to avoid): 92% (181/196)*  **Discontinued milk consumption**: 7.2% (14/195) of patients | **Patients followed:** All patients who started OIT  **Follow-up:** ≥ 6 months after completing build-up phase; median: 18 (range 6–75) months  **Completeness of follow-up**:  142/145 (98%) of all patients  130/133 (98%) of patients reaching 3000 mg (n=113) or 300-2400 mg (n=20) PP at end of up-dosing  **Eating peanuts regularly (≥300 mg peanut protein/d)**:  All patients with follow-up: 113/142 patients (80%)  *Sensitivity analysis (all lost to FU assumed to avoid): 78% (113/145)*  Patients who reached maintenance: 87% (113/130)  *Sensitivity analysis (all lost to FU assumed to avoid): 85% (113/133)*    **Discontinued eating peanuts** (among patients who reached maintenance): 17/130 (13%)  **Reasons for discontinuation**: food aversion (13/17), treatment burden (3/17), heartburn (1/17)  **Failed OIT (did not reach maintenance dose):** 12/142 (8.5%) |

PP: peanut protein

#### Data for other food allergies

|  | **Nowak-Wegrzyn, 2019^24^ (RCT, N=46) -wheat** | **Kulmala, 2018^25^ (prospective, single-arm study, N=100) -wheat** | **Elizur, 2019^26^ (CCT, N=73)-walnut** | **Barni, 2019^27^ (case series, N=43) - hazelnut** | **Nachshon, 2019^28^ (CCT) N=75 - sesame** |
| --- | --- | --- | --- | --- | --- |
| **Age** | Median, 8.7 years; range, 4.2-22.3 | Mean 11.6 years, range, 6.1-18.6 | Mean 7.9 years OIT, 6.8 years control; range 4-20 | Mean 10 years, range: 5-16 | Mean 7.5 years, ≥4, (IQR: 5.8-11.6) |
| **Diagnosis** | DBPCFC | Open OFC (not performed in 15 patients with an immediate reaction within the previous 3 months) | OFC, unless an immediate, recent (past year) reaction was documented | OFC | OFC or reaction within past year together with positive skin prick test (SPT) result and/or specific serum IgE (>0.35 kUA/L) |
| **Long-term follow-up** | No data | After 9 month maintenance:  39/100 consumed ≥2000 mg/d WP  18/100 consumed a median of 500 mg (range 83–1000) WP (1-12 strands of spaghetti)  43/100 had discontinued | 45 of 56 patients who had been desensitized to walnut had follow-up data (≥ 6 months after end of OIT) and were consuming a daily dose of 1200 mg walnut protein – 70% (45/64) of patients who had initiated OIT | At 1-year follow-up, all patients who had completed treatment continued to consume hazelnut, at a dose of ≤ 2.5 g ≥ 1 time a week, without adverse reactions | Follow-up ≥6 months after reaching the maintenance dose (median 8, range 5.5-34.6) months  Completeness of FU: 100% (56/56-4 patients had not yet reached ≥6 months) for regular consumption, 82% (46/56) for OFC  Regular consumption (ITT analysis):  ≥1200 mg sesame protein : 88% (49/56)  ≥480 mg sesame protein : 93% (52/56)  OFC with 4000 mg sesame protein: 82% (46/56) |

#### Assessment of the quality of evidence

|  |  | **Risk of bias** (Cochrane tool^57^ for interventional comparative studies; IHE tool^58^ for case series) | **Level of evidence (Oxford Centre for Evidence-based medicine) -** adapted using the approach of the European Academy of Allergy and Clinical Immunology^59^ |
| --- | --- | --- | --- |
| **Peanut** | **Interventional comparative studies (RCTs, CCTs)** - *Hsiao, 2017^61^* | **High**- 1 LTFU on RCT, limitations: open-label and loss to follow-up | Level II two groups, non-randomized studies |
|  | **Case series -** *Nachshon, 2018^7^* | **Moderate –** 1 case series; main limits: single-center, retrospective data collection |  |
| **Egg** | **Interventional comparative studies (RCTs, CCTs) -** *Jones 2016^60^, Meglio 2017^62^* | **High** - 2 LTFU on RCTs, limitations: open-label and loss to follow-up | Level II two groups, non-randomized studies |
|  | **Case series** | **--** |  |
| **Milk** | **Interventional comparative studies (RCTs, CCTs):** | **--** | Level III: one group non-randomized |
|  | **Case series**: **-** *Kauppila 2019^15^, Elizur 2016^16^* | **Moderate** – 2 large case series; main limits: single-center, retrospective data collection |  |
| **Wheat** | **Interventional comparative studies (RCTs, CCTs)** | **--** | Level III: one group non-randomized |
|  | **Case series**: 1 prospective case series in research context (Kulmala, 2018^25^) | **Low** – 1 prospective case series of low risk of bias |  |
| **Walnut** | **Interventional comparative studies (RCTs, CCTs)**: Elizur, 2019^26^ (CCT, N=73) | **High** – non-randomized control group,** unblinded | Level II two groups, non-randomized studies |
|  | **Case series**: NA |  |  |
| **Hazelnut** | **Interventional comparative studies (RCTs, CCTs)**:NA | **--** | Level III: one group non-randomized |
|  | **Case series:** Barni, 2019^27^ (clinical practice) | **High -** 1 small case series from clinical practice, limits: retrospective study design, single center, unclear recruitment and eligibility criteria and follow-up |  |
| **Sesame** | **Interventional comparative studies (RCTs, CCTs)**: Nachshon, 2019^28^ | **High** – single-arm follow-up, unblinded | Level III: one group non-randomized |

### Sustained unresponsiveness

#### Data for peanut, milk, egg allergies

| **Meta-analyses : Nurmatov, 2017^29^** |
| --- |
| Ability to safely consume foods containing the allergen in question after discontinuing as assessed in 3 egg RCTs (Burks 2012,^32^ Caminiti, 2015,^34^ Escudero, 2015,^18^) and 1 milk/egg RCT (Staden, 2007^41^) with DBPCFC - approx. 88% of patients were treated for egg allergy, period of avoidance was 1-3 months across studies  **OIT: 34%** (36/113)  **Control: 11%** (9/81)  RR (Control/OIT)=0.29 (95% CI 0.08, 1.13)  P < 0.074 |

DBPCFC: double-blind controlled food challenge

**RCTs (N≥50) not included in meta-analyses**

| **Study** | **Age (years)** | **Design** | **Intervention (N initiated treatment)** | **Comparator (N initiated treatment)** | **Allergy confirmed with DBPCFC?** | **Interval between treatment initiation and SU assessment** | **Intervening period of allergen avoidance** | **SU assessment** | **SU – ITT analysis** |
| --- | --- | --- | --- | --- | --- | --- | --- | --- | --- |
| Sampson, 2019 [abstract],^63^ USA – **egg*** | Mean 8 (range 3.5–16.8) | RCT | OIT (N=23) | Baked egg products consumption (N=27) | Yes | 24 months (2 years) | 8-10 weeks | DBPCFC - 7.4 g egg-white protein (1.9 egg whites), cumulative | 44% (10/23) vs 11% (3/27), P=.009 |
| Tang, 2015,^4^ (*OIT with probiotic Lactobacillus*) Australia – **peanut** | Mean 6 (SD 2.4) | Double-blind, placebo-controlled RCT | OIT + probiotic (Lactobacillus rhamnosus CGMCC 1.3724) (N=31) | Placebo (N=31) | For a subset of participants | 18 months | 2-5 weeks | DBPCFC -2 g peanut protein (8 peanuts), cumulative | 74% (23/31) vs 3.2% (1/31), P=0.001 |

**Even though this study was published only as abstract, it was included for the SU endpoint due to scarcity of data on SU (data reporting validated by checking data posted on Clinical trials.gov)*

*Note: 1 egg white contains about 4 g of protein; 1 peanut contains about 250 mg of protein*

**Case series and prospective cohort studies (single arm)**

- Wasserman, 2019^8^ (case series, N=270) – peanut: Patients were invited to test for SU if they had ≥ 36 months of maintenance dosing (a total of 105 patients), no ETRs within 2 years, ≥ 90% fall in peanut-specific IgE level or a prechallenge peanut-specific IgE < 1.0 kU/mL. After an avoidance period of 1 month, 19 patients were challenged (including 8 who did not meet these criteria) with 6 g peanut protein (24 peanuts); 74% (14/19) passed – among all patients who had ≥ 36 months of maintenance dosing: **13% (14/105)**
- Vickery, 2014^64^ (prospective cohort studies, age 1-16 years) – peanut: Of the 39 subjects originally enrolled, 12 (31%, 12/39) successfully passed a challenge with 5000 mg of peanut protein one month after stopping OIT.

**ADDITIONAL EVIDENCE**

**Smaller RCTs, CCTs or retrospective case-control studies (ITT analyses)**

| **Study** | **Design** | **Food allergen** | **Intervening period of allergen avoidance** | **SU – ITT analysis** | |
| --- | --- | --- | --- | --- | --- |
|  |  |  |  | **OIT** | **Comparator** |
| **Nagakura, 2018^65^, low-dose OIT** | Prospective cohort study with historical control | Peanut | 2 weeks | 33% (8/24) | Historical controls: 0 (0/23) |
| **Narisety 2015^66^** | RCT | Peanut | 4 weeks | 27% (3/11) | SLIT 10% (1/10) |
| **Takahashi, 2016^67^** | CCT | **Milk** | 2 weeks | 21% (7/31) – 1 year  45% (14/31) -2 years | Untreated: 0/17 (1 year) |
| **Vickery, 2017,^68^ USA age 9-36 months** | RCT comparing different maintenance doses with a substudy that retrospectively identified and matched standard of care controls | Peanut | 4 weeks | 85% (17/20) low dose maintenance  71% (12/17) high dose maintenance, Overall: 78% (29/37) | Historical matched controls: 3.9% (6/154), P<0.001 |
| **Yanagida 2016^69^, low-dose OIT** | RCT | Egg | 2 weeks | 71% (15/21) – 1/32 of one whole egg  33% (7/21) - 1/2 of one whole egg | Control: 0 (0/12) for both outcomes |

#### Allergens other than milk, egg and peanut

|  | **Nowak-Wegrzyn, 2019^24^ (RCT, N=46) -wheat** | **Kulmala, 2018^25^ (prospective, single-arm study, N=100) -wheat** | **Elizur, 2019^26^ (CCT, N=73)-walnut** | **Barni, 2019^27^ (case series, N=43) - hazelnut** | **Nachshon, 2019^28^ (CCT) N=75 - sesame** |
| --- | --- | --- | --- | --- | --- |
| **Age** | Median, 8.7 years; range, 4.2-22.3 | Mean 11.6 years, range, 6.1-18.6 | Mean 7.9 years OIT, 6.8 years control; range 4-20 | Mean 10 years, range: 5-16 | Mean 7.5 years, ≥4, (IQR: 5.8-11.6) |
| **Diagnosis** | DBPCFC | Open OFC (not performed in 15 patients with an immediate reaction within the previous 3 months) | OFC, unless an immediate, recent (past year) reaction was documented | OFC | OFC or reaction within past year together with positive skin prick test (SPT) result and/or specific serum IgE (>0.35 kUA/L) |
| **Sustained unresponsiveness** | At year 2, 7/23 (30%) low-dose VWG OIT-treated subjects were desensitized to 7443 mg of WP  **At year 2, 3/23 (13%) low-dose VWG OIT-treated subjects achieved SU 8 to 10 weeks off therapy to (SCD, 7443 mg of WP in DBPCFC)** | NA | **No data on** SU - (45 of 56 patients who had been desensitized to walnut had follow-up data (≥ 6 months after end of OIT) all of them (100%) maintained walnut desensitization for ≥6 months after reaching maintenance (passed OFC with single dose of 4000 mg walnut protein) – 70% (45/64) of patients who had initiated OIT) | NA | NA |

DBPCFC: double-blind, placebo-controlled food challenge; SCD: successfully consumed dose; VWG: vital wheat gluten

*Note: 7 walnuts correspond to approx. 28.3 g (1 ounce serving); 12 hazelnuts correspond to approx. 28.3 g (1 ounce serving), with 1 hazelnut weighting about 2.4 g*

#### Assessment of the quality of evidence

|  |  | **Risk of bias** (Cochrane tool^57^ for interventional comparative studies; IHE tool^58^ for case series) | **Level of evidence (Oxford Centre for Evidence-based medicine) -** adapted using the approach of the European Academy of Allergy and Clinical Immunology^59^ |
| --- | --- | --- | --- |
| **Peanut** | **Interventional comparative studies (RCTs, CCTs)** - *Tang 2015^4^* | **Low**- 1 RCT considered at low risk of bias | Level I (Systematic reviews, meta-analysis, randomized controlled trials) |
|  | **Case series –** Wasserman, 2019^8^ | **High –** 1 case series; main limits: single-center, retrospective data collection, only a small proportion of patients was assessed for SU |  |
| **Egg** | **Interventional comparative studies (RCTs, CCTs) – meta-analysis (88% /12% milk):** *including Burks 2012,^32^ Caminiti, 2015,^34^ Escudero, 2015, Staden, 2007^41^) ** | **Moderate** – 4 RCTs, of which at least 2 are considered being at high risk of bias | Level I (Systematic reviews, meta-analysis, randomized controlled trials) |
|  | **Case series** | **--** |  |
| **Milk** | **Interventional comparative studies (RCTs, CCTs):** Takahashi, 2016^67^ | **High** – 1 small open-label CCT** | Level II two groups, non-randomized studies |
|  | **Case series**: **-** NA | **--** |  |
| **Wheat** | **Interventional comparative studies (RCTs, CCTs):** Nowak-Wegrzyn, 2019^24^ | **Medium** – 1 small RCT with no control data | Level III: one group non-randomized |
|  | **Case series**: NA | **--** |  |
| **Walnut** | **Interventional comparative studies (RCTs, CCTs)** | **--** |  |
|  | **Case series**: NA | **--** |  |
| **Hazelnut** | **Interventional comparative studies (RCTs, CCTs)**:NA | **--** |  |
|  | **Case series:** NA | **--** |  |
| **Sesame** | **Interventional comparative studies (RCTs, CCTs)**: NA | **--** |  |
|  | **Case series**: NA | **--** |  |

*The quality of the study published in abstract form only (Sampson, 2019^63^ ) was not assessed

** The control group were patients who did not want to undergo OIT

### Impact of patient characteristics on efficacy outcomes

- Most OIT studies enrolled children and adolescents starting from age 4 to 7 years, with median/mean ages in the range of 6 to 12 years (Anagnostou, 2014;^1^ Bird, 2018;^5^ Burks, 2012;^32^ Elizur, 2016;^16^ Escudero,2015;^18^ Fuentes-Aparicio, 2013;^19^ Kauppila, 2019;^15^ Levy, 2014;^14^ Longo, 2008;^10^ Martin-Munoz, 2019;^20^ Nachshon, 2018;^7^ Reier-Nilsen, 2019;^3^ Vickery, 2018^6^ and Wasserman, 2019^8^). Three studies enrolled peanut-allergic children starting from a younger age, three years (Blumchen, 2019;^2^ Wasserman, 2014^56^) or one year (Tang, 2015^4^). One study enrolled children for milk or egg OIT starting from the age of one year (Morisset, 2007^12^).
- **Older children, adolescents and adults**
  - Association between baseline age and likelihood of achieving efficacy outcomes in large case series of children and adolescents (N: 145 to 295):
    1. Wasserman (2019^8^) reported (based on multivariate analysis) that among a cohort of 270 **4- to 18**-year old children and adolescents with **peanut** allergy (mean age 8.1 years), older age at the initiation of therapy significantly reduced the likelihood of reaching the target maintenance dose by 17% for each year after the age 5 years (odds ratio 0.83 [95% CI 0.75-0.93], P< .001) (see figure below).

- - 1. Other case series did not find such an association:
       1. Kauppila (2019^15^) observed (based on logistic regression analysis) that baseline age (<7 versus ≥7 years) was **not** a statistically significant predictor of treatment failure among 296 patients **5 to 17** years of age (median **7.5**) undergoing **milk** OIT.
       2. Levy (2014^14^) reported that among 280 **4- to 27**-year old patients (median **7.5** years) undergoing **milk** OIT there was no difference in median age between those who did or did not achieve the full dose (P=0.69).
       3. Nachshon (2018^7^) following 145 patients undergoing **peanut** OIT (age ≥ 4 years, median 5.8, interquartile range: 4.5-7, **88% were between 4 and 10** years old, 4.1% were >18 years old) observed that the 12 patients who failed desensitization were significantly younger than the 133 patients who were partially or fully desensitized (median 4.2 vs 6 yrs).
  - A double-blind, placebo-controlled RCT included 169 **adolescents** (age 12 to 17) and 55 **adults** (age 18 to 55) with peanut allergy (Burks, 2018;^70^ Vickery 2018^6^), with the percentage of adults who could tolerate ≥ 600 mg as a single dose being pre-specified as a secondary endpoint. There was no statistically significant difference between the OIT and the placebo groups in reaching this end-point.

| **Age range (years)** | **Proportion of patients who tolerated ≥ 600 mg peanut protein as a single dose: OIT versus placebo (n/N) – ITT analysis** | **Difference between OIT and placebo groups (95% CI)** |
| --- | --- | --- |
| **4 to 11** | 70.6% (168/238) versus 4.5% (4/89) | 66.1% (53.9 to 78.3), P<0.0001 |
| **12 to 17** | **61.2% (82/134) versus 2.9% (1/35)** | **58.3% (39.7 to 76.9), P<0.0001** |
| **18 to 55** | **41.5% (17/41) versus 14.3% (2/14)** | **27.2% (-1.7 to 56.0), P=0.07** |

- - Double-blind, placebo-controlled RCT of **adolescents** with peanut allergy (mean age 15 years, range 12-18; age at diagnosis: 3 years) (Fauquert, 2018^55^): Peanut (or placebo) capsules were ingested daily over 24 weeks with increments every 2 weeks from 2 to 400 mg of peanut protein. ITT analysis: at DBPCFC, unresponsiveness to 400 mg of peanut protein was achieved in 81% (17/21) of OIT and 11% (1/9) of placebo patients (P<0.001, absolute difference = 0.7, 95%IC 0.43 0.96).
  - Case series of 23 **adults** with OFC-confirmed IgE-mediated allergies who were treated with OIT (10 milk, 9 peanut, 4 egg) (Mantyla, 2018^71^): The median period of OIT was 515 days. The median dose of protein that could be tolerated increased from baseline 60-fold, 8-fold, and 35-fold in peanut, milk and egg-allergic subjects, respectively.
- **Toddlers and pre-school children:**
  - Vickery et al (2017^68^) randomized 37 children aged 9 to 36 months with OFC-confirmed peanut allergy to OIT with a maintenance dose of 300 or 3000 mg peanut protein per day. In the ITT analysis, 81% were desensitized to 20 peanuts (5000 mg peanut protein, OFC). Over a median of 29 months, an overall of 78% (29/37) children achieved a 4-week SU with 5000 mg of peanut protein (20 peanuts) (300 mg arm: 17/20 [85%]; 3000 mg, 12/17 [71%], P=0.43. Among 154 retrospectively identified matched control children who received standard care (avoidance), 6 demonstrated SU (3.9%), which was significantly lower than for the OIT group (P<0.001).
  - Martorell et al (2011^11^) randomized 60 children aged 24 to 36 months with OFC-confirmed milk allergy to OIT or avoidance. After one year, 90% (27/30) of OIT patients tolerated 200 mL milk compared to 23% (7/30) of control patients.
  - Soller et al 2019 (prospective multi-center study) reported that of 270 pre-school children (age 0.75-5.9 years, median 1.9 years) initiating peanut OIT, 243 (90%) reached the maintenance dose of 300-320 mg peanut protein (1.2-1.3 peanuts). ^9^ Of these, 87 patients had had a baseline reaction and an OFC after ~1 year of ingesting ~300mg peanut protein daily and 68 of them passed the OFC (78% = 68/87). For the 19 (21.9%) who reacted at the 1-year OFC, median cumulative dose increased from 12 mg (IQR: 10, 75) at baseline to 4000 mg (IQR: 4000 to 4000) at the 1-year OFC (change from baseline: 3988 mg [IQR: 3920, 3995]). (unpublished data on file from Canadian clinical practice)

### Correlations between baseline parameters and OIT efficacy outcomes

Outcomes of RCTs with N≥ 50 that included children with a history of severe reactions:

- **Anagnostou, 2014^1^ (peanut** RCT, N=99, baseline worst clinical reaction WAO score grade 3 or 4: control 22.5% vs active 8.1%): **62%** (24/39; 95% CI 45–78) of patients were desensitized in the active group and none of the control group (0/46; 95% CI 0–9; P<0.001). 84% (95% CI 70–93) of the active group tolerated daily ingestion of 800 mg protein (5 peanuts). Median increase in peanut threshold after OIT was 1345 mg (range 45–1400; P<0.001) or 25.5 times (range 1.82–280; P<0.001).
- **Blumchen, 2019^2^** (**peanut**, N2=62: 31 OIT, 31 control): At baseline, 53% of children in the OIT group and 58% in the control group had a history of severe allergic reactions to peanut (grade IV or V^72^). The median sIgE was 81.5 kU/L (range 0.57-624 kU/L) and the median maximum tolerated single dose at the initial OFC was 30 mg peanut protein (range 1-3,000 mg). At the completion of the maintenance phase, 23/31 patients (**74%)** in the OIT group tolerated ≥ 300 mg peanut protein compared to 5/31 patients (**16%**) in the placebo group (P<.001); 13 (42%) patients in the OIT group tolerated 4.5 g peanut protein vs 1 patient (3.2%) in the placebo group (P<.001).
- **Longo, 2008^10^** (**milk** RCT, N=30 OIT, N=30 control): Included children with a baseline sIgE level > 85 kUA/L who had a positive history of at least one severe allergic reaction (defined as class 4 and 5 by Clark’s classification) after accidental exposure to milk or dairy products requiring emergency treatment. After 1 year, 11/30 **(36%)** children receiving OIT achieved a daily intake of cow’s milk of at least 150 mL, **54%** (16/30) were able to consume 5 to 150 mL of milk daily, and 10% (3/30) discontinued due to adverse reactions (respiratory or abdominal). No desensitization was observed in the control group.
- **Martin-Munoz, 2019a^20^ (egg, RCT N=101 Age: 6-9 years):**  Passing DBPCFC at 12 months with 3.3 g pasteurized raw egg white protein (cumulative) or reaching target dose of 3.3 g pasteurized raw egg white egg white protein (equivalent to 1 egg): OIT: **84%** (64/76) Control: **16%** (4/25)
- **Reier-Nilsen, 2019a^3^** (**peanut**, N=77: 57 OIT, 20 control): At baseline, 79% of children had a history of anaphylaxis to peanut and all children reacted with anaphylaxis during the baseline DBPCFC. At the end of the study, **21%** (12/57) of the OIT children reached the maintenance dose of 5000 mg peanut protein**, 54%** (31/57) reached a lower maintenance dose (250-4000 mg) and 25% (14/57) had discontinued. The only significant predictor of reaching a maintenance dose was the baseline peanut sIgG4/sIgE ratio (P=0.02).

**Large case series**

| **Baseline parameter** | **Outcome and correlation** | **Reference** |
| --- | --- | --- |
| **History of anaphylactic reaction** | Proportion (n/N) of patients with history of anaphylaxis (n) among all patients achieving the following outcomes (N):   - Achieving the full target dose: 64% (102 [with history of anaphylaxis]/160 [all patients achieving this outcome]) - Achieving a partial dose: 91% (60/66) - Did not achieve any cow milk protein consumption: 82% (30/39) (P<0.001) | Levy, 2014^14^ (milk, N=280) |
|  | In multivariate analysis, history of anaphylaxis was **not** associated with reaching the maintenance dose. | Wasserman, 2019^8^ (peanut, N=270) |
| **Specific IgE serum levels** | In logistic regression analysis, **treatment failure** (< 200 mL milk daily in long-term follow-up) was related to milk sIgE before OIT (P=0.000) | Kauppila, 2019^15^ (milk, N=296) |
|  | An increase of 1 KU/L in the pre-treatment peanut-specific IgE level led to a 2% decrease (P<.001) in the **likelihood of reaching the target dose** | Wasserman, 2019^8^ (peanut, N=270) |
| **Maximum tolerated dose of food allergen** | Starting dose: median 120 mg cow milk protein; range (10-7200 mg). In multivariate logistic regression analysis, the most **important independent predictors for achieving the full dose included a maximal tolerated starting dose ≥ 30 mg of cow milk protein** (OR: 4.6, P<.001). | Levy, 2014^14^ (milk, N=280) |

**Kauppila, 2019^15^ Wasserman, 2019^94^**

**ADDITIONAL EVIDENCE - RCTs, CCTs and smaller case series**

| **Baseline parameter** | **Outcome and correlation** | **Reference** |
| --- | --- | --- |
| **History of anaphylactic reaction** | History of anaphylaxis was **not** a potential predictor of food challenge results at 4 months. | Escudero, 2015^32^ (RCT **egg**, N=30 OIT, N=31 control) |
| **Specific IgE blood levels** | In Tobit regression analysis, the baseline total IgE and the baseline sIgE were associated with the amount of peanut protein tolerated after OIT (P<0.001 for all). | Anagnostou, 2014^2^ (RCT **peanut**, N=49 OIT, N=50 control) |
|  | Desensitization was slower in patients with higher baseline sIgE levels: 9/10 (90%) patients in with sIgE <3.5 kU/L, 7/15 (50%) of patients with sIgE=3.5-17 kU/L, and 3/8 (30%) of patients with sIgE=17-50 kU/L achieved desensitization in < 3 months (P = 0.04). | Garcia-Ara, 2013^43^ (CCT, **milk**, N=36 OIT; N=19 control) |
| **Maximum tolerated dose of food allergen** | In Tobit regression analysis, the amount of peanut protein tolerated at baseline was associated with the amount of peanut protein tolerated after OIT (P<0.001 for all). | Anagnostou, 2014^2^ (RCT **peanut**, N=49 OIT, N=50 control) |
|  | The dose that triggered symptoms at baseline was not a potential predictor of food challenge results at 4 months. | Escudero, 2015^32^ (RCT **egg**, N=30 OIT, N=31 control) |
|  | Baseline maximum dose allergen dose tolerated on the first day was not predictive of sustained unresponsiveness. | Burks, 2012^32^ (RCT **egg**, N=40 OIT, N=15 control) |

### Correlations between baseline asthma status and OIT outcomes

**Large case series**

| **Outcome and correlation** | **Proportion of patients with asthma** | **Reference** |
| --- | --- | --- |
| Compared to patients without asthma (N=93), patients with asthma (N= 101) were **less likely to reach full desensitization** (7200 mg CMP) (52% vs 69%, P =.019). Among patients with asthma, need for controller therapy (initiated before or during OIT) was associated with lower likelihood of reaching full desensitization (P=0.001). Overall, 86% of all patients with asthma (87/101) and ≥ 82% of the sub-group requiring asthma controller therapy reached a dose likely protective against accidental exposure (>180 mg CMP). | 101 patients with asthma, 93 no asthma | Elizur, 2015^73^ (**milk**, N=194) |
| Baseline asthma was **not a statistically** significant predictor of treatment failure. | asthma: 73% | Kauppila, 2019^15^ (**milk,** N=244) |
| History of asthma (intermittent, P=.291, or persistent, P = .170) was **not** a statistically significant predictor of reaching the target dose. | asthma: persistent: 43%, intermittent: 21% | Wasserman, 2018^8^ (**peanut**, N=270) |
| Although more children with asthma (23%) vs without asthma (5.6%) had a single highest tolerated dose <5 mg peanut protein (P=.003), the two groups were **comparable in pre-OIT reaction severity and in the course and outcome of OIT**. | asthma: 56/145 [39%], controller therapy before OIT or initiated at the start of OIT: 34/145 [23%], uncontrolled asthma excluded | Nachshon, 2018,^7^ (**peanut** , N=145) |

### Correlations between baseline multiple food allergies single-food OIT outcomes in large case series

| **Outcome and correlation** | **Reference** |
| --- | --- |
| Percent of patients with other food allergies within the following outcome groups:   - Achieved the full target dose: 18% - Achieved partial dose: 17% - Did not achieve any cow milk protein consumption: 13%, P=0.42 | Levy, 2014^14^ (**milk**, N=280) |
| Other food allergies:   - Continued treatment: 49% (47/97) - Stopped treatment: 21% (3/14), P=0.08 | Nachshon, 2018^7^ (**peanut**, N=111) |

## CLINICAL SAFETY

### Severe reactions /Systemic reactions/ anaphylaxis / serious adverse events / epinephrine use

#### Data for peanut, milk, egg allergies

| **Meta-analyses** | | |
| --- | --- | --- |
| **Nurmatov, 2017^29^ – chicken’s egg, cow’s milk, peanut** | **Romantsik, 2018^30^ – chicken’s egg** | **Chu, 2019^31^ – peanut** |
| Absence of **systemic reactions** as assessed in 4 RCTs^34,36,38,42^ and 1 CCT:^46^  **-** milk/egg/peanut: 61%/21%/19%  **OIT: 84%% (73/87)**  **Control: 98% (62/63)**  RR (C/OIT) = 1.16 (95% CI 1.03, 1.30)  *Proportion of patients with systemic reactions:*  *OIT: 16% (14/87)*  *Control: 1.6% (1/63), P: significant* | Proportion of participants with **serious AEs (defined as ETR**) as assessed in 9 RCTs^18,19,32,34,35,37,51-53^ and 1 CCT:^54^  **OIT: 8.4% (21/249)**  **Control: 0 (0/190)**  RR= NA  *GRADE assessment: low quality of evidence* | Proportion of participants with **ETRs** as assessed in 9 RCTs^1-6,42,55,66^  **OIT: 12% (78/660)**  **Control: 3.7% (12/324)**  RR= 2.21 (1.27 to 3.83)  *GRADE assessment (overall certainty of evidence): High*  Proportion of participants with **anaphylaxis** as assessed in 9 RCTs^1,3-6,42,55,66^ (1 unpublished):  **OIT: 17% (108/653)**  **Control: 2.6% (8/297)**  RR=3.12 (95% CI 1.76, 5.55)  *GRADE assessment (overall certainty of evidence): High*  Proportion of participants with **serious AEs*** as assessed in 9 RCTs^1-6,42,55,66^ (3 unpublished)  **OIT: 6.2% (43/699)**  **Control: 3.0% (10/338)**  RR=1.92 (95% CI 1.00, 3.66)  *GRADE assessment (overall certainty of evidence): Moderate*  *Re-analysis to correct misclassification:* see section **8.2**  **OIT: 21/699 (3.0%)**  **Control: 11/338 (3.3%)** |

ELORS: eosinophilic esophagitis-like oral immunotherapy- related syndrome; ETR: epinephrine-treated reaction; DBPCFC: double-blind placebo-controlled food challenge; RR: risk ratio (random effects model)

*Serious adverse events defined by US FDA as: causing death, a life-threatening state, hospitalization, disability, congenital abnormality, or an important medical event such as an urgent intervention to prevent the other outcomes) and if they caused treatment discontinuation

| **Case series with N> 150** | | | | |
| --- | --- | --- | --- | --- |
| **Levy, 2014^14^ (Israel)– cow’s milk**  **N=265**  **Age: 4-27 (7.5 median) years**  **Diagnosis confirmed with OFC: not for all patients** | **Kauppila, 2019^15^ (Finland) – cow’s milk**  **N=295**  **Age: 5-17 (median 7.5) years**  **Diagnosis confirmed with OFC: yes** | **Soller, 2019^9^ (Canada) – peanut**  **N=270**  **Age: 0.75-5.9 (median 1.9, IQR : 1.25-2.75) years**  **Diagnosis confirmed with OFC: for 31% of patients** | **Wasserman, 2019^8^ (USA) – peanut**  **N=270**  **Age: 4-18 (mean: 8.1) years**  **Diagnosis confirmed with OFC: no** | **Wasserman, 2014^56^ (USA and Israel)- peanut**  **N=352**  **Age: 3-24 years**  **Diagnosis confirmed with OFC: 97% of patients** |
| **Follow-up:** ≥ 10 months after start of OIT  **ETRs** during induction cycles (in clinic): **48%** (128/265) of patients  **ETRs** during home-dosing phase: **17% (44/265**) of patients (58 ETRs per 77,098 doses= **0.08%**)  % of ETRs at home= 58/186=**31%**  **SEAs**: not reported | **Follow-up:** median 6.5 (range 1-11) years after start of OIT  **Anaphylaxis** at least once after build-up phase: **14%** (34/237)- data missing from 59/295 patients  **Possible SEAs**: One extremely severe anaphylaxis occurred in a patient after consumption of milk yogurt with a high concentration of cow's milk protein (8 g/100 mL), while he was on the aimed maintenance dose | **Follow-up:** median 20.0 weeks (build-up only)  **Possible SAE:**  **Grade 4 (severe symptoms)** (respiratory failure or hypotension or profound lethargy): **0.4%** (1/270) (1 per 2,321 doses administered in the office=0.04%)  **ETR** during build-up phase: **4.1%** (11/270) of patients (12 ETRs per 41,020 doses = **0.029%** of doses with ETR)  ETRs during in-office build-up: 6 per 2,321 doses = 0.26%  ETRs during home dosing build-up: 6 per 38,699 doses = **0.016%**  50% of ETRs occurred at home  1.1% (3/270) of patients visited emergency departments for AEs, all of which were Grade 2.  Grading according to Cox 2010^74^ (modified) | **Follow-up**: ≥ 1 month after reaching maintenance and up to 8 years  **ETRs** during build-up phase: **23%** (63/270) of patients (100 ETRs in total [37%] occurred in clinic)  **SAEs**: 5 of 100 ETRs required 2 epinephrine doses and 2 required 3 doses; no ETR required > 3 epinephrine doses or intravenous fluids  **ETRs** during maintenance phase: **13%** (28/214) of patients (63 ETRs in total, 40% occurred during the first 6 months) - sub-group: **patients treated for ≥ 3 years of maintenance**: **17%** (18/105) (43 ETRs)  Incidence of ETRs during maintenance phase: 9.9/100 patient-years | **Follow-up**: from a few weeks to >7 after reaching maintenance  **ETRs** during build-up phase: **10%** (36/352) of patients (57 ETRs per 79,726 doses = **0.07%** of doses with ETR - *corresponds to 26.1 ETRs per 100 patient-years, assuming 1 dose per day*)  **ETRs** during maintenance phase: **6.4%** (19/298) of patients (38 ETRs per 160,265 doses, **0.02%** - *corresponds to 8.7 ETRs per 100 patient-years, assuming 1 dose per day*)  Proportion of all ETRs that occurred in clinic: 29% (28/95)  **Possible SAEs**: 3 patients received 2 doses of epinephrine for a single ETR; no patient required intravenous fluids for hypotension or other manifestations of shock. |

**RCTs with N>50 not included in meta-analyses**

| **Martin-Munoz, 2019a^20^ (Spain) – chicken’s egg**  **N (OIT)=76 (88 including cross-over)**  **Age: 6-9 years**  **Diagnosis confirmed with OFC: yes** | **De Schryver, 2019^13^ (Canada) – cow’s milk**  **N (OIT) = 26 (41 including cross-over), 26 control (4 of them withdrew)**  **Age: 6-18, mean 12.1 years**  **Diagnosis confirmed with OFC: yes (single-blind)** |
| --- | --- |
| *Data from non-comparative phase:*  **ETRs during build-up phase:**  **OIT: 8.0%** (7/88) of patients  Control: NR  **Grade 4 dosing adverse reactions** during build-up phase:  **OIT: 9.2%** (7/76) of patients (15 DARs per 8448 doses, 0.2%)  Control: NR  **Grade 4 dosing adverse reactions** during maintenance phase:  **OIT: 1.3%** (1/76) of patients (1 reaction)  Control: NR  No patient had moderate or severe adverse reactions because of egg consumption once they had finished the OIT.  **Possible SAEs**:  Build-up phase: No patient developed dysrhythmia and/or severe hypotension, hypovolemic shock, laryngeal edema, or respiratory or cardiac arrest  Maintenance phase: One patient developed a grade 4 reaction after 2 months on maintenance after stopping OIT for 4 days due to intercurrent illness.  Grading according to Sampson 2003^75^ | *Data from comparative phase (first year of study):*  **Anaphylactic** allergic reactions (multi-organ reaction or hypotension):  **OIT (N=26)**: mean 5.5 reactions per patient  **Control (N=22)**: 2 moderate reactions per 22 patients (mean 0.1 per patient)  **ETRs:**  **OIT (N=26)**: mean 0.5 per patient (SD 0.9)  **Control (N=22)**: 2/22 patients (mean 0.1 per patient)  *Data from non-comparative phase:*  **Anaphylaxis**:  OIT (N=41): Number of anaphylactic reactions per patient: mean 6.0 (245 reactions per 41 patients), median 4 (range 0-21, IQR 1-10, SD 3.5)  **Severe anaphylactic reactions:**  OIT (N=41): 2 severe anaphylactic reactions in 2 patients: 4.9% (2/41) of patients  **ETRs:**  OIT (N=41): mean 0.6 per patient (SD 1.2)  Grading according to Muraro 2007^76^ |

#### Allergens other than milk, egg and peanut

|  | **Nowak-Wegrzyn, 2019^24^ (RCT, N=46) -wheat** | **Kulmala, 2018^25^ (case series, N=100) -wheat** | **Elizur, 2019^26^ (CCT, N=73)-walnut** | **Barni, 2019 (case series, N=43) - hazelnut** | **Nachshon, 2019^28^ (CCT) N=75 - sesame** |
| --- | --- | --- | --- | --- | --- |
| **Age** | Median, 8.7 years; range, 4.2-22.3 | Mean 11.6 years, range, 6.1-18.6 | Mean 7.9 years OIT, 6.8 years control; range 4-20 | Mean 10 years, range: 5-16 | Mean 7.5 years, ≥4, (IQR: 5.8-11.6) |
| **Diagnosis** | DBPCFC | Open OFC (not performed in 15 patients with an immediate reaction within the previous 3 months) | OFC, unless an immediate, recent (past year) reaction was documented | OFC | OFC or reaction within past year together with positive skin prick test (SPT) result and/or specific serum IgE (>0.35 kUA/L) |
| **Adverse reactions** | Among 7822 low-dose OIT doses in year 1, 15% were associated with adverse reactions: 0.04% were severe, and 0.08% (were ETRs.  Among 7921 placebo doses, 5.8% were associated with adverse reactions; none were severe.  SAEs:  Low-dose OIT: 2/23 (including 1 patient who was not randomized)  Placebo: 5/23 | Allergic symptoms occurred in 94/100 children: mild in 34, moderate in 36 and severe in 24 (24%). 12 patients (12%) had 13 ETRs  % of patients with severe reactions by phase:  Build-up (17 weeks – 3.3 months): 14% (14/100)  Maintenance 1 (3 months): 7.8% (6/77)  Maintenance 2 (9 months): 8.3% (6/72)  Severe reactions were defined as objective respiratory symptoms, such as extensive coughing, inspiratory stridor or expiratory wheezing, and cardiovascular symptoms such as unconsciousness, lethargy, collapse, drop in blood pressure or tachycardia, alone or in combination with other symptoms. During the study, only bronchial wheezing, coughing, and laryngeal stridor occurred; no patient experienced a drop in blood pressure or cardiovascular collapse. | 47/55 (85%) patients had an adverse reaction (mostly Grade 1 or 2) during up-dosing in the clinic and 40 (73%) during the home-dosing phase  The reactions were mostly mild (Grade 1 or 2) and occurred in response to 109 (4%) of the doses in clinic and 244 (2%) of home doses  ETRs: 11 (20%) during the inhospital study phase and 8 patients had an ETR in response to a dose at home.  During the maintenance phase, 1/45 patients had 1 ETR  No severe reactions (≥ grade 3) were recorded during the study. | 20/43 patients (46.5%) had no reactions and 23/43 patients had a total of 55 reactions: 34 (61.8%) oral allergy syndrome, 8 (14.5%) rash, 6 (10.9%) abdominal pain, 2 (3.6%) urticaria, 2 (3.6%) angioedema, and 3 (5.4%) dyspnea | Build-up phase:  Adverse reactions occurred in 127 of 2,720 (4.7%) induction doses and 253 of 13,170 (2%) home doses  ETR during the hospital build-up phase: 10 patients (16.7%) for 13/127 (9.4%) reactions  ETR during home treatment: 5 patients (8.3%) for 7/ 253 (2.8%) reactions.  No reactions worse than Grade I or II (WAO) occurred  Maintenance phase:  No ETR  Grading according to Cox 2010^74^ (modified) |

ETR: epinephrine-treated reaction; DBPCFC: double-blind, placebo-controlled food challenge; SCD: successfully consumed dose; VWG: vital wheat gluten

#### Assessment of the quality of evidence

|  |  | **Risk of bias** (Cochrane tool^57^ for interventional comparative studies; IHE tool^58^ for case series) | **Level of evidence (Oxford Centre for Evidence-based medicine) -** adapted using the approach of the European Academy of Allergy and Clinical Immunology^59^ |
| --- | --- | --- | --- |
| **Peanut** | **Interventional comparative studies (RCTs, CCTs):** meta-analysis (8 RCTs^1,2,4-6,42,55^, 1 unpublished) | **Low**- 9 RCTs of which 8 rated as being at overall low risk of bias for ETRs and anaphylaxis | Level I (Systematic reviews, meta-analysis, randomized controlled trials) |
|  | **Case series:** Soller, 2019^9^, Wasserman, 2019,^8^ Wasserman, 2014^56^ | **Moderate** – 3 case series: 2 with retrospective study design |  |
| **Egg** | **Interventional comparative studies (RCTs, CCTs):** Romantsik, 2018^30^ (9 RCTs^18,19,32,34,35,37,51-53^ and 1 CCT^54^) | **High** – 10 RCTs of which all were rated as being at overall high risk of bias by ≥1 analysis | Level I (Systematic reviews, meta-analysis, randomized controlled trials) |
|  | **Case series** | **--** |  |
| **Milk/egg/ peanut** | **Interventional comparative studies (RCTs, CCTs):** meta-analysis (4 RCTs^34,36,38,42^ and 1 CCT) | **Moderate** – RCTs (3 low, 1 high risk of bias) and 1 CCT (moderate risk of bias) | Level I (Systematic reviews, meta-analysis, randomized controlled trials) |
| **Milk** | **Interventional comparative studies (RCTs, CCTs):** 1 RCT (De Schryver, 2019^13^) | **High** – unblinded, 4 control patients withdrew | Level I (Systematic reviews, meta-analysis, randomized controlled trials) |
|  | **Case series**: Levy, 2014^14^ and Kauppila, 2019^15^ (clinical practice) | **Moderate** – 2 case series, main limit: retrospective study design |  |
| **Wheat** | **Interventional comparative studies (RCTs, CCTs)**: Nowak-Wegrzyn, 2019^24^ | **Low** – 1 RCT | Level I (Systematic reviews, meta-analysis, randomized controlled trials) |
|  | **Case series**: 1 prospective case series in research context (Kulmala, 2018^25^) | **Low** – 1 prospective case series |  |
| **Walnut** | **Interventional comparative studies (RCTs, CCTs)**: Elizur, 2019^26^ (CCT, N=73) | **High** – non-randomized control group,** unblinded | Level II (two groups, non-randomized studies) |
|  | **Case series**: NA | **--** |  |
| **Hazelnut** | **Interventional comparative studies (RCTs, CCTs)**:NA | **--** | Level II (two groups, non-randomized studies) |
|  | **Case series:** Barni, 2019^27^ (clinical practice) | **High** - 1 case series from clinical practice, limits: retrospective study design, single center, unclear recruitment and eligibility criteria |  |
| **Sesame** | **Interventional comparative studies (RCTs, CCTs)**: Nachshon, 2019^28^ | **High** – non-randomized control group,*** unblinded | Level II (two groups, non-randomized studies) |
|  | **Case series**: NA | **--** |  |

### Any adverse reactions / any allergic reactions / local reactions

#### Data for peanut, milk and egg allergy

| **Meta-analyses** | | | **RCT with N>50** | | **Case series with N> 150** | | |
| --- | --- | --- | --- | --- | --- | --- | --- |
| **Nurmatov, 2017^29^ – chicken’s egg and cow’s milk** | **Romantsik, 2018^30^ – chicken’s egg** | **Chu, 2019^31^ – peanut** | **Martin-Munoz, 2019^20,21^ – chicken’s egg** | **De Schryver, 2019^13^ (Canada) – cow’s milk**  **N (OIT) = 26 (41 including cross-over), 26 control (4 of them withdrew)**  **Age: 6-18, mean 12.1 years**  **Diagnosis confirmed with OFC: yes (single-blind)** | **Levy, 2014^14^ (Israel)– cow’s milk**  **N=265**  **Age: 4-27 (7.5 median) years**  **Diagnosis confirmed with OFC: not for all patients** | **Wasserman, 2019^8^ (USA) – peanut**  **N=270**  **Age: 4-18 (mean: 8.1) years**  **Diagnosis confirmed with OFC: no** | **Soller, 2019^9^ (Canada) – peanut**  **N=270**  **Age: 0.75-5.9 (median 1.9, IQR : 1.25-2.75) years**  **Diagnosis confirmed with OFC: for 31% of patients** |
| Absence of **local reactions** as assessed in 6 RCTs^11,12,32,34,37,38^ and 1 CCT:^46^ (Fig S21) - egg/milk: 61%/39%  **OIT: 49% (89/183)**  **Control: 98% (133/136)**  RR (C/OIT) = 2.14 (95% CI 1.47, 3.12  *Proportion of patients with local reactions:*  ***OIT: 51% (94/183)***  ***Control: 2.2% (3/136)****,* ***P: significant*** | Number of participants with **mild to severe AEs** as assessed in 9 RCTs^18,19,32,34,35,37,51-53^ and 1 CCT:^54^  **OIT: 75% (187/249)**  **Control: 6.8% (13/190)**  RR (OIT/C) = 8.35 (95% CI 5.31, 13.12)  *GRADE assessment: low quality of evidence* | **Vomiting**^††^ as assessed in 6 RCTs^2,4-6^ (2 unpublished):  **OIT: 39% (201/519)**  **Control: 19% (44/236)**  RR=1.79 (95% CI 1.35, 2.38)  *GRADE assessment (overall certainty of evidence): High*  **Angioedema**^‡‡^ as assessed in 5 RCTs:^4-6^ (2 unpublished)  **OIT: 10% (51/489)**  **Control: 3.9% (8/205)**  RR=2.25 (95% CI 1.13, 4.47)  *GRADE assessment (overall certainty of evidence): High*  **Nasal congestion or blockage**,^§§^ as assessed in 6 RCTs:^4-6,55^ (2 unpublished)  **OIT: 29% (149/510)**  **Control: 18% (38/214)**  RR=1.36 (95% CI 1.02, 1.81)  *GRADE assessment (overall certainty of evidence): Moderate*  ***Any allergic/adverse reaction***: 86% OIT (N=639) vs 61% control (N=278), RR=1.34 (1.12 to 1.60) | **Dose-adverse reactions (DARs):**  **OIT: 87%** (66/76)  **Control: 32%** (8/25)  P<.001  DARs decreased in number and severity throughout the OIT and throughout the study (P < 0.05).  **DARs during build-up phase:**  **OIT: 91%** (67/76) of patients (420 DARs per 8448 doses, 4.9%)  Control: NR  **DARs during maintenance phase:**  **OIT: 71%** (54/76) of patients (87 DARs)  Control: NR | No comparative data for non-anaphylactic reactions | **Frequency of reactions during** home-dosing phase which were **not treated**:  **OIT: 0.6%** (476 reactions per 77,098 doses)  **Frequency of reactions during** home-dosing phase which were **treated with an antihistamine or a bronchodilator**:  **OIT: 1.2%** (960 reactions per 77,098 doses) | Proportion of patients who reported **reactions that did not require**  **epinephrine use**:  **OIT: 58%** (157/270) patients (330 reactions) | ≥ 1 allergic reaction during buildup phase: 68% (183/270)  Grade 1 (mild) symptoms: 36%  Grade 2 (moderate) symptoms: 31%  Number of allergic reactions per patient: 1.99 (=538/270) |

††Similar findings for abdominal pain, mouth itching, and any allergic or adverse reaction. ‡‡Similar findings for urticaria. §§ Similar findings for asthma attack or wheeze

#### Allergens other than milk, egg and peanut

|  | **Nowak-Wegrzyn, 2019^24^ (RCT, N=46) -wheat** | **Kulmala, 2018^25^ (case series, N=100) -wheat** | **Elizur, 2019^26^ (CCT, N=73)-walnut** | **Barni, 2019 (case series, N=43) - hazelnut** | **Nachshon, 2019^28^ (CCT) N=75 - sesame** |
| --- | --- | --- | --- | --- | --- |
| **Age** | Median, 8.7 years; range, 4.2-22.3 | Mean 11.6 years, range, 6.1-18.6 | Mean 7.9 years OIT, 6.8 years control; range 4-20 | Mean 10 years, range: 5-16 | Mean 7.5 years, ≥4, (IQR: 5.8-11.6) |
| **Diagnosis** | DBPCFC | Open OFC (not performed in 15 patients with an immediate reaction within the previous 3 months) | OFC, unless an immediate, recent (past year) reaction was documented | OFC | OFC or reaction within past year together with positive skin prick test (SPT) result and/or specific serum IgE (>0.35 kUA/L) |
| **Adverse reactions** | Among 7822 low-dose OIT doses in year 1, 15% were associated with adverse reactions: 0.04% were severe, and 0.08% (were ETRs.  Among 7921 placebo doses, 5.8% were associated with adverse reactions; none were severe.  SAEs:  Low-dose OIT: 2/23 (including 1 patient who was not randomized)  Placebo: 5/23 | Allergic symptoms occurred in 94/100 children: mild in 34, moderate in 36 and severe in 24 (24%). 12 patients (12%) had 13 ETRs  % of patients with severe reactions by phase:  Build-up: 14% (14/100)  Maintenance 1 (3 months): 7.8% (6/77)  Maintenance 2 (9 months): 8.3% (6/72) | 47/55 (85%) patients had an adverse reaction (mostly grade 1 or 2) during up-dosing in the clinic and 40 (73%) during the home-dosing phase  The reactions were mostly mild (grade 1 or 2) and occurred in response to 109 (4%) of the doses in clinic and 244 (2%) of home doses  ETRs: 11 (20%) during the inhospital study phaseand 8 patients had an ETR in response to a dose at home.  During the maintenance phase, 1/45 patients had 1 ETR  No severe reactions (≥ grade 3) were recorded during the study. | 20/43 patients (46.5%) had no reactions and 23/43 patients had a total of 55 reactions: 34 (61.8%) oral allergy syndrome, 8 (14.5%) rash, 6 (10.9%) abdominal pain, 2 (3.6%) urticaria, 2 (3.6%) angioedema, and 3 (5.4%) dyspnea | Build-up phase:  Adverse reactions occurred in 127 of 2,720 (4.7%) induction doses and 253 of 13,170 (2%) home doses  ETR during the hospital build-up phase: 10 patients (16.7%) for 13/127 (9.4%) reactions  ETR during home treatment: 5 patients (8.3%) for 7/ 253 (2.8%) reactions.  No reactions worse than grade I or II (WAO) occurred  Maintenance phase:  No ETR  Grading according to Cox 2010^74^ (modified) |

ETR: epinephrine-treated reaction; DBPCFC: double-blind, placebo-controlled food challenge; SCD: successfully consumed dose; VWG: vital wheat gluten

#### Assessment of the quality of evidence

|  |  | **Risk of bias** (Cochrane tool^57^ for interventional comparative studies; IHE tool^58^ for case series) | **Level of evidence (Oxford Centre for Evidence-based medicine) -** adapted using the approach of the European Academy of Allergy and Clinical Immunology^59^ |
| --- | --- | --- | --- |
| **Peanut** | **Interventional comparative studies (RCTs, CCTs):** meta-analysis (Chu, 2019^31^) | **Low** | Level I (Systematic reviews, meta-analysis, randomized controlled trials) |
|  | **Case series:** Soller, 2019^9^, Wasserman, 2019^8^ | **Low** – 2 case series, of which 1 had a low and the other a moderate risk of bias |  |
| **Egg** | **Interventional comparative studies (RCTs, CCTs):** Romantsik, 2018^30^ (9 RCTs^18,19,32,34,35,37,51-53^ and 1 CCT^54^) plus 1 RCT (Martin-Munoz, 2019^20,21^) | **High** ––RCTs overall rated as being at high risk of bias | Level I (Systematic reviews, meta-analysis, randomized controlled trials) |
|  | **Case series** | **--** |  |
| **Egg** / **milk**: 61%/39% | **Interventional comparative studies (RCTs, CCTs):** meta-analysis: 6 RCTs^11,12,32,34,37,38^ and 1 CCT^46^ | **Moderate** – of the 6 RCTs in the meta-analysis, 3 low, 2 high, and 1 unclear risk of bias) plus 1 CCT | Level I (Systematic reviews, meta-analysis, randomized controlled trials) |
| **Milk** | **Interventional comparative studies (RCTs, CCTs):** NA | **--** | Level III: one group non-randomized |
|  | **Case series**: Levy, 2014^14^ (clinical practice) | **Moderate** – 1 case series, main limit: retrospective study design |  |
| **Wheat** | **Interventional comparative studies (RCTs, CCTs)**: Nowak-Wegrzyn, 2019^24^ | **Low** – 1 RCT | Level I (Systematic reviews, meta-analysis, randomized controlled trials) |
|  | **Case series**: 1 prospective case series in research context (Kulmala, 2018^25^) | **Low** – 1 prospective case series |  |
| **Walnut** | **Interventional comparative studies (RCTs, CCTs)**: Elizur, 2019^26^ (CCT, N=73) | **High** – non-randomized control group,** unblinded | Level II (two groups, non-randomized studies) |
|  | **Case series**: NA | **--** |  |
| **Hazelnut** | **Interventional comparative studies (RCTs, CCTs)**:NA | **--** | Level II (two groups, non-randomized studies) |
|  | **Case series:** Barni, 2019^27^ (clinical practice) | **High** - 1 case series from clinical practice, limits: retrospective study design, single center, unclear recrutement and eligibility criteria |  |
| **Sesame** | **Interventional comparative studies (RCTs, CCTs)**: Nachshon, 2019^28^ | **High** – non-randomized control group,*** unblinded | Level II (two groups, non-randomized studies) |
|  | **Case series**: NA | **--** |  |

### Discontinuations due to adverse events

#### Data for peanut, milk and egg allergy

| **Meta-analyses : Chu, 2019^31^ – peanut** | **RCT with N> 50 not included in meta-analysis** | **Case series with N> 150** | | |
| --- | --- | --- | --- | --- |
|  |  | **Levy, 2014^14^ (Israel)– cow’s milk**  **N=280***  **Age: 4-27 (7.5 median) years**  **Diagnosis confirmed with OFC: not for all patients** | **Kauppila, 2019^15^ (Finland) – cow’s milk**  **N=295**  **Age: 5-17 (median 7.5) years**  **Diagnosis confirmed with OFC: yes** | **Wasserman, 2019^8^ (USA) – peanut**  **N=270**  **Age: 4-18 (mean: 8.1) years**  **Diagnosis confirmed with OFC: no** |
| **Discontinuations due to AEs** as assessed in 9 RCTs^1-6,42,55,66^ (3 unpublished):  **OIT: 12% (87/699)**  **Control: 2.4% (8/338)**  RR=2.55 (95% CI 1.20, 5.42)  *GRADE assessment (overall certainty of evidence): high* | Martin-Munoz, 2019^20,21^ - **egg**  Discontinuations due to AEs during build-up phase:  **OIT: 18%** (16/88)  Control: not reported  Maintenance phase: not clear  Burks, 2012^17^ - **egg** :  Before maintenance:  **OIT: 13% (5/40)** (4 allergic reaction, 1 anxiety)  **Placebo: 6.7% (1/15)** (1 allergy symptoms)  During maintenance:  **OIT: 2.9% (1/35)** (allergic reactions) (no maintenance for placebo)  Overall: **15%** (6/40)  Escudero, 2015^18^ - **egg**  **OIT: 6.7% (2/30)** (persistent mild reactions)  **Control: 0/31**  Fuentes-Aparicio, 2013^19^ – **egg** :  **OIT: 7.5% (3/40)** (persistent GI symptoms, including a case of confirmed EOE)  **Control: 0/32**  Morisset, 2007^12^ – **milk** :  **OIT : 7.1% (2/28)**  **Control : 0/32** | **Follow-up:** ≥ 10 months after start of OIT  **Total discontinuations due to AEs or treatment failure: 9.6%** (27/280):  **Primary failures (stopped OIT during the 1^st^ week): 1.8%** (5/280) (3 extreme sensitivity, 2 parental or psychological reasons-not AE)  **Recurrent anaphylaxis: 5.7%** (16/280, 59% [16/27 of all]  **GI symptoms with peripheral blood eosinophilia: 2.9%** (8/280)  **Food aversion: 3.6%** (10/280, percent of patients who discontinued after 1^st^ week: 45% [10/22])  **Total discontinuations: 14%** (39/280) | **Follow-up:** median 6.5 (range 1-11) years after start of OIT  Over median FU of 6.5 years 24% (71/295) of patients discontinued OIT (data missing from 15% [44/295] of patients)  **GI symptoms** were the most common self-reported reason for discontinuation: **16%** (41/252), **58%** (41 of 71 discontinuations)  Other reasons: cutaneous (34/71, 48%), respiratory (24/71, 34%), anaphylaxis (22/71, 31% - **7.5% of 295**), oropharyngeal (21/71, 30%), food aversion (7/71, 10%), ocular (5/71, 7%), lack of progress (4/71, 6%), and miscellaneous (8/71, 11%). | **Follow-up**: ≥ 1 month after reaching maintenance and up to 8 years  Build-up phase  **Loss to FU:** 2.0% (6/270)  **Discontinuations, total**: 16% (42/270)  **ETR: 3.0%** (8/270, 19% of discontinuations)  **ELORS: 7.8%** (21/270, 50% of discontinuations)  **Other AEs: 1.9%** (5/270, 12% of discontinuations)  **Total discontinuations due to AEs: 13%** (34/270, 81% of discontinuations)  **Food aversion: 0.7%** (2/270, 4.8% of discontinuations)  **Other** (anxiety, burden of care, perception, sibling with OIT): **2.2%** (6/270, 14% of discontinuations)  Maintenance phase  **Loss to FU:** 15% (33/214)  **Discontinuations, total**: **12%** (25/214) of patients (- sub-group: patients treated for ≥ 3 years of maintenance: 11% [12/105])  **ETR: 1.4%** (3/214, 12% of discontinuations)  **ELORS: 0.5%** (1/214, 4.0% of discontinuations)  **Other AEs: 1.9%** (4/214, 16% of discontinuations)  **Total discontinuations due to AEs:** 3.7% (8/214, 32% of discontinuations)  **Food aversion: 5.1%** (11/214, 44% of discontinuations)  **Other** (perception, irregular dosing, failed SU): **2.8%** (6/214, 24% of discontinuations)  **Incidence of discontinuations:** 3.9/100 patient-years  **Discontinuations due to AEs in b**oth phases combined: 42/270=**17.5%** |

* At date of data cut-off, 15 patients continued to receive increasing amounts of cow milk (i.e., have not completed the build-up phase).

#### Allergens other than milk, egg and peanut

|  | **Nowak-Wegrzyn, 2019^24^ (RCT, N=46) -wheat** | **Kulmala, 2018^25^ (case series, N=100) -wheat** | **Elizur, 2019^26^ (CCT, N=73)-walnut** | **Barni, 2019 (case series, N=43) - hazelnut** | **Nachshon, 2019^28^ (CCT) N=75 - sesame** |
| --- | --- | --- | --- | --- | --- |
| **Age** | Median, 8.7 years; range, 4.2-22.3 | Mean 11.6 years, range, 6.1-18.6 | Mean 7.9 years OIT, 6.8 years control; range 4-20 | Mean 10 years, range: 5-16 | OFC or reaction within past year together with positive skin prick test (SPT) result and/or specific serum IgE (>0.35 kUA/L) |
| **Discontinuations** | 11/46 (24%) subjects discontinued the study:  1 subject in the low-dose VWG OIT group discontinued after completing the year 2 OFC, 6 subjects discontinued the study before week 52 and were counted as failures for the primary end point; 4 in the active VWG OIT arm (3 because of dosing-related symptoms and 1 because of participant’s decision) and 2 in the placebo arm (1 because of participant’s decision and 1 because of nonadherence). In addition, in the high-dose crossover group 2 subjects discontinued participation because of dosing symptoms (1 was given a diagnosis of both ulcerative colitis and EoE) and 1 because of nonadherence.  Discontinuation due to AEs in low-dose VWG during the build-up phase: 3/23 (13%) OIT vs 0 placebo  In maintenance phase: 1/19 (5.3%) | Total: 43% (43/100)  Build-up: 23/100 (23%) – dropouts due to severe reaction: 7.0% (7/100) (14 had severe reaction)  Maintenance phase 1 (3 months): 6.5% (5/77) – dropouts due to severe reaction: 1/77 (1.3%) (6 had severe reaction)  Follow-up (maintenance phase 2: 9 months): 21% (15/72) – dropouts due to severe reaction: 1/72 (1.4%) (6 had severe reaction)  Combining both maintenance phases: 26% (20/77) – dropouts due to severe reaction: 2/77 (2.6%) (6 had severe reaction)  34/ 43 (79%) had objective or objective and subjective symptoms and 8/43 (16%) only had subjective symptoms | one patient of those who had an ETR discontinued 1.8% (1/55) | Not reported | Build-up phase:  No discontinuation  Maintenance phase:  No discontinuation due to AEs (2 discontinued because of treatment burden, 2 because of food aversion) |

ETR: epinephrine-treated reaction; DBPCFC: double-blind, placebo-controlled food challenge; SCD: successfully consumed dose; VWG: vital wheat gluten

#### Assessment of the quality of evidence

|  |  | **Risk of bias** (Cochrane tool^57^ for interventional comparative studies; IHE tool^58^ for case series) | **Level of evidence (Oxford Centre for Evidence-based medicine) -** adapted using the approach of the European Academy of Allergy and Clinical Immunology^59^ |
| --- | --- | --- | --- |
| **Peanut** | **Interventional comparative studies (RCTs, CCTs):** meta-analysis (Chu, 2019^31^) | **Low** | Level I (Systematic reviews, meta-analysis, randomized controlled trials) |
|  | **Case series:** Wasserman, 2019^8^ | **Moderate** – 1 case series, main limit: retrospective study design |  |
| **Egg** | **Interventional comparative studies (RCTs, CCTs):** Burks, 2012^17^, Escudero, 2015^18^ Fuentes-Aparicio, 2013^19^ | **Moderate** – 3 RCTs: 1 low, 1 high, 1 unclear risk of bias | Level I (Systematic reviews, meta-analysis, randomized controlled trials) |
|  | **Case series** | **--** |  |
| **Milk** | **Interventional comparative studies (RCTs, CCTs):** Morisset, 2007^12^ | **High** – 1 RCT at high risk of bias | Level I (Systematic reviews, meta-analysis, randomized controlled trials) |
|  | **Case series**: Levy, 2014^14^ (clinical practice) | **Moderate** – 1 case series, main limit: retrospective study design |  |
| **Wheat** | **Interventional comparative studies (RCTs, CCTs)**: Nowak-Wegrzyn, 2019^24^ | **Low** – 1 RCT | Level I (Systematic reviews, meta-analysis, randomized controlled trials) |
|  | **Case series**: 1 prospective case series in research context (Kulmala, 2018^25^) | **Low** – 1 prospective case series |  |
| **Walnut** | **Interventional comparative studies (RCTs, CCTs)**: Elizur, 2019^26^ (CCT, N=73) | **High** – non-randomized control group,** unblinded | Level II (two groups, non-randomized studies) |
|  | **Case series**: NA | **--** |  |
| **Hazelnut** | **Interventional comparative studies (RCTs, CCTs)**:NA | **--** | -- |
|  | **Case series:** NA | **--** |  |
| **Sesame** | **Interventional comparative studies (RCTs, CCTs)**: Nachshon, 2019^28^ | **High** – non-randomized control group,*** unblinded | Level II (two groups, non-randomized studies) |
|  | **Case series**: NA | **--** |  |

### Long-term FU for safety and tolerability

| **LTFU of RCTs (with control arm)** | | | **Large observational studies (case series)** | | | |
| --- | --- | --- | --- | --- | --- | --- |
| **Jones, 2016^60^ – LTFU of Burks, 2012 (egg)^32^**  Diagnosis confirmed with OFC: no | **Hsiao, 2017^61^ – LTFU of Tang 2015 (peanut):^4^**  Diagnosis confirmed with OFC: no | **Meglio, 2017^62^ – LTFU of Meglio 2013 (egg)^37^**  Diagnosis confirmed with OFC: yes (DBPCFC) | **Manabe, 2019^77^ (Japan)* - egg, cow's milk, or wheat**  N=130  Age: 6-NR (children)  Diagnosis confirmed with OFC: yes (DBPCFC) | **Kauppila, 2019^15^ (Finland) – cow’s milk**  N=295  Age: 5-17 (median 7.5) years  Diagnosis confirmed with OFC: yes | **Elizur, 2016^16^ (Israel) - cow’s milk**  N=196  Age: >6, mean 10 years  Diagnosis confirmed with OFC: yes, for patients who did not have an anaphylactic reaction in the preceding year | **Nachshon, 2018**^7^ **(Israel)- peanut**  N=145  Age: ≥4 (median 5.8) years  Diagnosis confirmed with OFC: yes, for patients who did not have an anaphylactic reaction in the preceding year |
| **Patients followed:** All patients who started OIT or control therapy  **Follow-up**: Point 1: approx. 5 years after study enrolment; Point 2: 1 year later  **Completeness of follow-up**:  OIT: 85% (34/40); placebo: 73% (11/15) at both time points  Year 3 and 4 after initiation of OIT: 3 egg-related reactions, 2 in OIT-desensitized subjects including **1 ETR in a patient who had achieved SU** 19.4 months prior and reported unrestricted egg consumption. | **Patients followed:** All patients who started OIT or control therapy  **Follow-up:** Mean of 4.2 years (SD 0. 7) from treatment cessation  **Completeness of follow-up**:  77% (OIT: 24/31; placebo: 24/71) of patients  **No ETR, no anaphylaxis**  **Estimated number of reactions per 10 person-years:**  OIT: 1.1 (99.9 person-year of FU)  Placebo: 0.9 (102 person-years of FU) | **Patients followed:** All patients who started OIT or control therapy  **Follow-up:** Mean of 2.5 (SD 0.3) and 7 (SD 0.9) years after original study start  **Completeness of follow-up**: 2.5-year follow up: 19/20; 7-year follow-up: 18/20  **No ETR, no need for emergency care** | **Patients followed:** Patients who achieved a 2-week SU within 2 years after starting OIT  **Follow-up:** ≥ 1 year (median 3.6) since achieving a 2-week SU  **Completeness of follow-up**: 85% (108/130)  Adverse reactions in follow-up period among patients with follow-up:  **Any medication-treated symptoms**: 44/108 (41%) -  Egg: 17/61 (28%); Cow’s milk: 17/30 (57%); Wheat: 10/17 (59%)  **Any moderate or severe medication-treated symptoms**: 24/108 (22%)  **ETRs**: 2 (1 egg, 1 wheat)/108 (1.9%)  73% (32/44) patients reporting symptoms attributed their symptoms to co-factors such as exercise, fatigue or illness  59% (22/ 37) of patients with clear responses) experienced their first symptoms ≥2 years after 2-week SU | **Patients followed:** All patients who started OIT  **Follow-up**: median 6.5 years (range 1-11)  **Completeness of follow-up**:  83% (244/295) of patients  ETRs in the past year among the patients with follow-up:  Patients consuming ≥200 mL/d milk: 1.6% (2/122)  Patients consuming <200 mL/d milk: 5.6% (2/36)  *Patients consuming any milk: 2.5% (4/158)*  Patients avoiding milk: 8.9% (4/45)  P=0.09  Any milk-related side-effect in the past year among the patients with follow-up:  Patients consuming ≥200 mL/d milk: 37% (45/122)  Patients consuming <200 mL/d milk: 81% (29/36)  *Patients consuming any milk: 47% (74/158)*  Patients avoiding milk: 67% (30/45)  P=0.000  Among patients who consumed ≥200 mL/d milk, those with longer follow-up tended to have fewer side effects (P = 0.07) | **Patients followed:** Patients who reached full cow milk protein consumption  **Follow-up**: ≥ 6 months after completing up-dosing phase; median: 24.8 (range 6–41) months  **Completeness of follow-up**:  195/196 (99%) of patients  Adverse reactions during follow-up period**:**  **ETRs**: 13/195 (6.7%)  **Any AE** due to cow milk: 100/195 patients (51%)  Respiratory: 57/195 (29%)  GI: 17/195 (8.7%)  **Rate of AEs following completion of up-dosing:**  Month 6–15 months (n=29): 0.28/month  Month 15–30 months (n=39): 0.21/month  After > 30 months (n=28): 0.15/month  P < .01 | **Patients followed:** All patients who started OIT  **Follow-up:** ≥ 6 months after completing build-up phase; median: 18 (range 6–75) months  **Completeness of follow-up**:  142/145 (98%) of all patients  130/133 (98%) of patients reaching 3000 mg (n=113) or 300-2400 mg (n=20) PP at end of up-dosing  Adverse reactions during follow-up period among patients consuming 300-3000 mg PP :  **ETRs:** 2/130 (1.5%)  **Any objective reaction**: 12/130 (9.2%) |

*Included for this outcome event though N<150 for each allergen because of scarcity of long-term data after SU

#### ASSESSMENT OF THE QUALITY OF EVIDENCE

|  |  | **Risk of bias** (Cochrane tool^57^ for interventional comparative studies; IHE tool^58^ for case series) | **Level of evidence (Oxford Centre for Evidence-based medicine) -** adapted using the approach of the European Academy of Allergy and Clinical Immunology^59^ |
| --- | --- | --- | --- |
| **Peanut** | **Interventional comparative studies (RCTs, CCTs):** Hsiao, 2017^61^ | **High**- 1 LTFU on RCT, limitations: open-label and loss to follow-up | Level II two groups, non-randomized studies |
|  | **Case series:** Nachshon, 2018^7^ | **Moderate** – 1 case series, main limit: retrospective study design, single center |  |
| **Egg** | **Interventional comparative studies (RCTs, CCTs):** Jones, 2016^60^, Meglio, 2017^62^ | **High** – 2 LTFU on RCTs, limitations: open-label and loss to follow-up | Level II two groups, non-randomized studies |
|  | **Case series** | **--** |  |
| **Milk** | **Interventional comparative studies (RCTs, CCTs):** | **--** | Level III: one group non-randomized |
|  | **Case series**: Kauppila, 2019^15^, Elizur, 2016^16^, Manabe, 2019^77^ | **Moderate** – 3 case series, main limit: retrospective study designs, single center |  |
| **Wheat** | **Interventional comparative studies (RCTs, CCTs)**: NA | **--** | Level III: one group non-randomized |
|  | **Case series**: Manabe, 2019^77^ | **Moderate** – 1 case series, main limit: retrospective study designs, single center |  |
| **Walnut** | **Interventional comparative studies (RCTs, CCTs)**: NA | **--** | -- |
|  | **Case series**: NA | **--** |  |
| **Hazelnut** | **Interventional comparative studies (RCTs, CCTs)**:NA | **--** | -- |
|  | **Case series:** NA | **--** |  |
| **Sesame** | **Interventional comparative studies (RCTs, CCTs)**: NA | **--** | -- |
|  | **Case series**: NA | **--** |  |

### Eosinophilic esophagitis

| **Meta-analyses** | | **Large case series (N> 150)** | | | | |
| --- | --- | --- | --- | --- | --- | --- |
| **Chu, 2019^31^ – peanut** | **Lucendo, 2014^78^ – chicken’s egg, cow’s milk, peanut and others** | | **Goldberg, 2017^79^ (Israel) - milk, peanut, egg, sesame**  N=794  Age: median 83 months (IQR 60-126)  Diagnosis confirmed with OFC: yes, for patients who did not have an anaphylactic reaction in the preceding year | **Wasserman, 2019^8^ (USA) – peanut**  **N=270**  **Age: 4-18 (mean: 8.1) years**  **Diagnosis confirmed with OFC: no** | **Kauppila, 2019^15^ (Finland) – cow’s milk**  **N=295**  **Age: 5-17 (median 7.5) years**  **Diagnosis confirmed with OFC: yes:** | **Soller, 2019^9^ (Canada) – peanut**  **N=270**  **Age: 0.75-5.9 (median 1.9, IQR : 1.25-2.75) years**  **Diagnosis confirmed with OFC: for 31% of patients** |
| 3 events of EOE occurred in 3 RCTs; another 2 RCTs reported explicitly that no EOE occurred (N=719), all in the OIT group – *no statistical analysis performed* | Meta-analysis of 9 studies (8 retrospective case series, 1 RCT, published up to March 2014) that reported on a total of 708 patients receiving OIT (including baked milk introduction), indicated that 2.7% of patients undergoing OIT newly developed EOE (95% CI 1.7% to 4.0%). | | **Recurrent GI symptoms (abdominal pain and/or vomiting) independent of the timing to dose administration:** 8.2% (65/794)  Milk: 9.0% (55/614)  Peanut: 6.9% (9/130)  Egg: 2.4% (1/41)  39% (25/65) of cases occurred within the first month and 86% (56/65) within the first 3 months of OIT  69% (45/65) resumed OIT and 42 did not redevelop symptoms*  No dysphagia, no food impaction.  3 patients underwent biopsy showing increased esophageal eosinophilic counts  In all cases in which the dosage was reduced or stopped, symptoms subsided, and peripheral eosinophil counts decreased.  Patients with recurrent GI symptoms had higher peripheral eosinophil counts at baseline (P=0.046) and higher increases in peripheral eosinophil counts during OIT (P<0.001) compared to other patients, but did not differ in age and sex. | **ELORS:** EOE-like OIT-related syndrome: episodic vomiting occurring > 2 hours after dosing  Build-up phase:  Proportion of patients with **ELORS**: **14%** (**37**/270) (documented increase in peripheral blood eosinophils: 4.8% (13/270), only 16 of 37 tested)  Biopsies were performed in 2/37 patients (results not reported); 18/37 patients were treated with a PPI for 1-4 weeks and 1/37 with an oral corticosteroid for < 14 days  Proportion of patients with ELORS who:  -discontinued: 57% (21/37)  -reached target maintenance dose: 35% (13/37)  -Discontinued for other reasons or transferred care: 8.1% (3/37)  None of the 35 patients with ELORS with follow-up (2 transferred care) had persistent symptoms or required prolonged therapy.  Maintenance phase:  Proportion of patients with **ELORS** during maintenance phase: **0.5%** (1/214) –discontinued OIT  Incidence of **ELORs** during maintenance phase: 0.16/100 patient-years | One patient with vomiting and failure to thrive underwent endoscopy, which was negative for EOE; the examination was performed after OIT discontinuation while the patient was on a milk‐avoidance diet. | 1.1% (3/270) of patients experienced symptoms suggestive of EoE, 1 biopsy performed (no EoE)  An additional case of EoE identified incidentally during a biopsy to rule out celiac disease (the EoE persisted despite stopping OIT) |

* Goldberg (2019^80^) reported details on the management and the treatment course of these patients: the cumulative daily dose was reduced by a median of 50% in 66% (43/65) of patients, dose increases were deferred in 3.1% (2/65) of patients, or treatment was temporarily suspended in 28% (18/65) of patients. Symptoms and eosinophilia abated on dose modification, allowing for resumption of dose increases for 34 patients or reinitiation of treatment for 9 patients. OITIGER reoccurred 18.5% (10/54) of patients and resolved after further dose modification. In long-term follow-up (>3-26 months), patients with OITIGER had a higher OIT failure rate (P=.004) and were less likely to reach full desensitization (P < .001).

| **Smaller case series** | |
| --- | --- |
| **Echeverria-Zudaire, 2016^81^ (Spain) - milk (N=97) or egg (31)**  **N=128**  **Age: children**  **Diagnosis confirmed with OFC: yes (open)** | **Gomez Torrijos, 2017^82^ and Rodriguez, 2014^83^ (Spain) – milk**  **N=57**  **Age: children**  **Diagnosis confirmed with OFC: NR** |
| **Biopsy-confirmed EOE: 6.3% (8/128)**  6 patients developed EOE at a median of 29 months (range 15-48) after OIT(all with cow’s milk); 2 developed EOE during OIT (egg and milk plus egg)  Most of the 8 patients were males between 3 and 14 years of age.  **EOE with oesophageal involvement only**: 4.5% (6/128)  5/6 continued OIT with PPI (n=3) or PPI plus fluticasone (n=2); 4/5 underwent follow-up endoscopy: 3 histological remission, 1 continued EOE.  Histological remission was confirmed in the patient who discontinued OIT.  **EOE - plus duodenal or colon involvement**: 1.6% (2/128)  Both discontinued OIT and underwent follow-up endoscopy which showed histological remission in both cases | **Biopsy-confirmed EOE: 5.3% (3/57**) of patients treated with OIT for cow milk allergy  All three patients were males and developed symptoms (including dysphagia) after reaching complete desensitization and/or while on maintenance. All patients discontinued milk intake, redeveloped sensitization and started avoiding milk.  Biopsies performed 6-24 months after starting avoidance showed resolution of EOE. |

### Impact of patient characteristics on safety outcomes

- Most OIT studies enrolled children and adolescents starting from age 4 to 7 years, with median/mean ages in the range of 6 to 12 years (Anagnostou, 2014;^1^ Bird, 2018;^5^ Burks, 2012;^32^ Elizur, 2016;^16^ Escudero,2015;^18^ Fuentes-Aparicio, 2013;^19^ Kauppila, 2019;^15^ Levy, 2014;^14^ Longo, 2008;^10^ Martin-Munoz, 2019;^20^ Nachshon, 2018;^7^ Reier-Nilsen, 2019;^3^ Vickery, 2018^6^ and Wasserman, 2019^8^). Three studies enrolled peanut-allergic children starting from a younger age, three years (Blumchen, 2019;^2^ Wasserman, 2014^56^) or one year (Tang, 2015^4^). One study enrolled children for milk or egg OIT starting from the age of one year (Morisset, 2007^12^).
- **Older children, adolescents and adults**
  - No association between baseline age and safety outcomes in large case series of children and adolescents (N 130 to 295) (Wasserman 2019^8^, Elizur, 2016;^16^ Kauppila, 2019;^15^ Levy 2014^14^)
  - A double-blind, placebo-controlled RCT included 55 adults (18 to 55) and 169 adolescents (age 12 to 17) with peanut allergy (Burks, 2018;^70^ Vickery 2018^6^). There was no apparent difference between age groups in the proportion of patients with treatment-emergent hypersensitivity adverse events.

| **Age range (years)** | **N (OIT)** | **N (placebo)** | **Proportion of patients experiencing event OIT versus placebo** | | | |
| --- | --- | --- | --- | --- | --- | --- |
|  |  |  | **Treatment-emergent allergic hypersensitivity adverse events** | **Severe adverse event** | **Serious adverse events** | **Anaphylaxis/Systemic allergic reaction** |
| 4 to 11 | 238 | 89 | 86.1% versus 69.7% | 4.3% vs 0.8% | 2.4% vs 0.8% | 14.2% vs 3.2% |
| **12 to 17** | **134** | **35** | **89.6% versus 68.6%** |  |  |  |
| **18 to 55** | **41** | **14** | **87.8% versus 78.6%** | **4.9% vs 7.1%** | **4.9% vs 7.1%** | **19.5% vs 7.1%** |

- - Double-blind, placebo-controlled RCT of adolescents with peanut allergy (mean age 15 years, range 12-18; age at diagnosis: 3 years) (Fauquert, 2018^55^): Peanut (or placebo) capsules were ingested daily over 24 weeks with increments every 2 weeks from 2 to 400 mg of peanut protein. **Five severe multi-system reactions (grades 3a and 3b) occurred in 4 OIT patients (19%= 4/21) and in none (0) in the placebo group.**
  - Case series of 23 adults with OFC-confirmed IgE-mediated allergies who were treated with OIT (10 milk, 9 peanut, 4 egg) (Mantyla, 2018^71^): The median period of OIT was 515 days. **ETRs: 17% (4/23); emergency room treatments: 13% (3/23).**
- **Toddlers and pre-school children:**
  - Vickery et al (2017^68^) randomized 37 preschool children aged 9 to 36 months with OFC-confirmed peanut allergy to OIT with a maintenance dose of 300 or 3000 mg peanut protein per day. One ETR occurred in the high dose group (5.9%, 1/17) and none in the low-dose group. **There were no treatment-related severe adverse events.**
  - Martorell et al (2011^11^) randomized 60 children aged 24 to 36 months with OFC-confirmed milk allergy to OIT or avoidance. **Two ETRs occurred in 2 children in the OIT group (6.7%, 2/30); no severe reactions occurred.**
  - Soller et al (2019,^9^ prospective multi-center study) reported that of 270 pre-school children (age 0.75-5.9 years, median 1.9 years) undergoing peanut OIT 36% (98/270) experienced grade 1 (mild) symptoms, 31% (84/270) grade 2 (moderate), and 0.4% (1/270) grade 4 (severe) symptoms graded according to the World Allergy Organization Subcutaneous Immunotherapy Systemic Reaction Grading System^74^). Eleven patients (4.1%, 11/270) experienced a total of 12 ETRs during build-up (11 for grade 2 reactions and 1 for a grade 4 reaction). Half of the ETRs (6) occurred in the clinic during up-dosing and the other half at home. Overall, a total of 0.029% of doses during build-up were associated with an ETR.

### Impact of baseline food allergy-related parameters on safety outcomes

Inclusion of patients with a history of severe reactions in OIT studies:

- Among 13 RCTs with N> 50, 6 excluded patients with a history of severe anaphylaxis (Burks, 2012;^32^ Escudero, 2015;^32^ Martorell, 2011;^32^ Tang, 2015;^4^ Bird, 2018;^5^ Vickery; 2018;^6^ Sampson, 2019^63^), which was commonly defined as hypotension, neurological and/or respiratory compromise. Other RCTs (Longo, 2008,^10^ Martin-Munoz, 2019a,^20^ Reier-Nilsen, 2019a^3^) either did not list anaphylaxis among their exclusion criteria or, additionally, specifically stated that they did not exclude children with a history of severe allergic reactions (Blumchen, 2019;^2^ Anagnostou, 2014^2^). (For another 2 studies [Fuentes-Aparicio, 2013, Morisset, 2007], inclusion of patients with severe anaphylaxis was not clear.
  - In peanut RCTs, inclusion criteria for the minimum serum peanut-specific IgE level ranged from 0.35 to15 kU/L; there was no maximum sIgE level specified (Chu, 2019).^31^
- Large case series (clinical practice) generally did not exclude patients with a history of anaphylaxis; two of them explicitly state that no patient was excluded because of the severity of their previous reactions or their sIgE levels (Wasserman, 2018;^94^ Wasserman, 2014^56^).

Outcomes of RCTs with N≥ 50 that included children with a history of severe reactions:

- **Anagnostou, 2014^2^** (**peanut** RCT, N=99, baseline worst clinical reaction WAO score grade 3 or 4: control 22.5% vs active 8.1%): ETRs occurred after 0.01% of doses (1 participant); OIT vs placebo: 2.0% (1/49) vs 9 (0/50). There were no SAEs.
- **Blumchen, 2019^2^** (**peanut** RCT, N2=62: 31 OIT, 31 control): At baseline, 53% of children in the OIT group and 58% in the control group had a history of severe allergic reactions to peanut (grade IV or V^72^). The median sIgE was 81.5 kU/L (range 0.57-624 kU/L) and the median maximum tolerated single dose at the initial OFC was 30 mg peanut protein (range 1-3,000 mg). Wheezing was the only objective symptom reported significantly more often in the OIT group (8 events in 6 patients) than in the placebo group (1 patient; P=.045). There were no ETRs; the rate of serious AEs was 10% (3/30) in the OIT group and 16% (5/31) in the placebo group.
- **Longo, 2008^10^** (**milk** RCT, N=30 OIT, N=30 control): Included children with a baseline sIgE level > 85 kUA/L who had a positive history of at least one severe allergic reaction (defined as class 4 and 5 by Clark’s classification) after accidental exposure to milk or dairy products requiring emergency treatment. In the OIT group, almost all patients had allergic reactions; 4 ETRs occurred in 4 OIT patients (13%) during the initial hospital-based rush phase and 1 ETR (3.3%) during the home dosing phase. During the home dosing phase, 2/30 (6.7%) children attended the emergency department. In the control group, 6/30 (20%) children had mild adverse reactions caused by accidental exposure to milk.
- **Martin-Munoz, 2019a^20^ (egg,** RCT**):**  (N=88 OIT, 25 controls) ETRs: 8.0% OIT vs Control: NR; No SAEs
- **Reier-Nilsen, 2019a^3^** (**peanut**, N=77: 57 OIT, 20 control): At baseline, 79% of children had a history of anaphylaxis to peanut and all children reacted with anaphylaxis during the baseline DBPCFC. During up-dosing, 11/57 (19%) of children in the OIT group experienced 11 anaphylaxis events (classified as moderate), including 6 ETRs; the control group did not experience any anaphylactic reactions to peanut.

Correlations between baseline parameters and OIT safety and tolerability outcomes

**LARGE CASE SERIES**

| **Baseline parameter** | **Outcome and correlation** | **Reference** |
| --- | --- | --- |
| **History of anaphylactic reaction** | Associated with **adverse reactions** after the completion of treatment (median follow-up 24.8, range 6-41) months after completion of the build-up phase (OR=2.1, P=.033) | Elizur, 2016^16^ (milk, N=197) |
|  | History of anaphylaxis among the following sub-groups by outcome:   - Continued treatment: 39% (38/97) - Stopped treatment: 71% (10/14), P=0.04 | Nachshon, 2018^7^ (peanut, N=111) |
|  | In multivariate analysis, history of anaphylaxis was **not** associated with ELORS or ETRs | Wasserman, 2018^94^ (peanut, N=270) |
| **Specific IgE serum levels** | In logistic regression analysis, milk-related anaphylaxis after buildup was related to milk sIgE before OIT; for every additional sIgE doubling, the **risk of anaphylaxis** increased by 60% (OR 1.6) (P=0.000) | Kauppila, 2019^15^ (milk, N=296) |
|  | Higher sIgE increased the risk of **ELORS** (P <.001) and of **ETRs** (P=0.19) occurring during escalation | Wasserman, 2018 (peanut, N=270) |
|  | In a multivariable logistic regression model those with a higher baseline IgE were more likely to **drop out** during P-OIT buildup (odds ratio [OR], 1.03; 95% CI, 1.01-1.05) and were more likely to receive epinephrine (**ETR**) (OR, 1.03; 95% CI, 1.011.06) | Soller, 2019^9^ (peanut, N=270) |
| **SPT wheal size** | In a multivariable logistic regression model, patients with a higher baseline SPT were more likely to receive epinephrine (**ETR)** (OR, 1.35; 95% CI, 1.051.75) | Soller, 2019^9^ (peanut, N=270) |
|  | **No** significant difference between those who continued or stopped treatment (P=0.62) | Nachshon, 2018^7^ (peanut, N=111) |
|  | **Not** associated with occurrence of adverse reactions following completion of OIT (P=0.087) | Elizur, 2016^16^ (milk, N=197) |
| **Maximum tolerated dose of food allergen** | MITD not associated with occurrence of adverse reactions following completion of OIT (P=0.087) | Elizur, 2016^16^ (milk, N=197) |
|  | Starting dose: median 120 mg cow milk protein; range (10-7200 mg). ETRs were significantly more frequent in with a lower maximal tolerated starting dose (P <.0001). | Levy, 2014^14^ (milk, N=280) |

**RCTs, CCTs AND SMALLER CASE SERIES**

| **Baseline parameter** | **Outcome and correlation** | **Reference** |
| --- | --- | --- |
| **Specific IgE blood levels** | Patients who dropped out had higher egg sIgE serum antibody levels (P < 0.05). | Martin-Munoz, 2019a^20^ (RCT **egg**, N=76 OIT, 25 control) |
|  | Higher baseline sIgE levels were associated with OIT discontinuation or persistent reactions to OIT dosing. Optimal predictive cut-off levels were: egg-white sIgE: 9.41 kU/L; ovalbumin-sIgE: 6.49 kU/L and ovomucoid-sIgE: 8.85 kU/L. | Vazquez-Ortiz, 2014^54^ (CCT **egg**, N=50 OIT, N=32 control) |
|  | Fewer patients with lower sIgE levels (<3.5 kU/L) experienced adverse events (6/10, 60%) compared to 13/17 (76%) of patients with higher sIgE levels >3.5 kU/L (P=NR). | Garcia-Ara, 2013^43^ (CCT, **milk**, N=36 OIT; N=19 control) |
|  | Cox proportional hazards multivariate regression model identified sIgE ≥50 kU/L (HR 2.59, 95% CI 1.4-4.78, P=0.002) as independent risk factor for persistence of allergic reactions. | Vazquez-Ortiz, 2013^84^ (case series, **milk**, N=81) |
| **SPT** | Peanut SPT was the only significant predictor of the rate of GI AEs, both before and after adjusting for sex, age, asthma, log peanut-specific IgE, atopic dermatitis, and AR. Rates of GI AEs increased 1.8-fold (95% CI: 1.4, 2.4, p-value: <0.001) for every 5 mm increase in SPT size. | Virkud, 2017^85^ (retrospective pooled analysis of 3 RCTs, **peanut**, N=104) |
| **Maximum tolerated dose of food allergen** | Patients who dropped out had lower baseline threshold response dose (P < 0.05). | Martin-Munoz, 2019a^20^ (RCT **egg**, N=76 OIT, 25 control) |

### Impact of baseline asthma status on safety outcomes

In many interventional (Bird, 2018;^5^ Blumchen, 2019;^2^ Longo, 2008;^10^ Martin-Munoz, 2019;^20^ Reier-Nilsen, 2019;^3^ Tang, 2015;^4^ Vickery, 2018^6^) and in some observational (Levy, 2014;^14^ Nachshon, 2018;^7^) studies severe and/or poorly controlled or unstable asthma was an exclusion criterion for OIT.

**Asthma-related outcomes in RCTs with N>50:**

- Martin-Munoz, 2019a^20^ (Spain – **egg**, age 6-9 years, N=101, asthma 30/101 [30%], severe or uncontrolled asthma at baseline excluded): Among all patients who started OIT (N=88) 8 (9.1%) withdrew during the build-up phase because of uncontrolled asthma.
- Blumchen, 2019 (Germany– **peanut**, age 3.2-17.8 years, N=62, uncontrolled asthma excluded, asthma: 65% placebo, 42% OIT) : After the course of OIT, no difference was found with respect to newly diagnosed atopic diseases (bronchial asthma, atopic dermatitis, allergic rhinoconjunctivitis) or worsening established atopic diseases at baseline.
- Vickery, 2018^6^(**peanut**, poorly controlled asthma excluded, 53% of patients had asthma at baseline): Asthma exacerbation was recorded as an SAE in 2 OIT patients (4-17 years, N=372, 0.54%) and no placebo (N=124) patient.

**Correlations between baseline asthma status and OIT safety / tolerability outcomes in large case series**

| **Outcomes and Correlations** | **Proportion of patients with asthma** | **Reference** |
| --- | --- | --- |
| Compared to patients without asthma (N=93), patients with asthma (N= 101) had **more ETRs** during up-dosing (61% vs 34% of patients, mean ETRs per patient: 1.1 [SD 1.2] vs 0.4 [SD 0.7], P < .001) and at home (26% vs 4.2% of patients, mean ETRs per patient: 0.4 [SD 0.8] vs 0.1 [SD 0.5], P < .001). | 101 patients with asthma, 93 no asthma | Elizur, 2015^73^ (**milk**, N=194) |
| During the initial 10-day hospital-based “rush” phase, there was a **significant association** between the incidence of moderate to severe reactions and history of asthma (P=0.01).  During OIT dosing phase at home, there was **no significant correlation** between the presence of asthma and/or viral wheezing and the total number of reactions or reactions requiring nebulised epinephrine. | asthma: 66%, viral wheezing: 34% | Barbi, 2012b^86^ and Barbi, 2012a^87^ (**milk,** N=132) |
| History of intermittent asthma **increased the risk** of **ETRs** (P = .035) and **ELORs** (P = .014) during dose escalation. | asthma: persistent: 43%, intermittent: 21% | Wasserman, 2018^8^ (**peanut**, N=270) |
| While asthma was not significant in predicting rates of AEs overall, the presence of asthma significantly increased AE rates by 2.3 times during maintenance (P=0.03) | asthma: 44% | Virkud, 2017^85^ (retrospective pooled analysis of 3 RCTs, **peanut**, N=104) |
| 3 cases (2.3% of 130) of **life-threatening anaphylaxis** all in **teenage** boys with persistent asthma under suboptimal control, **high milk- or egg-specific IgE levels**, and **risk-taking behaviors**, including poor  compliance to OIT and/or to asthma management plans | 35% of patients (45/130) were on steps 3 to 4 of asthma treatment and 10% were adolescents (13-18 years old) | Vazquez-Ortiz, 2014^88^ (**egg, milk,** N=130) |

## IMPACT ON QUALITY OF LIFE

| **Study – food allergen**  **Study design** | **OIT protocol variables** | **PRO data collection** | **Number and age of patients enrolled** | **PRO Results (n: number of patients with valid responses)** | **Factors that influenced QoL outcomes** | **Main findings and study limitations** |
| --- | --- | --- | --- | --- | --- | --- |
| Anagnostou 2014^1^ - peanut  Crossover, open-label RCT | Target dose: 800 mg/d PP  Up-dosing interval: 2-3 weeks  Length of build-up phase: 26 weeks | **FAQLQ-PF** at end of OIT (26 weeks) – parent proxy | N=99 (49 OIT, 50 control)  Age: 7–16, median 12.4  FAQLQ-PF completed by parents of children of 7-12 yrs old | Median (range) change in **FAQLQ-PF** score from baseline to post-treatment:  OIT: (n=19): -1.61 (-4.87 to 0.24), P<0.001 (Wilcoxon signed rank test)  Control after crossing over to OIT (n=20): -1.41 (-4.83 to 1.38) | NR | OIT was associated with improvement in the parental FAQLQ-PF total score compared to baseline.  Limitations:  No comparator  Only parent report (no children),  Measured at one time point only  No information about the different domains of QoL |
| Dunn Galvin, 2018^89^, Tang, 2015^4^ – peanut  DBPCRT | Target dose 2000 mg/d PP  Up-dosing interval: 2 weeks  Length of build-up phase: 8 months  Maintenance dose and duration: 2000 mg/d PP for 10 months | **FAQLQ-PF** and  **FAIM** (– parent proxy) at:  T0 (baseline)  T1 (end-of treatment, 18 months from T0 and before assessment of SU)  T3 (3 months post-treatment)  T4 (12 month post-treatment) | N=62 (31 OIT, 31 placebo)  Age: 1–10, mean 6 | Mean (95% CI) change in **FAQLQ-PF total score** from T0 to:   \|  \| OIT \| Placebo \| \| --- \| --- \| --- \| \| End of treatment (T1) \| No statistically significant improvement \| \| \| T3: 3 months after T1 \| (n=24): +0.8 (0.001 to 1.7), P=0.05* \| (n=27): 0.4 (-0.3 to 1.1), P=0.3 \| \| T4: 12 months after T1 \| (n=20): +1.3 (0.4 to 2.1), P=0.005* \| (n=22): -0.6 (-0.8 to 0.7), P=0.8 \|   Improvement in FAQLQ-PF score by ≥0.5 (MCID) 3 months post-treatment (T3): OIT: 77%; Placebo: 34%; Absolute risk reduction: 42.2%; NNT = 2.3 (95% CI 10 to 2)  Improvement (mean difference from baseline) by subscale:   \|  \| T3 \| T4 \| \| --- \| --- \| --- \| \| FA \| 1.1, P = .003* \| 1.4, P = .001* \| \| SDL \| 0.9, P = .09 \| 1.2, P = .005* \| \| EI \| 0.6, P = .1 \| 1.1, P = .01* \|   Mean (95% CI) change in **FAIM score** from T0 to:   \|  \| OIT \| Placebo \| \| --- \| --- \| --- \| \| End of treatment (T1) \| (n=29): +0.8 (0.4 to 1.3), P=0.002* \| (n=28): -0.2 (-0.5 to 0.2), P=0.3 \| \| T3: 3 months after T1 \| (n=24): +0.5 (0.04 to 1.0), P=0.03* \| (n=27): -0.3 (-0.6 to 0.04), P=0.02 \| \| T4: 12 months after T1 \| (n=20): +0.4 (0.1 to 0.8), P=0.04* \| (n=22): -0.13 (-0.4 to 0.1), P=0.3 \|   Improvement (mean difference from baseline) by subscale:   \| **Chance of:** \| **T3** \| **T4** \| \| --- \| --- \| --- \| \| Severe reaction, FAIM-2 \| 1.0, P=.03* \| 0.8, P=.04* \| \| Dying from allergen ingestion, FAIM 3 \| 1.0, P=.1 \| 1.2, P=.01* \| \| Accidentally eating allergen, FAIM-1 \| 0.9, P=.02* \| 0.7, P=.04* \| \| Minimal improvement, FAIM-4 \| P=0.3 \| \| | In the OIT group, 23 patients attained SU, 5 patients failed to achieve SU and 3 withdrew.  Patients who did not achieve SU (n=NR) showed no improvement in FAQLQ-PF scores at 3 or 12 months post-treatment (Mean Difference T0-T3 0.58, t = 1.05, P =0.4; Mean Difference T0-T4 0.2, t =0.23, P =0 .8).  Patients who achieved SU (n=NR) reported a significant improvement in FAQLQ-PF scores across all subscales at 3 (T3) and 12 (T4) months post-treatment (Mean Difference T0-T3 1.3, t = 2.8, P = .01; Mean Difference T0-T4 1.8, t = 2.7, P = .001). | In a placebo-controlled trial, OIT was associated with clinically-important improvement of parental FAQLQ-PF scores compared to baseline 3 months and 12 months after the end of treatment, while there was no change in the placebo group.  Improvements were recorded in each of the 3 domains of the FAQLQ-PF, with the greatest improvement in the domain “food avoidance”.  Improvements were positively associated with achieving SU.  Limitations:  *Incomplete FU: by 12 months post-treatment, FAQLQ-PF data is only available from approx. 2/3 of the patients originally enrolled.  *No statistical analysis to compare change in FAQLQ-PF scores between the OIT and the placebo group.  *Only parental report (no children)  *Placebo-controlled (up to T1?): Subjective impact of OIT may not be fully captured in the absence of assurance of successful treatment |
| Blumchen, 2019^2^ - peanut  DBPCRT | Target dose: 125-250 mg PP  Build-up interval: 2 weeks  Build-up duration: up to 14 months  Maintenance dose and duration: median: 125 mg/d for 8±2 weeks | **FAQLQ-PF** (for mothers of children age 3-12) at a median of 9.5 weeks (IQR, 5-15.3) after final OFC  **FAQLQ-CF** (for children age 8-12) at a median of 11 weeks (IQR, 7-16) after final OFC | N=62 (31 OIT, 31 placebo)  Age: 3.2-17.8, median 6.8  FAQLQ-PF: OIT: 27, placebo: 21  FAQLQ-CF: OIT: 11, placebo: 9  BOT: OIT : 50 mothers of children (3-12 years), 21 children (8-12 years) | **FAQLQ-PF (mothers)**  Total score: median change from baseline not statistically significant in both OIT (n=20) and placebo (n=18) groups  No statistically significant change in all sub-scales  No statistically significant difference in changes between OIT and placebo.  **FAQLQ-CF (children age, 8-12 years)**  Total score: median change from baseline statistically significant in OIT group (n=9): -1.0 [IQR -2.7 to -0.5]; not statistically significant in placebo group (n=8): -0.1 [IQR-1.2-0.7], but difference between OIT and placebo not statistically significant (P=0.1)  By subscale, statistically significant improvement from baseline for:   - RAE (-2.0 [IQR -3.3 to -0.9]) - EI (-1.8 [IQR -2.8 to -0.9]), and - AA (-1.9 [-3 to -0.1]),   but not for DR.  Changes statistically different from placebo only for “risk of accidental exposure” and “emotional impact” (P=0.02 for both) | Not reported | In a placebo-controlled RCT, at approx. 3 months post-treatment there was no significant change of parental FAQLQ-PF scores compared to baseline or to placebo. However, children (age 8-12) in the OIT group reported clinically important and statistically significant improvements from baseline in the FAQLQ-CF total score and in all domains, except “dietary restrictions”. There were no significant changes from baseline in the children’s placebo group; differences between the two groups were not statistically significant, except for “risk of accidental exposure” and “emotional impact”.  Limitations:  *Incomplete FU  *Small sample size, particularly for the children (20 in total)  *Outcomes measured at one time point only  *Placebo-controlled: Subjective impact of OIT on QoL may not be fully captured in the absence of assurance of successful treatment |
| Reier-Nilsen, 2019b^90^ and 2019a^3^ - peanut  RCT-open label | Target dose: 5000 mg PP  Build-up interval: 2 weeks  Build-up duration: 50-78 weeks  Maintenance dose and duration planned: 5000 mg/d for 36 months; observed: mean 3322 mg PP (range 350 to 5000) | **PedsQL 4.0** (parents and children) at enrolment (Y0), after 1 year (end of up-dosing) (Y1) and after 2 years (Y2) of OIT  **FAQL-PB** (parents) | N=77 (57 OIT, 20 control)  Age: 5-15, median 10.1 OIT and 8.9 control | Mean (95% CI) **PedsQL 4.0** scores from baseline to Y2:   \| **OIT (n=39)** \| **Control (n=20)** \| **Between groups** \| \| --- \| --- \| --- \| \| **Children** \|  \|  \| \| from 82.1 to 86.7: +**4.4** (0.5, 8.3) P<.0001* \| from 83.4 to 82.2: **-0.9** (-7.9, 6.11), P=0.8 \| P= 0.12 \| \| **Parents** \|  \|  \| \| from 78.7 to 83.7: **+9.3** (4.3, 14.3) P<.0001* \| from 81.7 to 82.1: **+0.4** (4.3, 14.3), P=0.9 \| P=0.02* \|   **Parents’ FAQL-PB** improved significantly among both the OIT-group by -9.9 (95% CI -14.6, -5.3) (P<0.0001) and the control group by -9.4 (-15.3, -3.6) (P=0.004) from Y0 to Y2, with no significant difference between groups (P=0.57) | In multivariate robust regression analysis, none of the factors examined (age, gender, maintenance dose, perceived burden of GI AEs and perceived burden of taste/amount) were associated with a change in PedsQL4.0 (child), PedsQL4.0 (parent) or FAQL-PB (parents) scores. | Mean generic PedsQL 4.0 scores improved significantly from baseline to Y2 in both children and parents, while no significant difference was seen in controls. Compared to controls, the improvement was significantly different among parents only.  Limitations:  *Incomplete FU: Data not available for 32% (18/57) of the children who discontinued OIT  *Small sample size  *Children completed a generic QoL instrument only  *No information about the different domains of QoL |
| Epstein Rigbi, 2019^91^ - milk, peanut, egg, sesame, or tree nuts  Prospective cohort study | Target doses (mg protein): Milk: 7,200; egg: 12,000; peanut: 3,000; sesame: 5,000; tree nuts: 4,000  4 weeks (1 month)  Planned: 8 months; observed: mean 7.8 (SD 5.7) months  Maintenance (mg protein): Peanut, sesame, and tree nuts: 1200; milk: 3600; egg: 6000 | **FAQLQ-PF** (parents) at OIT initiation, mid up-dosing (4 months of OIT), after reaching maintenance (end of treatment), and 6 months into maintenance | N=191 (consecutive patients initiating OIT), 48 control group (matched for age and allergenic foods)  Age: 4-12, mean 6.3 | Mean scores in 175 (of 191, 92% follow-up) patients (158 had reached full, 14 partial maintenance dose, 3 discontinued [failed OIT])   \| **FAQLQ-PF** \| **OIT start** \| **Reaching maintenance or treatment cessation** \|  \| \| --- \| --- \| --- \| --- \| \| **Total score** \| 3.69 \| 3.19 \| P < .001* \| \| **EI** \| 3.66 \| 3.32 \| P=0.001* \| \| **FA** \| 3.90 \| 3.32 \| P<0.001* \| \| **SDL** \| 3.50 \| 2.94 \| P<0.001* \|   No significant changes in the control group (n=48) between 2 time points (mean interval 13.3 [SD11.2] months) in any of the domains or in the total score.  The 14 patients who reached a partial maintenance dose (150-2700 mg protein) experienced a significant improvement in mean FA (P=0.001), SDL (P=0.036), and total (P=0.015), scores, but not for EI (P=0.12) from start of OIT to maintenance. The 3 patients who failed OIT reported no significant change from the start of OIT to treatment cessation (range, 2.6-4.3 months).  Among 85 patients (of 96 eligible for mid-point assessment, 88.5% follow-up) who completed the FAQLQPF at mid up-dosing, a statistically significant improvement was seen between the start of OIT and mid-up-dosing for FA, SDL, and the total score but not for the EI domain:    Among the subset of patients whose FAQLQ-PF total score deteriorated (increased by > 0.5) from baseline to mid up-dosing (n=NR), FAQLQ-PF scores returned to near-baseline upon reaching maintenance.  Mean FAQLQ-PF scores among 93 OIT patients with 6-months of maintenance (of 105, 88.6% follow-up)   \|  \| **Baseline** \| **Reaching maintenance** \| **6 months of maintenance** \| \| --- \| --- \| --- \| --- \| \| **Total score** \| 3.55 \| 3.23 \| 2.55 \| \| **EI** \| 3.55 \| 3.37 \| 2.87 \| \| **FA** \| 3.78 \| 3.32 \| 2.55 \| \| **SDL** \| 3.33 \| 2.99 \| 2.28 \|   Significant differences were found for FA, SDL, and total score between start and maintenance and for all domains from maintenance to follow-up.  The 14 patients reaching partial maintenance reported had worse FAQLQ-PF scores at baseline and reported significant improvement in the FA (P=.001) and SDL (P=.036) domain and the Total score (P=.015) | In linear regression analysis, a **single food allergy** (P<0.001 SDL and total P=0.01EI, P=0.003 FA), and a **worse baseline FAQLQ-PF score** (P<0.001, all domains and total) predicted a greater improvement in FAQLQ-PF scores from start to maintenance/ cessation of treatment.  History of anaphylaxis was associated with greater improvement in single-factor analysis (<.05)  **Not significant**: asthma, food allergy treated, SHTD (starting dose), reactions during treatment, duration of OIT.  **Borderline significant** for EI and SDL: younger age  Among the 158 patients who reached full desensitization, younger age predicted a greater improvement in the FAQLQ-PF scores. (EI, P=0.029, FA, P=n.s., SDL, P=0.021, total score, P=0.042) | In a prospective cohort study, parents’ FAQLQ-PF scores significantly improved in all dimensions from OIT initiation to reaching full and/or partial maintenance, whereas there was no change in controls.  Worse baseline QOL, a single food allergy, and younger age predicted greater QOL improvements.  Among the subset of patients whose FAQLQ-PF total score deteriorated from baseline to mid up-dosing, FAQLQ-PF scores returned to near-baseline upon reaching maintenance.  Additional significant improvement in QOL was observed 6 months after reaching maintenance.  Limitations:  *Not randomized: there may have been significant differences between controls and OIT groups  *Only parent-reported QoL measures |
| **Factor, 2012 ^92^** – peanut  Case series | Build-up target: 450 mg PP /d  Build-up interval: 2 weeks | At baseline and on achieving the maintenance dose: Parents of 5 to 12-year-old children: **FAQLQ-PF**  8-12-year-old children: **FAQLQ-CF**  Teens (age 13-18): **FAQLQ-TF** | N=100  Age: 5-18 (85 aged 5-12, 35 aged 8-12, and 15 aged 13-18) | Significant improvement in QoL in all domains (AA, DR, RAE, EI, FA, and SDL) with the exception of the EI domain on the teens’ survey.  QoL significantly improved (P<.02) on all 30 questions when parents assessed their children 5 to 12 years old.  When children (8–12 years old) and teens assessed themselves, QoL improved (P<.05) on 22 of 24 questions and 12 of 18 questions, respectively.  Percentage of responses than decreased by ≤ 1 point by domain:   \| **Parents (n=76)** \| FA: 65.7%, SDL: 64.4%; EI: 55.1% \| \| --- \| --- \| \| **Children (n=32)** \| DR: 69.7%, RAE: 65.2%; AA: 62.7%; EI: 56.5% \| \| **Teens (n=14)** \| AADR: 71.9%, RAE: 67.9%; EI: 49.4% \|   See Figure bellow | NA | Limitations:  *No control group  *No analysis |

*n: number of patients with FAQLQ-PF data; N: number of patients enrolled; NA: not available; NR: not reported; T3: 3 months after build-up phase, T4: 12 months after build-up phase*

**FAIM**: Food Allergy Independent Measure: FAIM total score is based on 4 main questions, with a response scale from 0 (no chance) to 6 (very great chance): FAIM-1: chance of accidental exposure, FAIM-2: chance of a severe reaction on food exposure, FAIM-3: the chance of dying from food exposure and FAIM-4: chance of a child effectively treating him/herself or receiving effective treatment, following a food allergic reaction. Additional questions 5 and 6 ask (respectively) how many foods are avoided because of FA (categorized as single, 2, multiple >2) and how much FA limits the type of activities that the child can take part in. A reduced FAIM score indicates an improvement in the parent’s perception of the chance of an adverse outcome for their child with FA.

**FAQL-PB**: Food Allergy Quality of Life–Parental Burden: 7-point Likert scale (1 = not troubled, 7 = extremely troubled) for each question are summated, reporting the sum ranging from 17-119.

**FAQLQ-PF**: Food Allergy Quality of Life—Parent Form: for parents of children age 0-12 years: 7-point Scale (0- minimal impairment to 6 –maximal impairment), averaged over 3 areas with equal weighting: Emotional impact (EI), Food anxiety (FA), and Social and dietary limitations (SDL). The minimal clinically important difference (MCID) for FAQLQ-PF is 0.5.

**FAQLQ-CF**: Food Allergy Quality of Life—Child Form: for children age 8-12 years: 7-point Scale (1-not troubled to 7 –extremely troubled): 4 domains (24 items): Allergen avoidance (AA), Risk of accidental exposure (RAE), Emotional impact (EI), Dietary restrictions (DR). The minimal clinically important difference (MCID) for FAQLQ-CF is 0.5.

**MCID**: Minimally clinically important difference

**PedsQL 4.0**: Pediatric Quality of Life Inventory Version 4.0: 5-point Likert-scale (0=never, 4=almost always) for 8-18 year-olds, and a simplified 3-point scale for 5-7 year-olds. The 13 items are reverse-scored (0=100, 1=75, 2=50, 3=25, 4=0) reporting the mean sum of each item. The minimal clinically important difference (MCID) is ≥5.3 for child self-report and 5.5 for parental proxy report.

**Perceived treatment burden: VAS-form**: 3 domains: GI related AEs, taste and amount of daily peanut-dose, and time spent on OIT, range of VAS:0=no burden, 10=massive burden

## References

1. Anagnostou K, Islam S, King Y, et al. Assessing the efficacy of oral immunotherapy for the desensitisation of peanut allergy in children (STOP II): a phase 2 randomised controlled trial. *Lancet (London, England).* 2014;383(9925):1297-1304.

2. Blumchen K, Trendelenburg V, Ahrens F, et al. Efficacy, Safety, and Quality of Life in a Multicenter, Randomized, Placebo-Controlled Trial of Low-Dose Peanut Oral Immunotherapy in Children with Peanut Allergy. *The journal of allergy and clinical immunology In practice.* 2019;7(2):479-491.e410.

3. Reier-Nilsen T, Michelsen MM, Lodrup Carlsen KC, et al. Feasibility of desensitizing children highly allergic to peanut by high-dose oral immunotherapy. *Allergy.* 2019;74(2):337-348.

4. Tang ML, Ponsonby AL, Orsini F, et al. Administration of a probiotic with peanut oral immunotherapy: A randomized trial. *The Journal of allergy and clinical immunology.* 2015;135(3):737-744.e738.

5. Bird JA, Spergel JM, Jones SM, et al. Efficacy and Safety of AR101 in Oral Immunotherapy for Peanut Allergy: Results of ARC001, a Randomized, Double-Blind, Placebo-Controlled Phase 2 Clinical Trial. *The journal of allergy and clinical immunology In practice.* 2018;6(2):476-485.e473.

6. Vickery BP, Vereda A, Casale TB, et al. AR101 Oral Immunotherapy for Peanut Allergy. *The New England journal of medicine.* 2018;379(21):1991-2001.

7. Nachshon L, Goldberg MR, Katz Y, Levy MB, Elizur A. Long-term outcome of peanut oral immunotherapy-Real-life experience. *Pediatric allergy and immunology : official publication of the European Society of Pediatric Allergy and Immunology.* 2018;29(5):519-526.

8. Wasserman RL, Hague AR, Pence DM, et al. Real-World Experience with Peanut Oral Immunotherapy: Lessons Learned From 270 Patients. *The journal of allergy and clinical immunology In practice.* 2019;7(2):418-426.e414.

9. Soller L, Abrams EM, Carr S, et al. First Real-World Safety Analysis of Preschool Peanut Oral Immunotherapy. *The journal of allergy and clinical immunology In practice.* 2019.

10. Longo G, Barbi E, Berti I, et al. Specific oral tolerance induction in children with very severe cow's milk-induced reactions. *The Journal of allergy and clinical immunology.* 2008;121(2):343-347.

11. Martorell A, De la Hoz B, Ibanez MD, et al. Oral desensitization as a useful treatment in 2-year-old children with cow's milk allergy. *Clinical and experimental allergy : journal of the British Society for Allergy and Clinical Immunology.* 2011;41(9):1297-1304.

12. Morisset M, Moneret-Vautrin DA, Guenard L, et al. Oral desensitization in children with milk and egg allergies obtains recovery in a significant proportion of cases. A randomized study in 60 children with cow's milk allergy and 90 children with egg allergy. *European annals of allergy and clinical immunology.* 2007;39(1):12-19.

13. De Schryver S, Mazer B, Clarke AE, et al. Adverse Events in Oral Immunotherapy for the Desensitization of Cow's Milk Allergy in Children: A Randomized Controlled Trial. *The journal of allergy and clinical immunology In practice.* 2019.

14. Levy MB, Elizur A, Goldberg MR, Nachshon L, Katz Y. Clinical predictors for favorable outcomes in an oral immunotherapy program for IgE-mediated cow's milk allergy. *Annals of allergy, asthma & immunology : official publication of the American College of Allergy, Asthma, & Immunology.* 2014;112(1):58-63.e51.

15. Kauppila TK, Paassilta M, Kukkonen AK, Kuitunen M, Pelkonen AS, Makela MJ. Outcome of oral immunotherapy for persistent cow's milk allergy from 11 years of experience in Finland. *Pediatric allergy and immunology : official publication of the European Society of Pediatric Allergy and Immunology.* 2019.

16. Elizur A, Appel MY, Goldberg MR, et al. Clinical and laboratory 2-year outcome of oral immunotherapy in patients with cow's milk allergy. *Allergy.* 2016;71(2):275-278.

17. Burks AW, Jones SM, Plaut M. Oral Immunotherapy for Egg Allergy in Children REPLY. *New England Journal of Medicine.* 2012;367(15):1472-1473.

18. Escudero C, Rodriguez Del Rio P, Sanchez-Garcia S, et al. Early sustained unresponsiveness after short-course egg oral immunotherapy: a randomized controlled study in egg-allergic children. *Clinical and experimental allergy : journal of the British Society for Allergy and Clinical Immunology.* 2015;45(12):1833-1843.

19. Fuentes-Aparicio V, Alvarez-Perea A, Infante S, Zapatero L, D'Oleo A, Alonso-Lebrero E. Specific oral tolerance induction in paediatric patients with persistent egg allergy. *Allergologia et immunopathologia.* 2013;41(3):143-150.

20. Martin-Munoz MF, Belver MT, Alonso Lebrero E, et al. Egg oral immunotherapy in children (SEICAP I): Daily or weekly desensitization pattern. *Pediatric allergy and immunology : official publication of the European Society of Pediatric Allergy and Immunology.* 2019;30(1):81-92.

21. Martin-Munoz MF, Alonso Lebrero E, Zapatero L, et al. Egg OIT in clinical practice (SEICAP II): Maintenance patterns and desensitization state after normalizing the diet. *Pediatric allergy and immunology : official publication of the European Society of Pediatric Allergy and Immunology.* 2019;30(2):214-224.

22. Begin P, Winterroth LC, Dominguez T, et al. Safety and feasibility of oral immunotherapy to multiple allergens for food allergy. *Allergy Asthma Clin Immunol.* 2014;10(1):1.

23. Begin P, Dominguez T, Wilson SP, et al. Phase 1 results of safety and tolerability in a rush oral immunotherapy protocol to multiple foods using Omalizumab. *Allergy Asthma Clin Immunol.* 2014;10(1):7.

24. Nowak-Wegrzyn A, Wood RA, Nadeau KC, et al. Multicenter, randomized, double-blind, placebo-controlled clinical trial of vital wheat gluten oral immunotherapy. *The Journal of allergy and clinical immunology.* 2019;143(2):651-661.e659.

25. Kulmala P, Pelkonen AS, Kuitunen M, et al. Wheat oral immunotherapy was moderately successful but was associated with very frequent adverse events in children aged 6-18 years. *Acta paediatrica (Oslo, Norway : 1992).* 2018;107(5):861-870.

26. Elizur A, Appel MY, Nachshon L, et al. Walnut oral immunotherapy for desensitisation of walnut and additional tree nut allergies (Nut CRACKER): a single-centre, prospective cohort study. *The Lancet Child & adolescent health.* 2019;3(5):312-321.

27. Barni S, Mori F, Piccorossi A, et al. Low-Dose Oral Food Challenge with Hazelnut: Efficacy and Tolerability in Children. *International archives of allergy and immunology.* 2019;178(1):97-100.

28. Nachshon L, Goldberg MR, Levy MB, et al. Efficacy and Safety of Sesame Oral Immunotherapy-A Real-World, Single-Center Study. *The journal of allergy and clinical immunology In practice.* 2019.

29. Nurmatov U, Dhami S, Arasi S, et al. Allergen immunotherapy for IgE-mediated food allergy: a systematic review and meta-analysis. *Allergy.* 2017;72(8):1133-1147.

30. Romantsik O, Tosca MA, Zappettini S, Calevo MG. Oral and sublingual immunotherapy for egg allergy. *The Cochrane database of systematic reviews.* 2018;4:Cd010638.

31. Chu DK, Wood RA, French S, et al. Oral immunotherapy for peanut allergy (PACE): a systematic review and meta-analysis of efficacy and safety. *The lancet.* 2019.

32. Burks AW, Jones SM, Wood RA, et al. Oral immunotherapy for treatment of egg allergy in children. *The New England journal of medicine.* 2012;367(3):233-243.

33. Caminiti L, Passalacqua G, Barberi S, et al. A new protocol for specific oral tolerance induction in children with IgE-mediated cow's milk allergy. *Allergy and asthma proceedings.* 2009;30(4):443-448.

34. Caminiti L, Pajno GB, Crisafulli G, et al. Oral Immunotherapy for Egg Allergy: A Double-Blind Placebo-Controlled Study, with Postdesensitization Follow-Up. *The journal of allergy and clinical immunology In practice.* 2015;3(4):532-539.

35. Dello Iacono I, Tripodi S, Calvani M, Panetta V, Verga MC, Miceli Sopo S. Specific oral tolerance induction with raw hen's egg in children with very severe egg allergy: a randomized controlled trial. *Pediatric allergy and immunology : official publication of the European Society of Pediatric Allergy and Immunology.* 2013;24(1):66-74.

36. Lee JH, Kim WS, Kim H, Hahn YS. Increased cow's milk protein-specific IgG4 levels after oral desensitization in 7- to 12-month-old infants. *Annals of allergy, asthma & immunology : official publication of the American College of Allergy, Asthma, & Immunology.* 2013;111(6):523-528.

37. Meglio P, Giampietro PG, Carello R, Gabriele I, Avitabile S, Galli E. Oral food desensitization in children with IgE-mediated hen's egg allergy: a new protocol with raw hen's egg. *Pediatric allergy and immunology : official publication of the European Society of Pediatric Allergy and Immunology.* 2013;24(1):75-83.

38. Pajno GB, Caminiti L, Ruggeri P, et al. Oral immunotherapy for cow's milk allergy with a weekly up-dosing regimen: a randomized single-blind controlled study. *Annals of allergy, asthma & immunology : official publication of the American College of Allergy, Asthma, & Immunology.* 2010;105(5):376-381.

39. Patriarca G, Schiavino D, Nucera E, Schinco G, Milani A, Gasbarrini GB. Food allergy in children: results of a standardized protocol for oral desensitization. *Hepato-gastroenterology.* 1998;45(19):52-58.

40. Skripak JM, Nash SD, Rowley H, et al. A randomized, double-blind, placebo-controlled study of milk oral immunotherapy for cow's milk allergy. *The Journal of allergy and clinical immunology.* 2008;122(6):1154-1160.

41. Staden U, Rolinck-Werninghaus C, Brewe F, Wahn U, Niggemann B, Beyer K. Specific oral tolerance induction in food allergy in children: efficacy and clinical patterns of reaction. *Allergy.* 2007;62(11):1261-1269.

42. Varshney P, Jones SM, Scurlock AM, et al. A randomized controlled study of peanut oral immunotherapy: clinical desensitization and modulation of the allergic response. *The Journal of allergy and clinical immunology.* 2011;127(3):654-660.

43. Garcia-Ara C, Pedrosa M, Belver MT, Martin-Munoz MF, Quirce S, Boyano-Martinez T. Efficacy and safety of oral desensitization in children with cow's milk allergy according to their serum specific IgE level. *Annals of allergy, asthma & immunology : official publication of the American College of Allergy, Asthma, & Immunology.* 2013;110(4):290-294.

44. Martinez-Botas J, Rodriguez-Alvarez M, Cerecedo I, et al. Identification of novel peptide biomarkers to predict safety and efficacy of cow's milk oral immunotherapy by peptide microarray. *Clinical and experimental allergy : journal of the British Society for Allergy and Clinical Immunology.* 2015;45(6):1071-1084.

45. Syed A, Garcia MA, Lyu SC, et al. Peanut oral immunotherapy results in increased antigen-induced regulatory T-cell function and hypomethylation of forkhead box protein 3 (FOXP3). *The Journal of allergy and clinical immunology.* 2014;133(2):500-510.

46. Mansouri M, Movahhedi M, Pourpak Z, Akramian R, Shokohi Shormasti R, Mozaffari H. Oral desensitization in children with IgE-mediated cow’s milk allergy: a prospective clinical trial. *Tehr Univ Med J.* 2007;65:11-18.

47. Patriarca G, Nucera E, Roncallo C, et al. Oral desensitizing treatment in food allergy: clinical and immunological results. *Alimentary pharmacology & therapeutics.* 2003;17(3):459-465.

48. Patriarca G, Nucera E, Pollastrini E, et al. Oral specific desensitization in food-allergic children. *Digestive diseases and sciences.* 2007;52(7):1662-1672.

49. Fleischer DM, Burks AW, Vickery BP, et al. Sublingual immunotherapy for peanut allergy: a randomized, double-blind, placebo-controlled multicenter trial. *The Journal of allergy and clinical immunology.* 2013;131(1):119-127 e111-117.

50. Kim EH, Bird JA, Kulis M, et al. Sublingual immunotherapy for peanut allergy: clinical and immunologic evidence of desensitization. *The Journal of allergy and clinical immunology.* 2011;127(3):640-646 e641.

51. Akashi M, Yasudo H, Narita M, et al. Randomized controlled trial of oral immunotherapy for egg allergy in Japanese patients. *Pediatrics international : official journal of the Japan Pediatric Society.* 2017;59(5):534-539.

52. Giavi S, Vissers YM, Muraro A, et al. Oral immunotherapy with low allergenic hydrolysed egg in egg allergic children. *Allergy.* 2016;71(11):1575-1584.

53. Perez-Rangel I, Rodriguez Del Rio P, Escudero C, Sanchez-Garcia S, Sanchez-Hernandez JJ, Ibanez MD. Efficacy and safety of high-dose rush oral immunotherapy in persistent egg allergic children: A randomized clinical trial. *Annals of allergy, asthma & immunology : official publication of the American College of Allergy, Asthma, & Immunology.* 2017;118(3):356-364.e353.

54. Vazquez-Ortiz M, Alvaro M, Piquer M, et al. Baseline specific IgE levels are useful to predict safety of oral immunotherapy in egg-allergic children. *Clinical and experimental allergy : journal of the British Society for Allergy and Clinical Immunology.* 2014;44(1):130-141.

55. Fauquert JL, Michaud E, Pereira B, et al. Peanut gastrointestinal delivery oral immunotherapy in adolescents: Results of the build-up phase of a randomized, double-blind, placebo-controlled trial (PITA study). *Clinical and experimental allergy : journal of the British Society for Allergy and Clinical Immunology.* 2018;48(7):862-874.

56. Wasserman RL, Factor JM, Baker JW, et al. Oral immunotherapy for peanut allergy: multipractice experience with epinephrine-treated reactions. *The journal of allergy and clinical immunology In practice.* 2014;2(1):91-96.

57. The Cochrane Collaboration. Cochrane Handbook for Systematic Reviews of Interventions. In: Higgins J, Green S, eds.2011: <http://handbook-5-1.cochrane.org/>.

58. Institute of Health Economics (IHE). Quality Appraisal of Case Series Studies Checklist. In:2014.

59. Pajno GB, Fernandez-Rivas M, Arasi S, et al. EAACI Guidelines on allergen immunotherapy: IgE-mediated food allergy. *Allergy.* 2018;73(4):799-815.

60. Jones SM, Burks AW, Keet C, et al. Long-term treatment with egg oral immunotherapy enhances sustained unresponsiveness that persists after cessation of therapy. *The Journal of allergy and clinical immunology.* 2016;137(4):1117-1127.e1110.

61. Hsiao KC, Ponsonby AL, Axelrad C, Pitkin S, Tang MLK. Long-term clinical and immunological effects of probiotic and peanut oral immunotherapy after treatment cessation: 4-year follow-up of a randomised, double-blind, placebo-controlled trial. *The Lancet Child & adolescent health.* 2017;1(2):97-105.

62. Meglio P, Giampietro PG, Carello R, Galli E. Oral immunotherapy in children with IgE-mediated hen's egg allergy: Follow-ups at 2.5 and 7 years. *Allergy & rhinology (Providence, RI).* 2017;8(3):157-169.

63. Sampson HA, Leung DYM, Jones SM, et al. Egg Oral Immunotherapy (OIT) Induces More Rapid Desensitization and Sustained Unresponsiveness (SU) in Egg-Allergic, Baked-egg Tolerant Children than the Addition of Daily Baked-egg Products. *Journal of Allergy and Clinical Immunology.* 2019;143(2):AB255-AB255.

64. Vickery BP, Scurlock AM, Kulis M, et al. Sustained unresponsiveness to peanut in subjects who have completed peanut oral immunotherapy. *The Journal of allergy and clinical immunology.* 2014;133(2):468-475.

65. Nagakura KI, Yanagida N, Sato S, et al. Low-dose oral immunotherapy for children with anaphylactic peanut allergy in Japan. *Pediatric allergy and immunology : official publication of the European Society of Pediatric Allergy and Immunology.* 2018;29(5):512-518.

66. Narisety SD, Frischmeyer-Guerrerio PA, Keet CA, et al. A randomized, double-blind, placebo-controlled pilot study of sublingual versus oral immunotherapy for the treatment of peanut allergy. *The Journal of allergy and clinical immunology.* 2015;135(5):1275-1282.e1271-1276.

67. Takahashi M, Taniuchi S, Soejima K, Hatano Y, Yamanouchi S, Kaneko K. Two-weeks-sustained unresponsiveness by oral immunotherapy using microwave heated cow's milk for children with cow's milk allergy. *Allergy, asthma, and clinical immunology : official journal of the Canadian Society of Allergy and Clinical Immunology.* 2016;12(1):44.

68. Vickery BP, Berglund JP, Burk CM, et al. Early oral immunotherapy in peanut-allergic preschool children is safe and highly effective. *The Journal of allergy and clinical immunology.* 2017;139(1):173-181.e178.

69. Yanagida N, Sato S, Asaumi T, Nagakura K, Ogura K, Ebisawa M. Safety and Efficacy of Low-Dose Oral Immunotherapy for Hen's Egg Allergy in Children. *International archives of allergy and immunology.* 2016;171(3-4):265-268.

70. Burks A, Casale T, Beyer K, et al. Age-Related Findings from the Peanut Allergy Oral Immunotherapy Study of Ar101 for Desensitization (Palisade) Study. *Annals of allergy, asthma and immunology.* 2018;Conference:. 2018 annual scientific meeting of the american college of allergy asthma and immunology. United states 121(5 Supplement):S4.

71. Mantyla J, Thomander T, Hakulinen A, et al. The effect of oral immunotherapy treatment in severe IgE mediated milk, peanut, and egg allergy in adults. *Immunity, inflammation and disease.* 2018;6(2):307-311.

72. Blumchen K, Beder A, Beschorner J, et al. Modified oral food challenge used with sensitization biomarkers provides more real-life clinical thresholds for peanut allergy. *The Journal of allergy and clinical immunology.* 2014;134(2):390-398.

73. Elizur A, Goldberg MR, Levy MB, Nachshon L, Katz Y. Oral immunotherapy in cow's milk allergic patients: course and long-term outcome according to asthma status. *Annals of allergy, asthma & immunology : official publication of the American College of Allergy, Asthma, & Immunology.* 2015;114(3):240-244.e241.

74. Cox L, Larenas-Linnemann D, Lockey RF, Passalacqua G. Speaking the same language: The World Allergy Organization Subcutaneous Immunotherapy Systemic Reaction Grading System. *The Journal of allergy and clinical immunology.* 2010;125(3):569-574, 574 e561-574 e567.

75. Sampson HA. Anaphylaxis and emergency treatment. *Pediatrics.* 2003;111(6 Pt 3):1601-1608.

76. Muraro A, Roberts G, Clark A, et al. The management of anaphylaxis in childhood: position paper of the European academy of allergology and clinical immunology. *Allergy.* 2007;62(8):857-871.

77. Manabe T, Sato S, Yanagida N, et al. Long-term outcomes after sustained unresponsiveness in patients who underwent oral immunotherapy for egg, cow's milk, or wheat allergy. *Allergology international : official journal of the Japanese Society of Allergology.* 2019.

78. Lucendo AJ, Arias A, Tenias JM. Relation between eosinophilic esophagitis and oral immunotherapy for food allergy: a systematic review with meta-analysis. *Ann Allergy Asthma Immunol.* 2014;113(6):624-629.

79. Goldberg MR, Elizur A, Nachshon L, et al. Oral immunotherapy-induced gastrointestinal symptoms and peripheral blood eosinophil responses. *The Journal of allergy and clinical immunology.* 2017;139(4):1388-1390.e1384.

80. Goldberg MR, Nachshon L, Levy MB, Elizur A, Katz Y. Risk Factors and Treatment Outcomes for Oral Immunotherapy-Induced Gastrointestinal Symptoms and Eosinophilic Responses (OITIGER). *The journal of allergy and clinical immunology In practice.* 2019.

81. Echeverria-Zudaire LA, Fernandez-Fernandez S, Rayo-Fernandez A, Munoz-Archidona C, Checa-Rodriguez R. Primary eosinophilic gastrointestinal disorders in children who have received food oral immunotherapy. *Allergol Immunopathol (Madr).* 2016;44(6):531-536.

82. Gomez Torrijos E, Mendez Diaz Y, Moreno Lozano L, et al. Frequency and Course of Eosinophilic Esophagitis During Oral Immunotherapy for Cow's Milk Allergy in a Series of 57 Children. *Journal of investigational allergology & clinical immunology.* 2017;27(2):132-133.

83. Rodriguez CG, Torrijos EG, De la Pinzon FR, et al. Dysphagia in a boy treated with oral immunotherapy for cow's milk allergy. *Journal of investigational allergology & clinical immunology.* 2014;24(5):363-365.

84. Vazquez-Ortiz M, Alvaro-Lozano M, Alsina L, et al. Safety and predictors of adverse events during oral immunotherapy for milk allergy: severity of reaction at oral challenge, specific IgE and prick test. *Clinical and experimental allergy : journal of the British Society for Allergy and Clinical Immunology.* 2013;43(1):92-102.

85. Virkud YV, Burks AW, Steele PH, et al. Novel baseline predictors of adverse events during oral immunotherapy in children with peanut allergy. *The Journal of allergy and clinical immunology.* 2017;139(3):882-888.e885.

86. Barbi E, Longo G, Berti I, et al. Adverse effects during specific oral tolerance induction: in home phase. *Allergologia et immunopathologia.* 2012;40(1):41-50.

87. Barbi E, Longo G, Berti I, et al. Adverse effects during specific oral tolerance induction: in-hospital "rush" phase. *European annals of allergy and clinical immunology.* 2012;44(1):18-25.

88. Vazquez-Ortiz M, Alvaro M, Piquer M, et al. Life-threatening anaphylaxis to egg and milk oral immunotherapy in asthmatic teenagers. *Annals of allergy, asthma & immunology : official publication of the American College of Allergy, Asthma, & Immunology.* 2014;113(4):482-484.

89. Dunn Galvin A, McMahon S, Ponsonby AL, Hsiao KC, Tang MLK. The longitudinal impact of probiotic and peanut oral immunotherapy on health-related quality of life. *Allergy.* 2018;73(3):560-568.

90. Reier-Nilsen T, Carlsen KCL, Michelsen MM, et al. Parent and child perception of quality of life in a randomized controlled peanut oral immunotherapy trial. *Pediatric allergy and immunology : official publication of the European Society of Pediatric Allergy and Immunology.* 2019.

91. Epstein-Rigbi N, Goldberg MR, Levy MB, Nachshon L, Elizur A. Quality of Life of Food-Allergic Patients Before, During, and After Oral Immunotherapy. *The journal of allergy and clinical immunology In practice.* 2019;7(2):429-436.e422.

92. Factor JM, Mendelson L, Lee J, Nouman G, Lester MR. Effect of oral immunotherapy to peanut on food-specific quality of life. *Annals of allergy, asthma & immunology : official publication of the American College of Allergy, Asthma, & Immunology.* 2012;109(5):348-352.e342.

93. Remington BC, Krone T, Koppelman SJ. Quantitative risk reduction through peanut immunotherapy: Safety benefits of an increased threshold in Europe. *Pediatric allergy and immunology : official publication of the European Society of Pediatric Allergy and Immunology.* 2018;29(7):762-772.

94. Baumert JL, Taylor SL, Koppelman SJ. Quantitative Assessment of the Safety Benefits Associated with Increasing Clinical Peanut Thresholds Through Immunotherapy. *The journal of allergy and clinical immunology In practice.* 2018;6(2):457-465.e454.

95. Aimmune Therapeutics. *Allergenic Products Advisory Committee Meeting (APAC) Briefing Document - Peanut, Arachis hypogaea, Allergen Powder for Oral Administration (Proposed Trade Name: Palforzia).* 2019.

96. Eapen AA, Lavery WJ, Siddiqui JS, et al. Oral Immunotherapy for Multiple Foods in a Pediatric Allergy Clinic Setting. *Annals of allergy, asthma & immunology : official publication of the American College of Allergy, Asthma, & Immunology.* 2019.

97. Andorf S, Purington N, Block WM, et al. Anti-IgE treatment with oral immunotherapy in multifood allergic participants: a double-blind, randomised, controlled trial. *Lancet Gastroenterol Hepatol.* 2018;3(2):85-94.

98. Martorell A, Alonso E, Echeverria L, et al. Oral Immunotherapy for Food Allergy: A Spanish Guideline. Immunotherapy Egg and Milk Spanish Guide (ITEMS Guide). Part I: Cow Milk and Egg Oral Immunotherapy: Introduction, Methodology, Rationale, Current State, Indications, Contraindications, and Oral Immunotherapy Build-up Phase. *J Investig Allergol Clin Immunol.* 2017;27(4):225-237.

99. Wasserman RL, Jones DH, Windom HH. Oral immunotherapy for food allergy: The FAST perspective. *Annals of allergy, asthma & immunology : official publication of the American College of Allergy, Asthma, & Immunology.* 2018;121(3):272-275.

100. Pajno GB, Caminiti L, Salzano G, et al. Comparison between two maintenance feeding regimens after successful cow's milk oral desensitization. *Pediatric allergy and immunology : official publication of the European Society of Pediatric Allergy and Immunology.* 2013;24(4):376-381.

101. Hill DA, Dudley JW, Spergel JM. The Prevalence of Eosinophilic Esophagitis in Pediatric Patients with IgE-Mediated Food Allergy. *The journal of allergy and clinical immunology In practice.* 2017;5(2):369-375.

102. Wood RA, Kim JS, Lindblad R, et al. A randomized, double-blind, placebo-controlled study of omalizumab combined with oral immunotherapy for the treatment of cow's milk allergy. *The Journal of allergy and clinical immunology.* 2016;137(4):1103-1110.e1111.

103. MacGinnitie AJ, Rachid R, Gragg H, et al. Omalizumab facilitates rapid oral desensitization for peanut allergy. *J Allergy Clin Immunol.* 2017;139(3):873-881 e878.

104. Takahashi M, Taniuchi S, Soejima K, Sudo K, Hatano Y, Kaneko K. New efficacy of LTRAs (montelukast sodium): it possibly prevents food-induced abdominal symptoms during oral immunotherapy. *Allergy Asthma Clin Immunol.* 2014;10(1):3.

105. Yokooji T, Matsuo H. Sodium Cromoglycate Prevents Exacerbation of IgE-Mediated Food-Allergic Reaction Induced by Aspirin in a Rat Model of Egg Allergy. *International archives of allergy and immunology.* 2015;167(3):193-202.

106. Hirano I, Dellon ES, Hamilton JD, et al. Efficacy of Dupilumab in a Phase 2 Randomized Trial of Adults With Active Eosinophilic Esophagitis. *Gastroenterology.* 2020;158(1):111-122 e110.

# Appendix 4: Narrative summaries of the data that led to the recommendations

## Recommendations on patient-centered care

Four studies in which parents completed the FAQLQ-PF questionnaire (a validated HRQoL instrument in food allergy) regarding the HRQoL of their allergic child both at baseline and after the build-up phase reported statistically and clinically significant improvements in the Total score,^1,89,91,92^ with the greatest improvements in the Food Anxiety domain.^89,91^ Two of them had randomized^89^ or non-randomized^91^ control groups, in which HRQoL did not change significantly.^89,91^ The RCT observed that 77% of OIT patients reported clinically significant improvement in the Total score three months after completion of double-blind treatment compared to 34% of patients that had been randomized to placebo (P=significant),^89^ with achieving sustained unresponsiveness significantly associated with larger QoL improvements.^89^ FAQLQ-PF data for 66 patients from one Canadian OIT clinic also showed significant improvements upon completion of OIT (data on file). In contrast, in one double-blind peanut OIT RCT there were no statistically significant changes in FAQLQ-PF scores in the OIT arm, although there where numerical improvements.^2^

Two of the FAQLQ studies included self-reported data from a small number of children and teenagers (N=17 and 46);^2,92^ these reported significant improvements from baseline in the Total score and in most of its sub-domains.

One open-label RCT^90^ used the generic Pediatric Quality of Life Inventory Version 4.0 questionnaire. The mean total score improved significantly from baseline to year 2 in the OIT group in both the children and their parents, with no significant changes in the control group; differences in the changes were statistically significant for the parents only.

In a longitudinal study following 175 children, the mean FAQLQ-PF scores improved from baseline through mid-up-dosing to reaching maintenance and further at 6 months into maintenance, with the greatest improvements seen during maintenance.^91^ In this cohort, a subset of patients reported deterioration in QoL during mid-up-dosing with a return to baseline levels upon reaching maintenance.^91^ Having a history of anaphylactic reactions and worse food-allergy related QoL at baseline was associated with larger QoL improvements.^91^ The type of food allergy treated (peanut, egg, milk) was not significant, but having multiple food allergies (in the context of single-food OIT) was associated with less positive QoL outcomes.^91^

## Recommendations on eligible food allergens and clinical outcomes that can be achieved by OIT

A majority of patients undergoing OIT tolerate a higher amount of all food allergens at the end of the build-up phase (*at least* *partial desensitization*: peanut: 73-90% [^31^see note under Figure 1],^9,56^ chicken’s egg: 82%,^30^ cow’s milk: 78-89%,^14,15,29^ hazelnut: 65%,^27^ sesame: 100%^28^), a significantly higher proportion compared to control patients in all controlled studies (Figure 1). (Note: For peanuts, an increase in the eliciting dose from less than 100 mg to 300 mg peanut protein was estimated to provide an at least 95% reduction of the risk of an allergic reaction stemming from exposure to traces of peanut in packaged foods.^93,94^) Many patients can tolerate a full serving (*complete desensitization*: chicken’s egg: 45-84%,^20,30^ cow’s milk: 60-71%,^13-15^ wheat: 52-64%,^24,25^ walnut: 89%,^26^ sesame: 88%^28^) or a cumulative dose of at least 1 g peanut protein (4.2 peanuts) (56-78%) (Figure 1).^8,31^ A sizable proportion of patients continue consuming the food allergen regularly in the longer term (*continued consumption*: peanut: 35-78%,^7,61^ chicken’s egg: 58-70%,^60,62^ wheat: 39%,^25^ walnut: 70%^26^, hazelnut: 65%,^27^ sesame: 88%^28^) and a variable proportion can maintain tolerance to the allergen after a period of food avoidance (*sustained unresponsiveness*: peanut: 13-74%^4,8^ chicken’s egg: 35-44%^29,63^ cow’s milk: 21%,^67^ wheat: 13%^67^), but the amount of available data for this outcome is limited.

Patients undergoing OIT are more likely to experience allergic reactions related to consuming the OIT food allergen dose than patients who are avoiding the food. A majority of OIT patients have at least one allergic reaction, and, based on meta-analyses of milk, egg and peanut OIT trials, 16% to 17% of patients experience systemic or anaphylactic reactions (vs 1.6-2.6% of control patients),^29,31^ which require the use of epinephrine in 8.4% to 12% of patients (vs 0-3.7% of control patients).^30,31^ One meta-analysis estimated that peanut OIT approximately doubled the risk of serious adverse events (SAEs) (6.2% vs 3.0% control).^31^ Quality assessment of this analysis based on original source publications indicated misclassification of adverse events in some cases, suggesting that the rate of SAEs may be similar between OIT and control groups.

Analyses of data from double-blind RCTs of peanut OIT indicate that adverse events related to accidental exposure to the food allergen occur in fewer patients undergoing OIT than in patients receiving placebo.^2,95^

The average rate of discontinuation due to adverse events in peanut OIT RCTs has been estimated at 13% compared to 3.7% of control patients.^31^ Discontinuation rates due to adverse events ranged from 0 to 18% across egg^12,17-19,21^ and wheat^24^ RCTs, sesame^28^ and walnut^26^ CCTs as well as in peanut^8^ and milk^14,15^ clinical practice. One exception is a wheat case series, in which an overall of 43% of patients discontinued OIT, 40% of them due to mild or moderate adverse events occurring during the 12-month maintenance.^25^

Many studies across different designs and food allergens report that the occurrence or severity of allergic reactions declines as the treatment progresses from the build-up to the maintenance phase.^2,3,8,9,13,20,25,26,28,32,56^

## Recommendations on who could benefit from OIT (indications)

Most food OIT studies included children and adolescents across wide age ranges. Large case series of milk and peanut OIT, including patients starting from the age of four and up to 27 years, reported no significant association between age and safety outcomes and inconsistent observations regarding efficacy.^7,14-16^

Three studies focused on toddlers and pre-school children (age 1-5 years) (2 peanut^9,68^ and 1 milk OIT^11^); they indicate high efficacy (81-90% desensitization,^9,11,68^ 78% sustained unresponsiveness,^68^ 90% continued consumption^11^) and an excellent safety profile in this age group (0-0.4% had severe reactions). One peanut OIT RCT focused on adolescents (age 12-17 years); it reported a desensitization rate (400 mg peanut protein) of 81% compared to 11% for the placebo group. A second RCT, performing a sub-group analysis, reported that absolute differences in desensitization rates between OIT and placebo groups were comparable for adolescents and children aged 4 to 11 years (58% [95% CI 40 to 77%] and 66% [95% CI 54% to 78%] respectively).^70,6^ The same study also included adults (for a secondary end-point analysis), reporting a desensitization rate of 41% with OIT vs 14.3% with placebo, but the number of patients was small (41 in OIT and 14 in placebo group) and the difference was not statistically significant.^6,70^ One small case series of 23 adults receiving OIT reported significant increases in the amount of protein that patients were able to tolerate.^71^

## Recommendations on contra-indications

A history of anaphylactic reactions to the targeted food allergen was generally not an exclusion criterion in OIT studies. Evidence from large case series on whether baseline history of anaphylaxis had an impact on OIT outcomes is inconsistent,^7,8,14,16^ but most patients with a history of anaphylaxis were able to achieve at least partial desensitization.^8,14^

Across two reports of OIT clinical practice, there was no correlation between a patient’s number of food allergies and the outcomes of single-food OIT.^14^ ^7^ Studies of multi-food OIT targeting patients with multiple food allergies raise no particular safety or efficacy issues in this patient demographic.^22,23,96,97^

In many RCTs^2-6,10,13,20^ and reports of clinical practice,^7,9,14^ severe and/or poorly controlled or unstable asthma was an exclusion criterion for OIT. Baseline asthma was associated with an increased risk of adverse reactions in large case series, ^8,73,85,87^ and asthma exacerbation was recorded as an adverse event in peanut and egg RCTs.^6,20^ Nevertheless, most patients with controlled asthma were able to achieve at least partial desensitization.^7,8,15,73^

OIT requires patients (and/or their caregivers) to regularly attend visits, understand and follow instructions regarding administering the treatment at home, and be able to recognize and treat adverse events.^59,98,99^

## Recommendations on personalized protocols

Published OIT protocols vary in terms of food allergen product and preparation, initial dose escalation, build-up starting and target dose, up-doing frequency, length of build-up phase and maintenance dose and frequency (see Table A1 in Appendix 2).

Most OIT clinical trials and all OIT clinical practice studies used non-pharmaceutical food-based products. There are no head-to-head comparisons between pharmaceutical and food-based products. Meta-analysis of peanut OIT RCTs found that both proprietary and non-proprietary OIT products led to desensitization versus placebo or usual care (non-proprietary: 67% [64/93] vs 7.5% ([4/53]; proprietary: 53% [256/481] vs 2.2% [5/231]).^31^

In terms of data directly comparing different OIT protocols, one egg OIT RCT indicated better efficacy outcomes when weekly up-dosing was combined with small daily dose increments than with weekly up-dosing only.^29^ With respect to safety, this approach was associated with higher rates of mild and local reactions but lower rates of moderate to severe reactions.^29^ Two small RCTs reported similar efficacy outcomes comparing different target or maintenance doses;^24,68^ however, in a clinical practice case series patients appeared to be more likely to continue peanut consumption when the maintenance dose was lowered.^7^ Consuming the maintenance dose daily versus every second day was associated with fewer reactions and better adherence in an egg OIT RCT,^21^ but in a milk OIT RCT there was no difference in the frequency of adverse events between daily and weekly consumption after one year of maintenance.^100^

With respect to treating multiple food allergies, one non-randomized study (N=40) observed similar rates of reaction per dose with multiple-food OIT (targeting up to 5 foods simultaneously) as compared to single-food OIT, while time to reach 10-fold increase in desensitization threshold was only 3.2 months longer with multi-food OIT as compared to single-food OIT.^22^

## Recommendations for the safe provision of OIT

Clinical studies of OIT report that anaphylactic reactions or reactions requiring the use of epinephrine may occur in the clinic as well as during home dosing. Some studies reporting fewer reactions at home than in the clinic,^9,14,26,28^ while others report more reactions at home.^8,13,56^ Note that up-dosing, which is associated with a higher risk of reaction, occurs in the clinic, but the proportion of doses administered at home is usually higher and increases with the duration of treatment. These factors could contribute to the observed differences between studies.

EoE is more prevalent in children with food allergy compared to the general pediatric population (4.7% vs 0.04%) and is particularly associated with milk and egg allergy.^101^ Biopsy-confirmed EoE occurred in 0.4% (peanut) to 6.3% (milk) of patients undergoing OIT.^2,5,6,55,66,78,81-83^ Recurrent gastro-intestinal symptoms indicative of EoE were reported in 8.2 to 14% of OIT-treated children^8,79^ and 1.1%^9^ of pre-school children and were managed with dose adjustments.^80^

Three RCTs compared OIT in the presence of omalizumab versus OIT with placebo.^102^ Omalizumab did not improve efficacy outcomes at 28 or 32 months when used for an extended time with a standard slow OIT schedule;^102^ however, a short course of omalizumab within an accelerated OIT schedule significantly increased desensitization rates.^97,103^ Omalizumab use was also associated with a reduced rate of dosing reactions.^97,102^

# Appendix 5: Equipment required

The following tables on the minimal and additional safety requirements for the management of emergencies in allergy office are from the CSACI position paper on safety standards for the management of office emergencies (in redaction).

Table S5.1: Minimal Safety Medications, Equipment and Supplies

| Category |  |
| --- | --- |
| Vital sign assessment | - Stethoscope  - Sphygmomanometer and blood pressure cuffs - Oxygen saturation monitor  - Personal protective equipment (gloves, mask, eye shield)  - Watch or clock |
| Medications | - Intramuscular epinephrine (3 doses)  - Glucagon or vasopressin for adults on beta blocker  - Salbutamol (with MDI and spacer or nebulizer)  - Second generation antihistamine |
| Airway | - Oropharyngeal airway (adult and pediatric) |
| Breathing | - Self inflating bag-valve-mask (adult and pediatric)  - Disposable face masks (adult and pediatric)  - Oxygen tank  - Oxygen extension tubing  - Oxygen nasal cannula  - Non rebreather mask (adult and pediatric) |
| Circulation | - Tourniquet  - Tape  - Alcohol swabs  - Drip chamber  - Syringe with needles  - T-connector  - Extension tubing  - Intravenous 0.9 normal saline (two 1 liter bags)  A method to establish parenteral access which could include any of:  - Intravenous butterfly needles  - Indwelling catheters  -Intraosseous devices |
| Other | - Written anaphylaxis management protocol  - Flow chart for recording times and events  -911 script for office staff to use |

Table S5.2: Additional equipment and medications to consider, depending on provider experience, skill and location

| Category |  |
| --- | --- |
| Vital sign assessment | - Automated BP cuff, HR, and O2 sat monitor  - 5 minute timer |
| Airway | - Portable suction  - Nasal airway  - Laryngeal airway masks (LMA) with lubrication  - Laryngoscope with blades, ET tubes, stylet and CO2 detector, tape and suction, and Magill forceps  - Alternative airway devices (eg: King Airway) |
| Breathing | - Nebulizer mask |
| Circulation | - Pediatric Armboard  - Set up for 3 way stopcock for pediatric fluid bolus  - Intraosseous devices  - AED |
| Medications for treatment of allergic conditions | - Ipatropium bromide (spacer with MDI or nebulizer)  - Diphenhydramine IV  - Corticosteroid for injection |
| Medications for treatment of non allergic conditions | - Nitroglycerine spray  - ASA  - Naloxone  - Lorazepam or diazepam  - Glucose gel |

# Appendix 6: Sample protocols

The following sample protocols are based on the protein content for the allergen(s) included in the OIT treatment. They actual amount of food given will therefore depend on the protein content of the allergen.

The following equivalency table provides protein content for foods frequently used for OIT, based on the USDA food database. Because variations in protein content are to be expected according to brand or nut size, patients should be advised to use the same product at home as the one used in clinic during the up-dosing phase. Ideally, new products are introduced during up-dosing visits. Once maintenance has been achieved and is well tolerated, patients can usually experiments with new food alternatives safely, provided they understand the concept of food equivalency. Consultation with registered dieticians experienced in OIT and teaching tools to that effect can be very helpful to assist patients with equivalent food alternatives.

For food containing heat-labile allergens, such as milk or egg, special consideration should be given to the product used in OIT. Where desensitization with raw forms will protect against cooked forms, the reverse may not be true. In practice, patient objectives regarding heat-labile and heat-resistant allergens are often not the same. Where patients often wish to achieve complete desensitization to cooked forms to allow their introduction in diet and reduce limitations, most only wish to achieve partial desensitization against raw forms to protect against accidental contamination. The choice of products should reflect those objectives, which can change during therapy as patients discover their love or dislike of the food they had been avoiding. For example, a target daily maintenance dose for milk allergy could be 15mL of fresh milk (0.5g protein) and 1 whole portion of string cheese (6g protein).

**Measurement of daily doses**

**Whole units**: With larger dose amounts, easily measurable of food units can be used (e.g. ¼ peanut snack stick, 1 cm of string cheese).

**Weight:** With smaller amounts, individual doses can be measured precisely with scale in soufflé cups or capsules. Usual practice aims for a level of precision of ± 10%. If this task is delegated, proper training is essential to avoid dosing errors and cross-contamination and ensure traceability.

**Volume:** A practical alternative to weighing individual doses consists in using syringes or measuring spoons for patient to calculate daily dose for liquid food or suspension.

The suspension media must be adapted to the food. For example, defatted peanut or sesame flour are best suspended in simple syrup suspension. Nut meals, on the other hands tend to float in simple syrup because of their oil content. Suspension vehicles with suspending and anti-foam agents (e.g. oral-blend or oral mix) are therefore better suited for nut meals.

*Example of Recipe for 60 mL of peanut suspension at 250mg protein per 5mL (50mg/mL):*

In a sterile specimen cup, add 6g of defatted peanut flour (or 7.2g of powdered peanut butter).

Add simple syrup up to the 50mL line. Mix thoroughly with spoon. Complete with simple syrup up to the 60 mL line. Shake. Keep refrigerated.

On the label, make sure to indicate:

- Patient name
- Content and concentration (e.g. peanut 250mg/5mL)
- Date of production
- Peanut flour lot
- Peanut flour expiry date

## Equivalency table: protein content per type of allergen

Protein content is based on the USDA database. Exact allergen content may vary according to brand. Food escalations in clinic should therefore be performed with the same product that will be used at home.

| **Allergen** | | **Amount of protein** |
| --- | --- | --- |
| **Chicken’s egg** | 1 mg of powdered-egg whites | 1.25 mg |
|  | 1 whole egg | 5000 mg |
|  | 1 ml raw egg* mixed | 100 mg |
|  | **Note:** Raw egg is not recommended under 1 year old unless pasteurized due to risk of bacterial contamination | |
| **Peanut** | 1 peanut | 250 mg |
|  | 1 ml Peanut butter ( Kraft®) | 200 mg |
|  | 2.4 mg of powdered peanut butter (PB2®) | 1 mg |
|  | 2 mg of defatted peanut flour | 1 mg |
|  | 0.1 mL of peanut flour suspension (50mg/mL) | 5 mg |
|  | 1 peanut stick snack (Bamba®) | 80 mg |
| **Cow’s milk** | 1 ml milk (any percent of fat) | 35 mg |
|  | 2.8 mg of powdered milk | 1 mg |
|  | 1 cm of string cheese stick  (6g protein for 12cm stick) | 500 mg |
| **Mustard** | 1 ml yellow mustard | 40 mg |
| **Hazelnut** | 1 table spoon of Nutella ® | 300 mg |
|  | 1 hazelnut | 180 mg |
|  | Hazelnut meal | 1 mg |
| **Almond** | 1 almond | 275 mg |
|  | 4.6 mg of almond meal/flour, non-defatted | 1 mg |
|  | 2.6 mg of powdered almond butter (PB2®) | 1 mg |
| **Cashew** | 1 cashew | 275 mg |
|  | 5.6 mg of cashew meal | 1 mg |
| **Pecan** | 1 pecan half | 150 mg |
|  | 10 mg of pecan meal | 1 mg |
| **Pistachio** | 1 pistachio | 150 mg |
|  | 4.6 mg of pistachio meal | 1 mg |
| **Walnut** | 1 walnut half | 360 mg |
|  | 6 mg of walnut meal | 1 mg |
| **Salmon** | 5 mg of smoked or raw salmon | 1 mg |
| **Sesame** | 1 ml tahini | 167 mg |
|  | 2.5 mg of defatted sesame flour | 1 mg |
|  | **DO NOT USE** whole sesame seeds unless crushed to allow release of the allergen contained in the seed. | |
| **Shrimps** | 1 small shrimp (2.5g) | 450 mg |
| **Soy** | 1 ml soy milk (7g/250 ml) | 28 mg |
| **Oat** | 7.5 mg of oat flour | 1 mg |
| **Barley** | 9.3 mg of barley baby cereal | 1 mg |
| **Rye** | 7.5 mg of rye flour | 1 mg |
| **Buckweat** | 7.5 mg of buckwheat flour | 1 mg |
| **Wheat** | 1 slice of whole wheat bread (32g) | 4380 mg |
|  | 10 mg of white all-purpose flour | 1 mg |
|  | 1 macaroni (elbow) | 35 mg |

## Sample dosing schedules

**Note:** all schedules are meant as guidance and should be adapted to patient response and personal objectives. These are provided as examples to guide implementation of OIT and have not been shown superior to other published schedules.

### CoFAR schedule

This protocol, published by Burks et al in 2012,^32^ consists in an initial food escalation up to a dose of 6 to 50 mg of food protein. Followed by daily dosing with the highest tolerated dose and up-dosing every other week.

Food doses are initially doubled (+100%) at each step up to a dose of 50 mg protein. Percentage increments are then progressively decreased to increments of +25%.

| Step | Protein amount | % increase |
| --- | --- | --- |
| 1 | 0.1 mg | - |
| 2 | 0.2 mg | 100% |
| 3 | 0.4 mg | 100% |
| 4 | 0.8 mg | 100% |
| 5 | 1.5 mg | 100% |
| 6 | 3 mg | 100% |
| 7 | 6 mg | 100% |
| 8 | 12 mg | 100% |
| 9 | 25 mg | 100% |
| 10 | 50 mg | 100% |
| 11 | 75 mg | 50% |
| 12 | 100 mg | 33% |
| 13 | 125 mg | 25% |
| 14 | 156 mg | 25% |
| 15 | 195 mg | 25% |
| 16 | 245 mg | 25% |
| 17 | 306 mg | 25% |
| 18 | 383 mg | 25% |
| 19 | 479 mg | 25% |
| 20 | 599 mg | 25% |
| 21 | 749 mg | 25% |
| 22 | 936 mg | 25% |
| 23 | 1170 mg | 25% |
| 24 | 1463 mg | 25% |
| 25 | 1829 mg | 25% |
| 26 | 2286 mg | 25% |
| 27 | 2858 mg | 25% |
| 28 | 3572 mg | 25% |
| 29 | 4465 mg | 25% |
| 30 | 5582 mg | 25% |
| 31 | 7000 mg | 25% |

### Omalizumab-enabled accelerated schedule

Adapted from Begin et al., 2014^23^

This up-dosing schedule follows the same structure as CoFAR schedule, but faster. The first steps for the initial escalation triple the amount of food (+200%) and latter up-doses consist in doubling the dose (+100%). This accelerated protocol was developed for patients having received omalizumab for at least 2 months prior to initiating OIT, which contributes to increase their reactivity threshold (off-label indication).

| Step | Protein amount | % increase |
| --- | --- | --- |
| 1 | 5 mg | - |
| 2 | 15 mg | +200% |
| 3 | 50 mg | +233% |
| 4 | 150 mg | +200% |
| 5 | 300 mg | +100% |
| 6 | 600 mg | +100% |
| 7 | 1200 mg | +100% |
| 8 | 2400 mg | +100% |
| 9 | 4800 mg | +100% |
| 10 | 9600 mg | +100% |

### Pre-school peanut schedule

This protocol was published in a cohort study of toddlers and pre-schoolers undergoing peanut OIT^9^ but can be used for any patient with high baseline reactivity threshold to their food. It can be initiated with an initial escalation or directly at a dose that is expected to be below the patient’s reactivity threshold (given in clinic). For convenience, the up-dosing steps were developed based on easily measurable portions of the Bamba peanut snack. The schedule should be adapted to the food used to remain convenient.

| Step | Protein amount |  | % increase |
| --- | --- | --- | --- |
| 1 | 10 mg | 1/8 sticks | - |
| 2 | 20 mg | ¼ sticks | 100% |
| 3 | 40 mg | ½ sticks | 100% |
| 4 | 80 mg | 1 sticks | 100% |
| 5 | 120 mg | 1 ½ sticks | 50% |
| 6 | 160 mg | 2 sticks | 33% |
| 7 | 240 mg | 3 sticks | 50% |
| 8 | 320 mg | 4 sticks | 33% |

### Individualized symptom-driven up-dosing

This approach consists of individualizing up-dosing speed according to patient tolerance. There is no predetermined schedule. Home dosing is started at the highest tolerated dose from an initial escalation customized based on patient’s baseline allergy profile (i.e. allergy testing, previous challenges or clinical reactions) and personal tolerance to the risk of reacting on that first introduction.

**Intervals between dosing visits**

The approach generally assumes constant intervals between up-dosing visits (2 weeks to 3 months). In some patients, longer intervals may improve chances of tolerating a given escalation but the extent to which length of interval between up-dosing visits contributes to preparedness for next escalation is unknown and most likely varies between patients. At a minimum, new symptoms from the previous escalation should have abated before proceeding with the next escalation. Greater intervals between intervals can also contribute to decreasing the logistical burden of therapy for patients and providers.

**First up-dosing visit:**

When the starting dose was well tolerated, both in clinic and at home, the first up-dosing visit will usually consist of an attempt to double the dose (+100%). An additional +100% can be added 30 minutes later during the same visit if it was well tolerated, for a total +200% escalation. A more conservative initial increment of +50% can also be considered based on specific context and shared-decision making.

***Example A:*** *First up-dosing after starting OIT at a daily dose of 10mg.*

| Step | Amount | % of current dose | Cumulative |
| --- | --- | --- | --- |
| 1 | 20 mg | +100% | 20 mg |
|  | 10 mg | +100% | 30 mg |

**If the escalation is not tolerated in clinic:** Patient should remain on the current dose and rescheduled for an attempt to escalate with half the failed % increment (+50% in the example above, 15 mg). Alternatively, in absence of systemic symptoms to the failed escalation, the patient could also attempt to increase to half the failed % increment at home.

**Following visits:**

**If the dose was well tolerated at home since the last escalation:**  The subsequent escalation should use the same percent increment.

***Example A****: Escalation to 30 mg (+200%) was well tolerated in clinic and at home.*

| Step | Amount | % of current dose |
| --- | --- | --- |
| 2 | 90 | +200% |
| 3 | 270 | +200% |
| 4 | 2 hazelnuts |  |

**If the escalation was not well tolerated at home after the last escalation:** The subsequent escalation should use half of the % increment

***Example B****: Escalation to 20mg (+100%) was tolerated in clinic but patient was bothered by moderate symptoms during the first week of home dosing at that dose.*

| Step | Amount | % of current dose | Outcome |
| --- | --- | --- | --- |
| 2 | 30 | +50% | Moderate symptoms at home |
| 3 | 38 | +25% | No symptoms |
| 4 | 48 | +25% | No symptoms |
| 5 | 60 | +25% | No symptoms |

**If following escalations are completely symptom-free:**  Re-attempting higher percent increments can be considered. Assessment of dosing tolerance should take into account whether or not pre-medication is used prior to food dosing. Transient mild symptoms with home dosing are frequent in the first week following an escalation and are an indication that treatment speed is most likely optimal.

***Example B (cont’d)***

| Step | Amount | % of current dose | Outcome |
| --- | --- | --- | --- |
| 6 | 90 | +50% | Oral pruritus for 4 days |
| 7 | 135 | +50% | No symptoms* |
| 8 | 200 | +50% | No symptoms* |
| 9 | 300 | +50% | No symptoms* |
| 10 | 2 hazelnuts |  | No symptoms |

*Patient advised to take cetirizine 60 minute before dose for the first 4 days after an escalation.

**When patients are using pre-medication** before taking food dose at home, the same medication should be used prior to the up-dosing. When patients are symptom-free with medication, ability to tolerate the dose without the pre-medication should be assessed before considering further increasing the up-dosing speed.

## Pre-medication

**Note:** The following protocol on the use of pre-medication is meant as an example to help support implementation of OIT and has not been shown superior to other clinical approaches to pre-medication in OIT. Recourse to and choice of mediation should be individualized based on patient specific symptoms and context and should be adapted to patient response and personal objectives. Apart from disodium cromoglycate which is specifically indicated for the prevention of gastro-intestinal IgE-mediated symptoms, the uses described below constitute off-label uses.

**For oral pruritus:**

- Consider a trial of non-sedating oral H1 antagonists one hour before taking the food dose.

**For immediate gastro-intestinal symptoms (<30 minutes)**:

- Consider taking the food dose with along with a bulky meal or snack
- Consider a trial of oral H2 antagonists and/or non-sedating oral H1 antagonists one hour before taking the dose.

**For delayed gastro-intestinal symptoms (>45 minutes)**:

- Consider taking dose in the morning, as sleep cycle/deprivation can contribute to decreased dose tolerance.
- Consider a trial of oral montelukast, one hour before taking the doses^104^.
- Consider taking misoprostol before the dose, once a day. Finding optimal dosage of misoprostol can be challenging as it can cause confusing symptoms of intestinal cramping and diarrhea. Determining the right therapeutic index in the context of OIT requires close follow-up with patient and adjustments. It is usually reserved for patients where delayed symptoms appear to progress rather than abate over time following a food dose increase, presumably from the local cumulative amplifying effect of prostaglandins, and mast cell stabilizers are insufficient or not covered.

**For either immediate or delayed gastro-intestinal symptoms, or both:**

- Consider a trial of disodium cromoglycate no more than 15 minutes before taking the dose (once a day, unless food dose is taken multiple times).

**In the context of an unavoidable co-factor (e.g. NSAIDs following dental procedure), to decrease the risk of systemic reactions to the dose:**

- Consider a transient decrease in the daily dose
- Consider transiently adding disodium cromoglycate, taken once a day, no more than 15 minutes before the food dose. In mice models, disodium cromoglycate prevents the loss of barrier functions resulting from mast cell activation in the gastro-intestinal tract that is exacerbated by co-factors such as NSAIDs, which in turn protected against co-factor induced systemic reactions to the food dose.^105^

**To prevent the occurrence of isolated bronchospasms following food dosing:**

- The presentation of isolated bronchospasm without other systemic manifestation of anaphylaxis following food dosing suggests localized increased sensitivity of mast cells in bronchial tissue.
- If known asthma, reassess control and need to increase inhaled corticosteroids.
- If no asthma, consider diagnosis and consider a trial of inhaled corticosteroids.
- Assess and address potential contribution of environmental allergens.
- Consider performing FeNO. The objective measure of a local inflammatory process with FeNO is particularly useful to confirm the presence of underlying “sub-clinical” asthma when patient has no manifestation of asthma other than with food dosing. It can be helpful to demonstrate the concept to the patient and to monitor response to therapy in this particular context.

**For gastro-intestinal symptoms which timing is not related to food dosing:**

- Consider the possibility of a cellular-mediated allergic reaction to the food allergen. This can be difficult to differentiate from delayed type IgE-mediated reactions. Response to trials with the above-mentioned medications can help distinguish the underlying process.
- Consider adding a proton-pump inhibitor.
- Consider transiently or permanently decreasing the food dose.
- Consider performing endoscopy. In the context of OIT, the presence of eosinophils on endoscopy can result from either recurrent delayed type IgE-mediated reactions or from a cellular-mediated reaction and should therefore not be the sole basis on which to orient management.
- Consider performing an atopy patch test (APT) with the food allergens. Where many patients with IgE-mediated food allergy can present mild infiltrate on APT, a strong reaction to one or more specific food is unusual and can be useful to guide management.
- *For patient with symptoms compatible with eosinophilic oesophagitis (EoE),*
  - Discuss the options of discontinuing or continuing OIT with patient. In a previous cohort of patients with cellular-mediated symptoms with OIT, those with symptoms compatible with EoE were generally able to continue treatment.
  - Consider a trial of swallowed topical corticosteroid after discussing.
- *For patients with symptoms compatible with eosinophilic gastro-intestinal disease (EGID),*
  - Consider discontinuing OIT. In a previous cohort of patients with cellular-mediated symptoms with OIT, those with symptoms compatible with EGID were unable to complete treatment.
  - For patients already receiving another biologic therapy indicated for their asthma, consider switching to dupilumab, which could contribute to a better control of cellular-mediated allergic response in the gastro-intestinal tract^106^.
